# Metastable Protein–Protein Interactions as a Design Principle for PROTACs: Insights from the RIPK1–VHL System

**DOI:** 10.1021/jacsau.6c00260

**Published:** 2026-05-07

**Authors:** Yue Wu, Zhen Zhang, Nina J. Hawkins, Weiping Tang, Xuhui Huang

**Affiliations:** † Department of Chemistry, Theoretical Chemistry Institute, 5228 University of Wisconsin-Madison, Madison, Wisconsin 53706, United States; ‡ Lachman Institute for Pharmaceutical Development, School of Pharmacy, University of Wisconsin-Madison, Madison, Wisconsin 53705, United States; § Data Science Institute, University of Wisconsin-Madison, Madison, Wisconsin 53706, United States

**Keywords:** RIPK1, target protein degradation, PROTAC, rational drug design, Markov state model

## Abstract

Proteolysis-Targeting Chimeras (PROTACs) eliminate disease-relevant proteins by stabilizing short-lived, metastable protein–protein interactions (PPIs) between a target protein and an E3 ligase. These transient encounter complexes are central to PROTAC activity, yet they are difficult to predict and are often invisible to structure-based or AI-only modeling approaches that favor stable, native interactions. Here, we show that explicitly accounting for metastable PPIs provides an effective design principle for rational PROTAC development. Using large-scale molecular dynamics (MD) simulations combined with integrative generalized master equation (IGME) modeling, we mapped the ensemble of metastable PPIs between receptor-interacting serine/threonine-protein kinase 1 (RIPK1) and the von Hippel-Lindau (VHL) E3 ligase, and identified four distinct PPIs suitable for degrader engagement. We find that different PPIs preferentially accommodate different linker architectures, providing a mechanistic explanation for the counterintuitive experimental observation that PROTACs with very short and very long linkers can consistently yield potent degradation. Guided by this insight, we established a virtual screening workflow for linker design and identified the benzylic position on the VHL ligand as a viable linkage site. Rationally designed novel PROTACs featuring this site achieve complete degradation with subnanomolar to low-nanomolar potency. Our results demonstrate that incorporating protein dynamics into degrader design substantially expands the accessible PROTAC design space.

## Graphical abstract

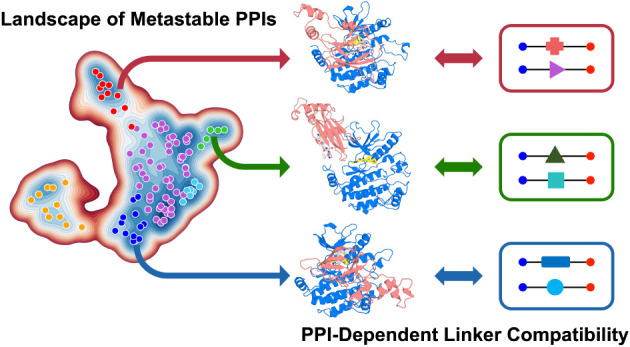

## 1 Introduction

Receptor-interacting serine/threonine kinase 1 (RIPK1) is a central regulator of cellular fate, coordinating upstream signals that direct either pro-survival nuclear factor-κB (NF-κB) signaling or pro-death pathways.^1–4^ Its N-terminal kinase domain facilitates cell-death programs through autophosphorylation,^5–7^ while its intermediate domain mediates scaffolding functions to assemble signaling complexes that regulate pro-inflammatory and pro-survival signaling.^8–12^ Dysregulation of RIPK1 disrupts this balance and is implicated in a range of pathological conditions, including inflammatory and autoimmune disorders as well as cancer.^1,13^ Accumulating evidence indicates that cancer cells can hijack RIPK1’s scaffolding function to divert tumor necrosis factor (TNF) signaling away from cell death and toward NF-κB activation to promote cell survival.^14–16^ In particular, sustained RIPK1-dependent NF-κB activation in cancer cells induces an immunosuppressive chemokine program, leading to reduced T and natural killer (NK) cell infiltration and promoting resistance to immune checkpoint blockade (ICB).^14,17–19^

Traditional small-molecule inhibitors, which target only the kinase domain, cannot suppress this scaffolding function and therefore fail to address RIPK1-driven ICB resistance.^14^ Targeted protein degradation (TPD) offers a promising solution by eliminating the entire RIPK1 protein,^20,21^ thereby disrupting its scaffolding functions and potentially improving responses to immunotherapy. Among TPD strategies, Proteolysis-Targeting Chimeras (PROTACs) have gained particular prominence.^22–25^ PROTACs are bifunctional molecules composed of three components: a ligand that binds the protein of interest (POI), a ligand that recruits an E3 ubiquitin ligase such as von Hippel Lindau (VHL) or Cereblon (CRBN), and a linker that connects the two. By simultaneously engaging the POI and the E3 ligase, PROTACs induce non-native protein–protein interactions (PPIs) and facilitate the formation of a ternary complex. The resulting spatial proximity enables the E3 ligase to catalyze the polyubiquitination of the POI, which subsequently directs the target protein to degradation by the endogenous ubiquitin proteasome system.^26,27^ This event-driven mechanism of action enables PROTACs to operate at substoichiometric doses and provides a powerful strategy for targeting proteins that are difficult to inhibit directly.^28^

Several proof-of-concept studies have demonstrated that RIPK1 can be efficiently degraded by VHL-based PROTACs.^19,29–32^ However, key mechanistic questions remain unresolved, and the effective design space for RIPK1-targeting PROTACs has not yet been systematically explored. Notably, our previous work demonstrated that PROTACs with dramatically different linker architectures can nevertheless achieve highly effective degradation of RIPK1.^31^ Potent degraders were observed across a wide range of linker lengths, spanning from ∼8 Å to ∼16 Å (see Figure 4 and Figure S14). From a static structural viewpoint, such broad linker-length tolerance is difficult to rationalize. This observation suggests that RIPK1 and VHL may engage through multiple, distinct PPIs that can be differentially accessed and stabilized by linkers of varying architectures, a behavior that has also been reported in other PROTAC systems.^33–36^ Compounding this complexity, the VHL ligand binds in a relatively shallow pocket and offers multiple potential attachment sites (Figure 3c), further expanding the combinatorial design space.^37,38^

Yet fully exploiting this rich design space remains challenging, with the key bottleneck being the comprehensive and high-quality modeling of the metastable PPIs involved. Co-crystal structures of ternary complexes are difficult to obtain, and even when available, they capture only static snapshots of what is inherently a dynamic and heterogeneous process.^35,39–41^ As a complement, a wide range of computational methods has also been developed to advance PROTAC discovery.^36,42–44^ Recent deep-learning generative models, such as AlphaFold3 ^45^ or Boltz-2,^46^ have achieved striking success in biological structure prediction. However, because these models are trained predominantly on evolutionarily conserved sequences and experimentally resolved structures, they are optimized to predict native, stable PPIs and struggle to capture the metastable, transient encounter complexes that TPD strategies rely on for productive ternary complex formation.^40,47–49^ Docking approaches remain widely adopted in ternary complex modeling, while their performance depends strongly on conformational sampling, filtering, and scoring methods.^50–56^ In addition, all-atom molecular dynamics (MD) simulations provide a versatile approach, enabling the refinement of docking poses, assessment of conformational stability, and characterization of thermodynamic and kinetic properties of PROTAC ternary systems.^36,39,57–60^ Nevertheless, a systematic and efficient strategy for mapping the full landscape of metastable PPIs in PROTAC systems from MD simulations is elusive.

Markov state models (MSMs) provide a powerful framework for addressing this challenge.^61–75^ MSMs describe conformational dynamics as Markovian transitions among discrete conformational states at fixed time intervals. For an MSM to possess predictive power, it must be constructed from MD simulations long enough for the dynamics to become approximately Markovian. This requirement can limit its applicability to larger systems and longer time scales. To overcome this limitation, the integrative generalized master equation (IGME) method was developed to explicitly incorporate non-Markovian effects through time-integrated memory kernels, enabling reliable modeling even at shorter lag times.^76–78^ In previous work, we introduced a physics-based framework that combines protein–protein docking, large-scale MD simulations, and IGME modeling to evaluate all metastable POI–E3 encounter complexes relevant to PROTAC design for Kirsten rat sarcoma viral oncogene homologue (KRAS).^79^ This enables PROTAC design to be guided by an ensemble of linker-competent encounter complexes rather than by a single static structure.

In this study, we show that explicitly accounting for metastable PPIs enables rational and predictive PROTAC design for RIPK1. Specifically, we used large-scale MD simulations combined with IGME modeling to map the ensemble of metastable PPIs suitable for degrader engagement (Figure 1c–d). Our IGME model revealed four distinct encounter complexes with well-defined geometries, each favoring different linker lengths and attachment sites. Guided by these metastable PPIs, we evaluated linker accessibility across multiple linkage sites and prioritized PROTAC candidates that are compatible with specific PPIs (Figure 1e–f). As demonstrated at the established linkage Site 2 on the VHL ligand,^31^ our results provides a mechanistic explanation for previously puzzling experimental observation that PROTACs with very short (e.g., compound **216–11**, ∼8 Å; Figure 4) and very long (e.g., compound **225–5**, ∼16 Å) linkers can exhibit effective degradation potency, as these linkers stabilize different metastable PPIs. Leveraging this insight, we further identified the benzylic position (Site 3) as a viable but previously underutilized attachment site and rationally designed PROTACs that exploit distinct PPIs. Experimental validation confirmed that both short- (∼7 Å; compound **269–2**, Figure 5) and long-linker PROTACs (∼16 Å; **269–10**) featuring Site 3 attachment achieve complete degradation with subnanomolar to low-nanomolar DC_50_. Our results demonstrate that explicitly incorporating protein dynamics and encounter complex ensembles substantially expands the effective PROTAC design space beyond what can be inferred from static structures alone.

**Figure 1:**
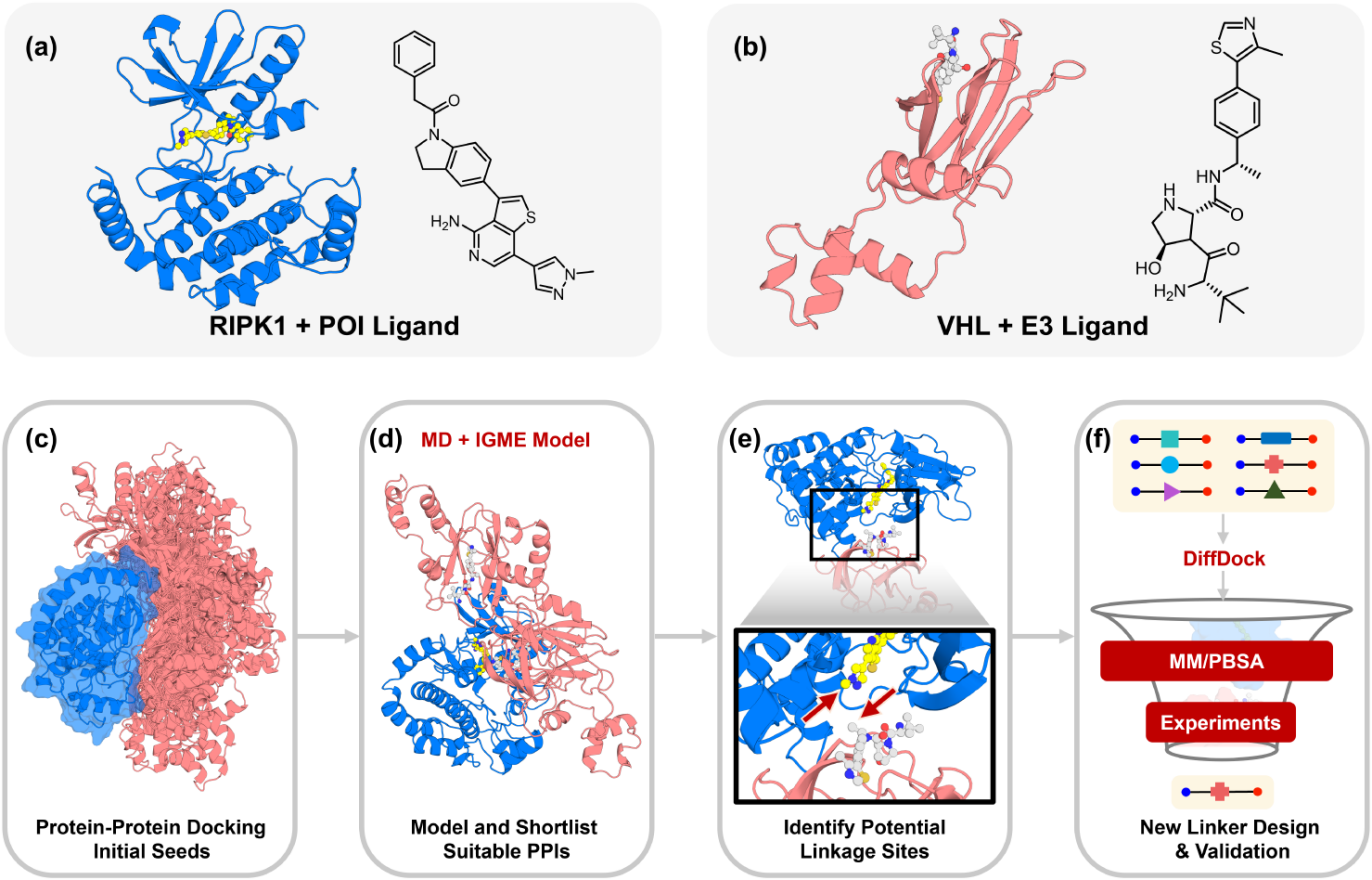
Overall pipeline for the rational design of novel PROTACs targeting RIPK1. (a) Structure of protein-of-interest (POI) RIPK1 and its corresponding POI ligand. (b) Structure of the E3 ligase VHL and its E3 ligand. (c) Rigid protein–protein docking is performed to generate initial seeds for MD simulations. (d) Metastable PPIs are identified by integrating MD simulations with IGME analysis and are subsequently shortlisted. (e) High solvent-exposed linkage sites with favorable orientation and linker length are selected for PROTAC linker construction. (f) DiffDock is employed to model ternary complexes for designed linker candidates, followed by short MD simulations, binding free energy calculations (MM/PBSA), and experimental validation.

## 2 Results and Discussions

### 2.1 IGME Models Reveal Multiple Metastable RIPK1–VHL PPIs

A central question in rational PROTAC design is how many distinct metastable PPIs are accessible between the target protein and the E3 ligase, and whether these PPI geometries can be exploited by different linker designs. For the RIPK1–VHL system, we found that the interaction landscape is not dominated by a single ternary arrangement. Instead, RIPK1 and VHL sample multiple metastable encounter complex PPIs, each with distinct geometries that are potentially compatible with degrader engagement.

To characterize this ensemble, we performed large-scale MD simulations of RIPK1 bound to its ligand GSK’074 ^80^ (Figure 1a) and VHL bound to the ligand Me-VH032 ^81,82^ (Figure 1b). As a type II inhibitor, GSK’074 stabilizes the DLG-out inactive state of RIPK1 and prevents transition to the active conformation.^80,83^ Accordingly, our simulations exclusively sample encounter complexes associated with the inactive state. The linker was intentionally omitted to allow the two proteins to freely explore relative orientations relevant to ternary complex formation. Initial encounter configurations were generated by rigid-body protein–protein docking using the HDOCK program^84^ and served as starting points for extensive MD simulations. In total, approximately 420 μs of all-atom MD trajectories were accumulated on the Folding@home^85,86^ platform for a system containing 188,802 atoms (see Methods, SI text Section 1, and Figure S1 for details).

Analysis of these MD trajectories shows that the relative motion between RIPK1 and VHL is highly heterogeneous and dominated by interprotein rearrangements rather than internal conformational changes. To capture this behavior, we focused on interprotein Cα–Cα distances as input features to describe the PPI formation. After feature selection^87^ and dimensionality reduction using time-lagged Independent Components Analysis (tICA),^88–90^ the MD conformations were clustered^91^ into 100 microstates and further lumped^92,93^ into six metastable states (Figure 2a). These metastable states correspond to distinct encounter complex PPIs with characteristic interface geometries (see Figure 2d, Figures S2–S3, and SI text Section 2.3 for details).

**Figure 2:**
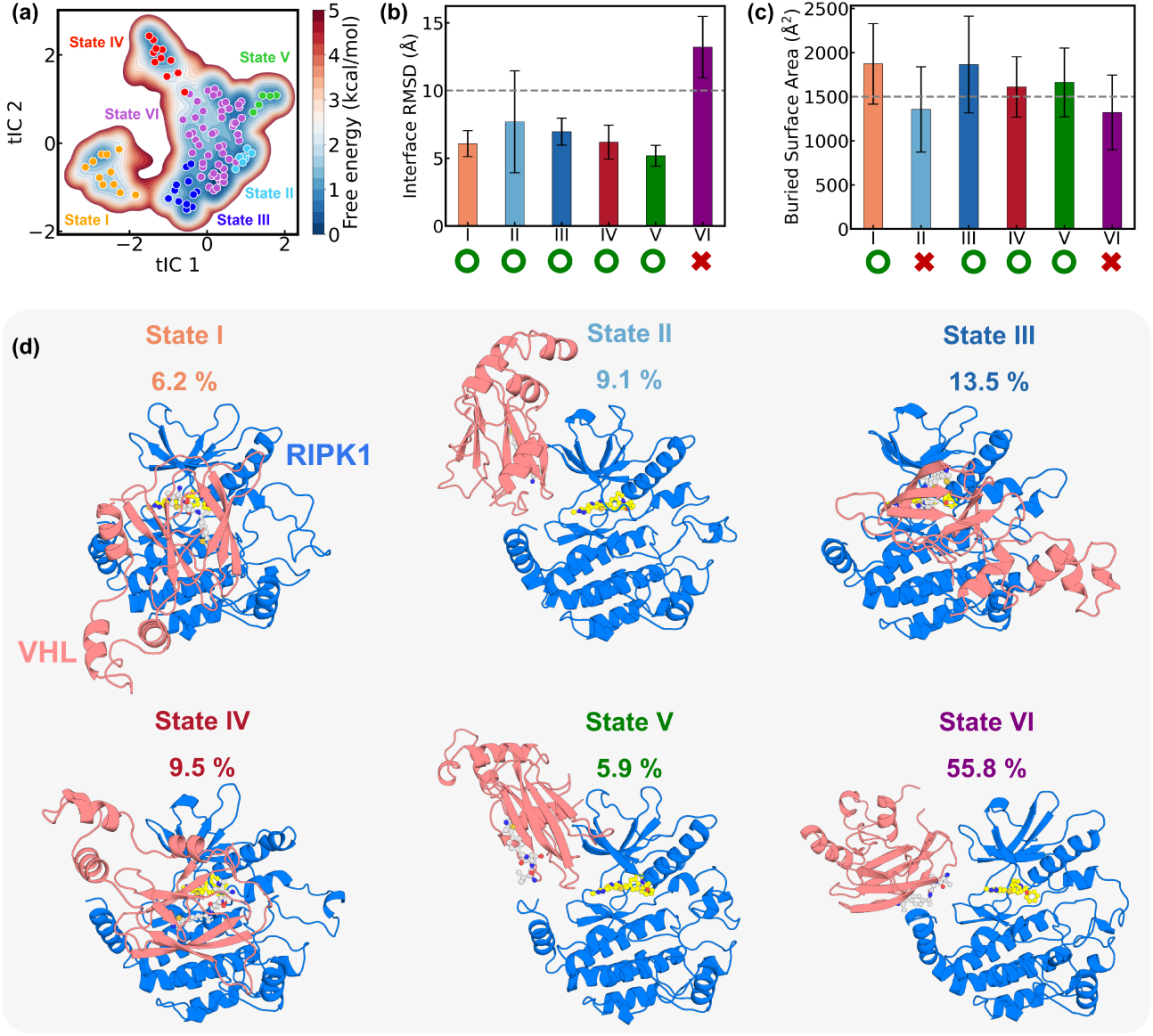
Metastable protein–protein interfaces between RIPK1 and VHL identified from the IGME model. (a) Free energy landscape projected onto the first two tICA components. Microstate centers are shown as dots: state I (orange), state II (cyan), state III (blue), state IV (red), state V (green), and state VI (purple). (b) Interface RMSD values of the six metastable states, with mean and standard deviation indicated. (c) Buried surface area (BSA) of the six metastable states, with mean and standard deviation indicated. (d) Representative structures and populations of the six metastable states. RIPK1 is shown in marine and VHL in salmon. Populations are mean values derived from the top 5% IGME models. The selected structures correspond to the centers of the most populated microstates for each metastable state.

Conventional Markov state models (MSMs)^61–75^ constructed for this system were unable to accurately reproduce the long-time scale dynamics for the six metastable states. In particular, the 6-state MSM failed the Chapman–Kolmogorov test at a lag time of 150 ns (Figure S4 d), indicating pronounced non-Markovian dynamics in RIPK1–VHL encounter complex formation. To address this challenge, we applied the IGME framework, which explicitly incorporates dynamic memory effects via a time-integrated memory kernel.^76–78^ For this system, we build IGME models using trajectory data with lag times <100 ns. As shown in Figure S4 b, IGME models constructed across a broad range of hyperparameters yielded consistently low root-mean-square error (RMSE) values, with the top 5% of models reaching mean RMSE values as low as 2 × 10^–4^. This represents a substantial improvement over the MSM in Figure S4 d, which exhibited an RMSE of approximately 1 × 10^–3^. Importantly, IGME models successfully passed the Chapman–Kolmogorov test, confirming their ability to capture the dynamics of RIPK1–VHL encounter complex formation as shown in Figure S4 d.

Based on the validated IGME analysis, we quantified the thermodynamic populations and kinetic stability of the six metastable states (Figure 2d and Figure S5). All six states are populated to a substantial extent (>5%), indicating that RIPK1 and VHL can access multiple encounter complex geometries on biologically relevant time scales.

### 2.2 Shortlist Suitable PPIs for Further Linker Design

The presence of multiple metastable PPIs provides an advantage for degrader design, as different encounter complexes offer distinct geometries that can be selectively stabilized by different PROTAC linkers. However, not all metastable PPIs are equally suitable for rational linker design. To identify encounter complex PPIs that are likely to support productive ternary complex formation, we evaluated the six metastable RIPK1–VHL PPIs using a combination of criteria, including structural heterogeneity, binding modes, and interfacial burial.

We first examined the structural features of the six metastable states identified by the IGME model (Figure 2d). Interprotein Cα–Cα distance maps (Figure S6) reveal that each state adopts a distinct PPI geometry with substantial variation in spacing between RIPK1 and VHL. States I and III exhibit compact interaction patterns with extensive close contacts, whereas State VI displays only a few localized regions of proximity. Residue-level contact frequency analysis (Figure S7) further shows that each interface is stabilized by a unique subset of residues. Further analysis by residue type (Figure S9) shows that charged and polar residues contribute a larger fraction of interprotein contacts than hydrophobic residues in both RIPK1 and VHL, indicating that electrostatic interactions play a major role in stabilizing these encounter complexes. In particular, frequent contacts between Arg residues in VHL and Glu residues in RIPK1 suggest that salt bridges play an important role in stabilizing several of the encounter complexes (Figure S9).

Although all six states are populated above 5% and are therefore sufficiently metastable to be potentially stabilized by PROTACs, additional filtering is required to identify PPIs suitable for rational linker design.^79^ We assessed the structural heterogeneity within each state by computing the interface root-mean-square deviation (interface RMSD) using the centers of the constituent microstates as references. As shown in Figure 2b, five states (States I to V) exhibit moderate interface RMSD values, indicating relatively well-defined and stable PPI interfaces. In contrast, State VI displays a substantially higher interface RMSD (>10 Å), reflecting a broad distribution of diverse binding modes and high configurational entropy. Overlay of representative structures from State VI (Figure S10) further confirm pronounced conformational variability, suggesting that this state is too heterogeneous to serve as a reliable PPI for linker design.

We next evaluated the buried surface area (BSA) of each metastable PPI, as larger interfacial BSA in PROTAC-mediated ternary complexes has been shown to correlate with stronger binding and higher degradation potency.^94,95^ The BSA was calculated by subtracting the solvent-accessible surface area (SASA) of the individual proteins from that of the encounter complex. As shown in Figure 2c, States II and VI exhibit significantly smaller BSA values than the remaining states. This observation is consistent with the smaller number of residues exhibiting high contact frequency (Figure S8), indicating weaker or more transient interaction patterns. Based on the above criteria, we selected States I, III, IV, and V as the most promising PPIs for subsequent PROTAC linker design.

### 2.3 Identifying Viable Linkage Sites across Metastable RIPK1–VHL PPIs

Rational PROTAC design requires the identification of chemically viable linkage sites on both the POI ligand and the E3 ligand that are compatible with the geometry of specific encounter-complex PPIs. Our analysis shows that linker placement is strongly constrained on the RIPK1 ligand but considerably more flexible on the VHL ligand, creating multiple viable linkage sites that can be selectively exploited across different metastable PPIs.

To quantify linker feasibility, we first evaluated solvent exposure by calculating the solvent-accessible surface area (SASA) of all heavy atoms in the RIPK1 ligand GSK’074 (Figure 3a) and the VHL ligand Me-VH032 (Figure 3b, see Methods and SI text Section 4 for details). A clear contrast emerges between the two ligands. GSK’074 is deeply buried within the RIPK1 kinase pocket and exhibits uniformly low SASA values. Ranking individual heavy atoms by SASA (Figure S11) reveals that only the methyl group attached to the solvent-exposed pyrazole ring exceeds 10 Å^2^, identifying it as the sole feasible attachment point on the RIPK1 ligand (denoted Site C). In contrast, Me-VH032 binds in a relatively shallow and solvent-exposed region of VHL, providing substantially greater flexibility for linker installation. Consistent with prior experimental studies,^38,96^ five exit vectors on the VHL ligand are viable for PROTAC design (Figure 3c): Site 1 at the left-hand-side (LHS) amide, Site 2 on the *tert-*leucine group, Site 3 at the benzylic (*S*)-methyl group, Site 4 on the phenyl ring, and Site 5 on the thiazole group. Across the shortlisted metastable PPIs, these five sites generally exhibit SASA values above 10 Å^2^, with the exception of Site 2 in States I and IV, where steric occlusion reduces solvent exposure (Figure S11).

**Figure 3:**
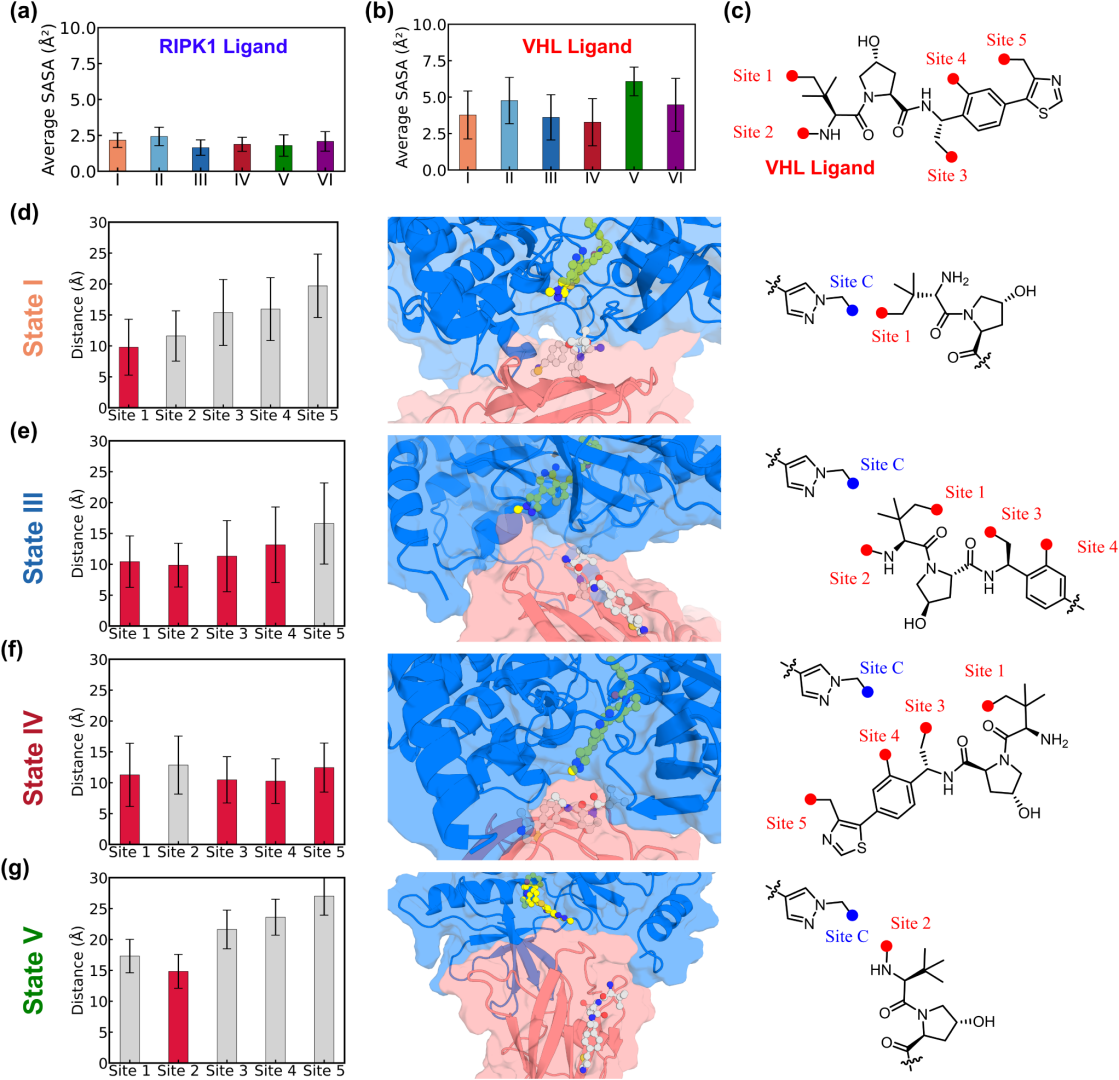
Identification of potential linkage sites for shortlisted metastable states. (a) Average SASA of the RIPK1 ligand heavy atoms. (b) Average SASA of the VHL ligand heavy atoms. (c) Most frequently observed linkage sites on VHL ligands used for PROTAC design. (d–g) Suitable linkage sites for state I (d), state III (e), state IV (f), and state V (g). *Left:* Distances between the linkage sites on the VHL ligand (Sites 1–5) and the methyl carbon of the pyrazole ring (Site C) on the RIPK1 ligand. Red bars indicate sites suitable for linker design, whereas gray bars indicate unsuitable sites. *Middle:* Close-up views of the relative positions of the protein complex and ligands, with the protein surface shown. The structures correspond to the centers of the most populated microstates for each metastable state. *Right:* Identified optimal linkage sites for each metastable state.

Beyond solvent accessibility, geometric distance imposes an additional constraint on linker design. Excessively long linkers are generally disadvantageous, as they increase the conformational entropic penalty during ternary complex formation and often compromise drug-like properties by increasing molecular weight, resulting in reduced cell permeability and enhanced metabolic vulnerability.^97–99^ Accordingly, we measured the distances between Site C on GSK’074 and each of the five potential linkage sites on Me-VH032 and considered a mean distance below 15 Å to be optimal for linker construction. For State I (Figure 3d), Sites 1 and 2 fall within the preferred distance range. However, Site 2 is sterically occluded by the *tert*-butyl group, as reflected by both structural inspection and reduced SASA. Sites 3 and 4 lie slightly above the upper distance threshold and require highly contorted linker paths, rendering them unfavorable. As a result, Site 1 emerges as the only viable attachment point for this encounter complex PPI (State I). In State III (Figure 3e), four sites (Sites 1–4) satisfy both solvent exposure and distance criteria. Sites 1–3 adopt favorable orientations that allow direct and geometrically reasonable linker paths from Site C. Although Site 4 presents a modest steric constraint due to the benzylic methyl group, it remains chemically tractable, particularly given the availability of the methyl-free analog VH-032. In State IV (Figure 3f), all five sites fall within an acceptable distance range; however, as in State I, Site 2 is sterically hindered and exhibits average SASA values below 10 Å^2^, leaving the remaining four sites as viable candidates. In contrast, for State V (Figure 3g), only Site 2 lies within the optimal distance range and adopts a favorable orientation relative to Site C, making it the preferred attachment point for this state.

Our above analysis demonstrates that different metastable RIPK1–VHL encounter complexes impose distinct geometric constraints on linker placement. Rather than a single universal exit vector, multiple linkage sites on the VHL ligand can be selectively matched to specific PPIs, thereby expanding the accessible design space for PROTAC development.

### 2.4 Distinct Metastable PPIs Preferentially Accommodate Short and Long Site-2 Linkers

A counterintuitive observation in PROTAC design is that compounds bearing very different linker lengths can exhibit similarly high degradation potency. For RIPK1-targeting PROTACs, both short and long Site-2 linkers have been reported to achieve strong degradation, despite their markedly different geometries.^31^ Our metastable-PPI framework provides a mechanistic explanation for this behavior: distinct RIPK1–VHL encounter complexes impose different geometric constraints and therefore preferentially accommodate different linker lengths.

Site 2 (the LHS amide on the VHL ligand; Figure 4a) is the most widely used attachment point in VHL-based PROTACs and thus offers an ideal testbed for examining this principle.^38^ Among the metastable PPIs identified in Section 2.2, States III and V provide favorable geometry and orientation for Site-2-based linkage. Importantly, these two encounter complex PPIs differ substantially in their interprotein arrangement, suggesting that they may favor different linker architectures. Shorter linkers are expected to be more compatible with compact PPIs, whereas longer linkers may be required to bridge more extended encounter geometries.

**Figure 4:**
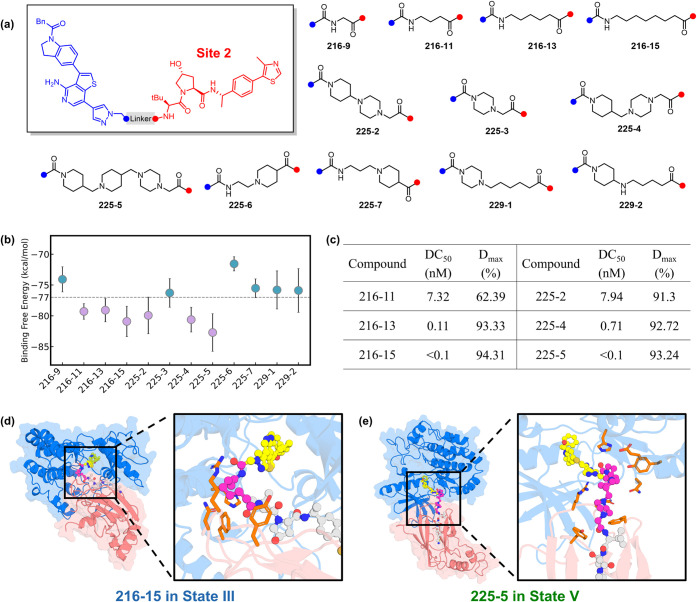
Site 2 linkers for PROTAC design. (a) Chemical structures of PROTACs containing various Site 2 linkers. The RIPK1 ligand and its attachment site are shown in blue, while the VHL ligand and its corresponding attachment site are shown in red. (b) Binding free energies calculated using MM/PBSA for PROTAC molecules shown in (a). The top 50% compounds are in violet while the others are in teal. Mean values and standard deviations are obtained via bootstrapping. The threshold to distinguish top class and bottom was set as −77 kcal/mol. (c) Degradation efficiency of the top-class compounds, as reported in Zhang et al.^31^ DC_50_ represents the dose that reduces RIPK1 protein levels by 50%, and *D*_max_ denotes the maximum reduction of RIPK1 protein achieved by the compound. (d) Structures of **216–15** in State III. (e) Structures of **225–5** in State V. RIPK1 is in marine, VHL in salmon, POI ligand in yellow stick-and-ball, E3 ligand in gray stick-and-ball. Linker motif is in magenta stick-and ball, and residues that have contacts with linkers are shown in orange sticks.

To realize this insight and prioritize viable linker designs, we implemented a multistep virtual screening workflow to estimate the ternary complex stability across candidate PROTAC molecules. We estimate ternary complex stability using the free energy of PROTAC binding to preformed metastable PPIs, because the protein–protein association free energy is fixed for a given PPI (see Methods and SI text Section 5 for details). The workflow integrates metastable PPI structures, PROTAC docking using DiffDock,^100^ MD relaxation, and binding free-energy calculations using the Molecular Mechanics/Poisson–Boltzmann Surface Area (MM/PBSA) method.^101,102^ We evaluated a series of Site-2 PROTACs spanning a range of linker lengths and chemical compositions (Figure 4a) by docking them into representative structures from States III and V. For linkers compatible with both states, the lower-free-energy conformation was retained for analysis (Figure S13). Compounds were then ranked by predicted binding free energy, and the top 50% were selected as candidates with the highest likelihood of productive ternary complex formation. As shown in Figure 4b, a threshold of −77 kcal/mol cleanly separates this upper tier from lower-performing designs.

Strikingly, this physics-informed prioritization correlates strongly with experimental degradation data. Among the top 50% of compounds, five out of six exhibit *D*_max_ values above 90%, and all display DC_50_ values below 10 nM (Figure 4c). In contrast, compounds in the lower-ranking half show substantially weaker degradation activity: none reach *D*_max_ greater than 90%, and only a single compound achieves a DC_50_ below 10 nM (Figure S14). These results demonstrate that binding free-energy evaluation within the appropriate metastable PPI context provides a practical and predictive criterion for identifying effective Site-2 PROTAC linkers.

Further analysis of representative low-free-energy poses provides a mechanistic explanation for the experimental observation that Site-2 PROTACs with short and long linkers can exhibit similarly high degradation potency. In State III, which adopts a more compact encounter geometry, shorter Site-2 linkers are preferentially accommodated. For example, in the poses with favorable binding free energies of compound **216–15**, the linker adopts a slightly bent conformation that fits naturally within the State III interface and forms stabilizing contacts with residues H102 in RIPK1 and N67, R69, F91, and Y112 in VHL (Figure 4d). In contrast, State V presents a more extended PPI geometry that disfavors short linkers but can readily accommodate longer ones. In the low-free-energy poses of compound **225–5**, the extended linker spans the larger interprotein separation and engages additional contacts with Y308, E311, and N312 of RIPK1 (Figure 4e).

The above results demonstrate that Site-2 linker length is not governed by a single “optimal” value. Instead, short and long linkers can both be highly effective when they stabilize different metastable RIPK1–VHL encounter complexes. This finding resolves the apparent paradox of linker-length tolerance in RIPK1 PROTACs and underscores a general design principle: effective degraders arise from matching linker geometry to the specific metastable PPI they preferentially stabilize, rather than from optimization against a single static ternary structure.

### 2.5 An Underutilized Benzylic Attachment Site Enables Potent Degradation via Distinct Metastable PPIs

Beyond rationalizing linker-length tolerance at the established Site 2, our virtual screening workflow enables the systematic identification of an underutilized attachment site at the benzylic position on the VHL ligand (Site 3). Although this position has been explored in a limited number of prior studies,^32^ it has not been widely adopted, in part because a principled framework for matching linker geometry to compatible protein–protein encounter complexes has been lacking.

Our analysis indicates that two metastable RIPK1–VHL encounter complexes, States III and VI, provide suitable but distinct PPI geometries for Site-3-based linkage (Figure 3e–f). To test this hypothesis, we evaluated a series of Site-3 PROTACs spanning a broad range of linker lengths (Figure 5a). Binding free-energy calculations reveal a clear, state-dependent preference (Figure S15). In State VI, which adopts a compact encounter complex geometry, the shortest linker, compound **269–2** (∼7 Å), is strongly favored, exhibiting an average binding free energy below −77 kcal/mol (Figure 5b). In contrast, for State III, progressively longer linkers yield increasingly favorable binding free energies (Figure S15 b), with compounds **269–8** and **269–10** achieving the most stable binding.

**Figure 5:**
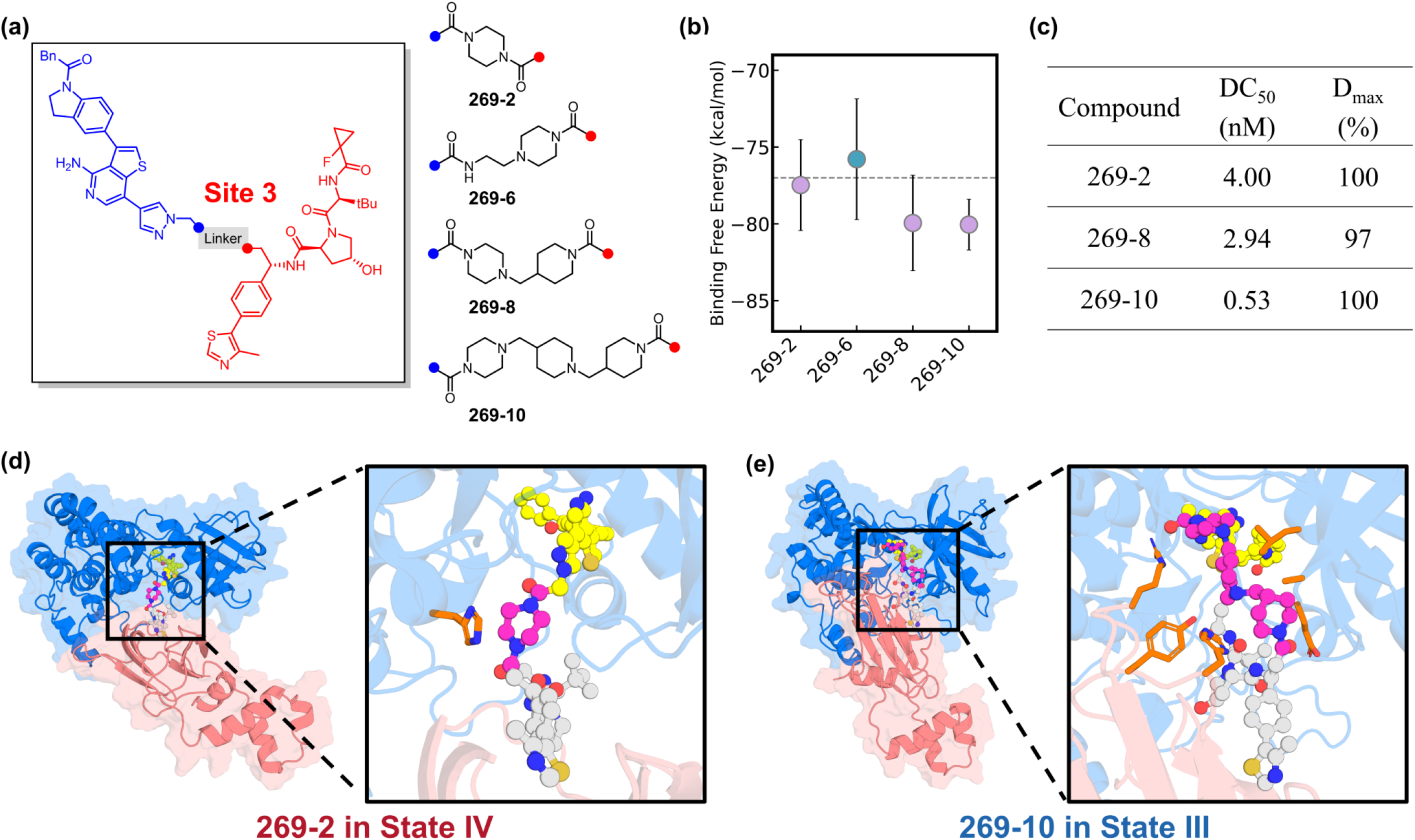
Site 3 linkers for PROTAC design. (a) Chemical structures of PROTACs containing various Site 3 linkers. The RIPK1 ligand and its attachment site are shown in blue, while the VHL ligand and its corresponding attachment site are shown in red. (b) Binding free energies of the PROTAC molecules shown in (a), calculated using MM/PBSA. Mean values and standard deviations are obtained via bootstrapping. (c) Degradation activity of the PROTAC molecules in (a), evaluated by measuring RIPK1 protein levels using Western blot analysis. (d) Structures of **269–2** in State IV. (e) Structures of **269–10** in State III. RIPK1 is in marine, VHL in salmon, POI ligand in yellow stick-and-ball, E3 ligand in gray stick-and-ball. Linker motif is in magenta stick-and ball, and residues that have contacts with linkers are shown in orange sticks.

We next experimentally prepared a new library of compounds and tested these predictions (see Methods, SI text Sections 6–7, and Figure S16 for details). Consistent with the computational analysis, all three prioritized Site-3 PROTACs, **269–2**, **269–8**, and **269–10**, exhibited potent RIPK1 degradation in cellular assays (Figure 5c). Each compound achieved *D*_max_ values above 90% and DC_50_ values below 10 nM. Notably, both the shortest linker (**269–2**, ∼7 Å) and the longest linker (**269–10**, ∼16 Å) achieved complete degradation (*D*_max_ = 100%), with **269–10** displaying subnanomolar potency. Although **269–2** is modestly less potent than **269–10**, its minimal linker length reduces overall molecular weight, offering potential advantages in synthetic accessibility and pharmacokinetic properties.

To further evaluate the potential off-target effects of the Site-3 degraders, we treated PC3 cells with compound **269–10** across a range of concentrations and assessed the protein levels of RIPK1, RIPK3, and phosphorylated mixed lineage kinase domain-like pseudokinase (p-MLKL). As shown in Figure S17, no significant changes were observed in the levels of RIPK3 or p-MLKL, indicating minimal off-target effects on these related signaling proteins and confirming that RIPK1 degradation alone does not significantly affect downstream necroptosis. We next examined the degradation mechanism by pretreating PC3 cells with either the free RIPK1 ligand, VHL ligand, proteasome inhibitor MG132,^103^ or the neddylation inhibitor MLN4924 ^104^ prior to PROTAC treatment. As shown in Figure S18, all pretreatments effectively rescued RIPK1 levels, demonstrating that degradation requires both target and E3 ligase engagement and proceeds through a VHL-dependent ubiquitin–proteasome pathway.

Structural inspection of low-free-energy poses provides a mechanistic explanation for the high degradation efficiency observed with degraders featuring markedly different Site-3 linker lengths. In State VI, the compact geometry allows compound **269–2** to fit snugly within the protein–protein interface, where the piperazine ring engages stabilizing hydrophobic contacts with RIPK1 residue H102 (Figure 5d). In contrast, State III readily accommodates longer linker architectures. In the low-free-energy poses of **269–10**, the extended linker forms additional interactions with RIPK1 residues A21, E22, L33 as well as VHL residues R69, H110, and Y112 (Figure 5e). Thus, as observed for Site 2, short and long Site-3 linkers succeed by stabilizing different metastable RIPK1–VHL encounter complexes.

The above results establish that the benzylic Site 3, while previously underutilized, can be robustly and predictively exploited when metastable PPIs are explicitly considered. More broadly, they demonstrate the generality of our design principle: effective PROTACs emerge from matching linker geometry and attachment site to specific metastable encounter complex PPIs, rather than from optimization against a single static ternary structure. The design space enabled by this perspective extends beyond the experimentally validated Sites 2 and 3. Other attachment sites on the VHL ligand, particularly Sites 1 and 4, may offer additional opportunities to exploit multiple encounter complexes and further enhance degrader success rates. More broadly, as our physics-based design framework does not rely on VHL-specific structural features, it can potentially be extended to other E3 ligases, including CRBN, provided that suitable initial structures are available. We therefore anticipate that integrating metastable-PPI analysis with advances in chemical synthesis and experimental screening will provide a general and transferable strategy for PROTAC discovery across diverse protein–E3 ligase systems.

RIPK1 participates in several signaling complexes with distinct cellular functions.^1–4^ In the tumor necrosis factor receptor 1 (TNFR1) signaling pathway, RIPK1 functions as a scaffold in complex I to promote pro-survival signaling, whereas activation of RIPK1 can contribute to the formation of cell-death complexes such as complex IIa and IIb. These distinct complexes raise the question of whether degrader design could in principle exploit different RIPK1 conformations or RIPK1–VHL encounter complexes to achieve pathway-selective degradation. However, such selectivity is unlikely to arise solely from engagement of the kinase domain. Assembly of these signaling complexes, along with RIPK1 oligomerization, is primarily mediated by interactions involving the intermediate and death domains,^4,6,83,105–107^ whereas the PROTACs developed here bind the kinase domain. Therefore, these higher-order assemblies are not expected to directly interfere with VHL recruitment. Moreover, kinases are known to dynamically interconvert between active and inactive conformational states under physiological conditions,^108,109^ allowing degraders that preferentially bind one state to access the broader RIPK1 population. Consistent with this view, our type II inhibitor-based degraders (e.g., **269–2** and **269–10**) achieve complete degradation (*D*_max_ ≈ 100%), indicating that conformational preference does not restrict degradation to a particular conformational state of RIPK1.

Recent advances in deep learning based generative models, exemplified by AlphaFold3 ^45^ and Boltz-2 ^46^, are transforming structure prediction of complex biological assembly. By leveraging evolutionary sequence information and experimentally resolved structures, these tools excel at modeling native, stable PPIs. However, PROTAC-mediated degradation relies on stabilizing metastable, short-lived encounter complexes that are not typically represented in the training data of such models. As a result, direct application of these methods to PROTAC design can yield heterogeneous or weak ternary complexes. To examine this limitation in the RIPK1–VHL system, we applied Boltz-2 ^46^ as a representative generative model to Site-3-based PROTACs with short (**269–2**, ∼7 Å) and long (**269–10**, ∼16 Å) linkers. For the short-linker compound **269–2**, Boltz-2 predicts relative RIPK1–VHL orientations broadly consistent with those identified by IGME modeling. However, the resulting ternary complexes remain heterogeneous and exhibit significantly smaller BSA and fewer interprotein contacts than the corresponding IGME-derived complexes (Figure S19a–e). In contrast, for the long-linker compound **269–10**, Boltz-2 consistently predicts a single relative RIPK1–VHL orientation that differs from the metastable PPI identified by IGME. Despite this apparent structural consistency, all Boltz-2–predicted complexes for **269–10** display substantially reduced BSA (∼604 Å^2^ v.s. ∼1766 Å^2^ in the corresponding IGME models) and fewer interprotein contacts, with representative structures exhibiting markedly diminished interfacial contact regions (Figure S19f–j). These results indicate that AlphaFold-like generative models, when applied directly, may not capture the metastable PPIs required for productive degrader function. In contrast, our physics-informed framework explicitly resolves the dynamic ensemble of metastable encounter complexes that define the effective design space for PROTACs, enabling rational linker selection beyond what can be inferred from static or AI-only structure prediction.

Finally, we note that ternary complex stability alone does not fully determine degradation efficiency. Additional factors, including the proximity and orientation of lysine residues on the POI relative to the ubiquitination machinery, as well as downstream cellular processes, also contribute to degradation outcomes. In this context, our virtual screening workflow is best viewed as an efficient and predictive strategy for prioritizing PROTAC candidates that are structurally and dynamically compatible with productive PPIs, thereby focusing experimental efforts on the most promising designs.

## 3 Conclusion

In this study, we show that explicitly accounting for metastable, short-lived PPIs provides a powerful framework for rational PROTAC design. By mapping the ensemble of metastable encounter complexes between RIPK1 and the VHL E3 ligase, we demonstrate that distinct PPIs impose different geometric constraints and therefore preferentially accommodate linkers of different lengths and orientations. This insight explains the surprising experimental observation that both short and long linkers can achieve similarly optimal degradation potency, as demonstrated at the established Site 2. Guided by this principle, we further performed virtual screening to show that an underutilized benzylic attachment site on the VHL ligand (Site 3) can be robustly exploited when metastable PPIs are explicitly considered. Experimental validation using a newly designed and synthesized PROTAC library confirmed that both short- (compound **269–2**) and long-linker PROTACs (**269–10**) featuring Site 3 attachment achieve complete degradation with subnanomolar to low-nanomolar potency. Altogether, our results establish metastable encounter complexes, rather than a single static ternary structure, as design targets and provide a general, physics-informed strategy for expanding the accessible design space and improving success rates in PROTAC discovery.

## 4 Methods

### 4.1 All-Atom MD Simulations

The VHL structure was obtained from PDB 5NVW^110^ and the RIPK1 structure from PDB 4NEU.^111^ The RIPK1-GSK’074 binary complex was generated by molecular docking using AutoDock Vina^112^ via the DockingPie PyMOL plugin.^113^ Initial structures of MD simulations were produced by rigid protein–protein docking with HDOCK^84^ with a 30 Å distance restraint between the two ligands. MD simulations employed the Amber14SB^114^ force field for proteins with TIP3P water,^115^ and ligand parameters were derived from GAFF2.^116^ Ligand geometries were optimized at the B3LYP/6–311G­(d,p) level with D3BJ dispersion correction^117^ using Gaussian 16,^118^ and electrostatic potentials were computed at the HF/6–31G* level. RESP charges were generated with AmberTools23,^119^ and bonded and Lennard-Jones parameters were assigned from GAFF2. Each system was solvated in a 14 nm dodecahedral TIP3P water box with a minimum 15 Å buffer. Na^+^ and Cl^–^ ions were added to neutralize the system, achieving an ionic strength of 0.15 M. The simulation box contains 188,802 atoms. Long-range electrostatics were treated using PME,^120^ with a 12 Å cutoff for short-range nonbonded interactions. Equilibration simulations were performed in GROMACS 2022.5,^121^ including 10,000 steps of steepest-descent energy minimization followed by 1 ns NVT and 1 ns NPT equilibration with positional restraints on all heavy atoms (1000 kJ mol^–1^ nm^–2^). Bonds involving hydrogens were constrained using LINCS.^122^ During equilibration, temperature was maintained at 300 K using a velocity-rescaling thermostat^123^ (τ = 0.1 ps), and pressure was controlled at 1 bar using a Berendsen barostat^124^ (τ = 0.5 ps). Production simulations were conducted in OpenMM 8.0.0 ^125^ under the NVT ensemble using a Langevin middle integrator (2 fs time step, friction coefficient 1.0 ps^–1^). After 10 ns of local simulations, final structures were used to initiate large-scale Folding@Home^85,86^ simulations. The final data set comprised trajectories from 31 seeding structures totaling approximately 420 μs. Please refer to SI text Section 1 and Figure S1 for more details of system setup and all-atom simulations.

### 4.2 Microstate MSM Construction and Validation

Cα–Cα pairwise distances between VHL and RIPK1 were used as input features. All VHL Cα atoms and every other Cα atom from RIPK1 were included, yielding 22,022 features. Spectral Accelerated Sequential Incoherent Selection (spectral oASIS)^87^ was then used to select the 2,000 most informative features (Figure S2a). These features were further projected using tICA^88–90^ to identify slow collective variables (CVs) by maximizing time-lagged autocorrelation. Conformations were then clustered into microstates using the K-means clustering algorithm.^91^ Hyperparameters including the number of CVs, tICA lag time, and number of microstates were optimized via cross-validation using the generalized matrix Rayleigh quotient (GMRQ)^126^ score. Based on this analysis, a tICA lag time of 30 ns, four CVs, and 100 microstates were selected (Figure S2b–d). The resulting microstate MSM model was validated using implied time scale and Chapman–Kolmogorov tests (Figure S3). Please refer to SI text Section 2 for additional details.

### 4.3 IGME Model Construction and Validation

Using the validated microstate MSM, the 100 microstates were further lumped into 6 metastable states using Robust Perron Cluster Analysis (PCCA+).^92,93^ Six metastable states were selected because it yielded relatively low Root Mean Square Error (RMSE, see eq S7) in the IGME models (Figure S4 a). For IGME construction, the two constant matrices, ***A*** and ***T̂*** (eq S6), were numerically fitted to MD data using a least-squares fitting (LSF) approach.^76^ A systematic search over the hyperparameters ( $\tau_{K}^{\mathrm{trial}}$ and *L*) was then performed to identify optimal models that minimized the prediction RMSE (Figure S4b). The top 5% of models with the lowest RMSE values were used to estimate ***T̂*** and characterize the dynamics and thermodynamics of the system. Please refer to SI text Section 3 for additional details.

### 4.4 PROTAC Modeling and Binding Free Energy Calculation

A “core region” was defined within each metastable state by selecting microstates with stationary populations greater than 1%. From each core region, 200 MD snapshots were randomly selected. Ligands were removed from these structures to generate a VHL–RIPK1 receptor ensemble for docking. For each PROTAC molecule compatible with the geometric constraints of a given metastable state (including linker length and attachment sites), DiffDock^100^ was performed against the corresponding 200 receptor structures using default parameters. Docked poses were ranked by ligand RMSD calculated without ligand superposition, and the five lowest-RMSD poses for each PROTAC-state pair were selected for a 10 ns all-atom MD simulation. The production simulations were carried out in the NPT ensemble using the velocity-rescaling thermostat^123^ (τ = 0.1 ps, 300 K) and the Parrinello–Rahman barostat^127^ (τ = 2 ps, 1 bar) in GROMACS 2022.5^121^,. The final 5 ns of each trajectory were used for binding free energy calculations with gmx_MMPBSA tool^102^ based on the MM/PBSA method.^101,102^ Ensemble-averaged binding free energies and uncertainties were estimated by bootstrap resampling of the five trajectories: for each of 10 bootstrap iterations, trajectories were resampled with replacement and averaged, and the final binding free energy and statistical error were taken as the mean and standard deviation across bootstrap sets. To maintain consistency across systems, the fluorocyclopropyl group at Site 3 was excluded from simulations and energy calculations. For compounds **269–2** and **269–10**, additional ternary complex structures were generated using Boltz-2^46^ cofolding with the same protein sequences used in MD simulations. Five structures were generated per compound using default settings. Please refer to SI text Section 5 for more details of DiffDock and Boltz-2 modeling, and the calculation of binding free energies.

### 4.5 Chemical Synthesis

All reactions were conducted under a positive pressure of dry argon in glassware that had been oven-dried prior to use. Anhydrous solutions of reaction mixtures were transferred via an oven-dried syringe or cannula. All solvents were dried prior to use unless noted. Thin-layer chromatography (TLC) was performed using precoated silica gel plates. Flash column chromatography was performed with silica gel. ^1^H and ^13^C nuclear magnetic resonance (NMR) spectra were recorded on Bruker 400 MHz. ^1^H NMR spectra were reported in parts per million (ppm) referenced to 7.26 ppm of CDCl_3_ or referenced to the centerline of a septet at 2.50 ppm of DMSO-d6. Signal splitting patterns were described as singlet (s), doublet (d), triplet (t), quartet (q), quintet (quint), or multiplet (m), with coupling constants (J) in hertz. High-resolution mass spectra (HRMS) were performed on an electron spray injection (ESI) TOF mass spectrometer. The HPLC spectrometry analysis of the final products was processed on a Shimadzu CMB-40 system using a Shimadzu Nexcol C18 column (5 cm × 3.0 mm, 5 μm) for chromatographic separation. Shimadzu SPD-40 LC/MS with multimode electrospray ionization plus atmospheric pressure chemical ionization was used for detection. Method: The mobile phases were 0.1% formic acid in purified water (A) and 0.1% formic acid in MeCN (B). The gradient was increased from 5% to 100% at 10 min, then held at isocratic 100% B for 5 min, and then immediately stepped back down to 5% for 5 min re-equilibration. The flow rate was set at 1.0 mL/min. The column temperature was set at 30 °C. The purities of all of the final compounds were determined to be over 95% by LC–MS. Please refer to SI text Section 6 for more details of chemical synthesis.

### 4.6 Western Blot

PC3 cells were cultured in RPMI-1640 medium (Corning) supplemented with 10% fetal bovine serum and 1% penicillin–streptomycin at 37 °C in a humidified 5% CO_2_ incubator. Western blotting was performed as previously described.^31,128^ Cells were lysed in RIPA buffer, and protein concentrations were determined using the BCA assay. Equal amounts of protein were separated by SDS-PAGE, transferred to PVDF membranes, and probed with antibodies against RIPK1 and β-Actin. Bound antibodies were visualized using the ECL assay (Bio-Rad), and images were captured using the ChemiDoc MP imaging system (Bio-Rad). Antibodies were purchased from Cell Signaling Technology, including Anti-RIPK1 (CS#3493), Anti-RIPK3 (CS#10188), Anti-Phospho-MLKL (CS#91689), Anti-β-Actin (CS#3700), and HRP-conjugated antirabbit IgG (CS#7074).

## Supplementary Material



## Data Availability

The MD simulation trajectories are available from Zenodo (10.5281/zenodo.18155129).

## Notes

Supporting Information Text: details of all-atom MD simulation; microstate MSM construction and validation; IGME model construction and validation; molecular property analysis; details of DiffDock, binding free energy calculations, and Boltz-2 modeling; chemical synthesis; Western blot; Supplementary Figures: additional results and figures that complement the main text; Analytical Characterization Data: NMR, HPLC, and HRMS spectra (PDF)
