# Supplementary material for: Metastable Protein–Protein Interactions as a Design Principle for PROTACs: Insights from the RIPK1–VHL System
Source: JACS Au. 2026 May 7;6(5):2935–48. doi: 10.1021/jacsau.6c00260 (PMC13213396; doi:10.1021/jacsau.6c00260)
Supplement: Supplementary file 1 [file au6c00260_si_001.pdf]

Supporting Information for the manuscript:

Metastable Protein–Protein Interactions as a Design Principle for PROTACs: Insights from the RIPK1–VHL System

Yue Wu[1,†], Zhen Zhang[2,†], Nina J. Hawkins[2,†], Weiping Tang[2,*], Xuhui Huang[1,3,*]

[1]Department of Chemistry, Theoretical Chemistry Institute, University of Wisconsin-Madison, Madison, WI, 53706, USA

[2]Lachman Institute for Pharmaceutical Development, School of Pharmacy, University of Wisconsin-Madison, Madison, WI, 53705, USA

[3]Data Science Institute, University of Wisconsin-Madison, Madison, WI, 53706, USA

[*]To whom correspondence should be addressed.

E-mail: weiping.tang@wisc.edu or xhuang@chem.wisc.edu

†These authors contributed equally.

Table of Contents

Supplementary Information Text …… 1

All-atom Molecular Dynamics (MD) Simulation …… 1

Microstate MSM Construction and Validation …… 2

IGME Model Construction and Validation …… 4

Molecular Property Analysis …… 5

DiffDock, Binding Free Energy Calculations, and Boltz-2 Modeling ⋯ 6

Chemical Synthesis …… 7

Western Blot …… 9

Supplementary Figures …… 11

Analytical Characterization Data …… 30

NMR Data …… 30

NMR Spectrum …… 32

HPLC Data …… 36

HRMS Spectrum …… 40

Supplementary Information Text

1. All-atom Molecular Dynamics (MD) Simulations

1.1 Structure Preparation and Molecular Docking

The conformation of VHL was taken from PDB 5NVW[1], with its bound ligand manually modified to Me-VH032. The conformation of RIPK1 was obtained from PDB 4NEU[2], and the missing loop regions were modelled using SWISS-MODEL[3]. Protonation states of all titratable residues in both VHL and RIPK1 were evaluated using H++[4] and PROPKA[5]. RIPK1 residues H136, H151, and H173 were assigned as HIP (doubly protonated) in the simulations. Since no co-crystal structure of GSK'074 bound to RIPK1 is available, we modelled the RIPK1–GSK'074 binary complex through molecular docking. Docking was performed using AutoDock Vina[6] via the DockingPie[7] PyMOL plugin. Protein–protein docking was then performed using HDOCK[8], applying a 30 Å distance restraint between the two ligands. To remove redundant encounter poses, we computed the pairwise Cα RMSD of VHL after aligning each complex on RIPK1. For any pair of structures with an RMSD < 10 Å, one representative was randomly retained while the other was discarded.

1.2 Force Field Preparation and MD Simulation Setup

In the MD simulations, the Amber14SB force field[9] was used for proteins, and water molecules were modeled using the TIP3P force field[10]. Parameters for small molecules were derived from the General Amber Force Field2 (GAFF2[11]). Ligand geometry optimization and electrostatic potential (ESP) calculations were performed using Gaussian 16[12]. Geometry optimization was carried out at the B3LYP/6-311G(d,p) level with Grimme's D3BJ dispersion correction[13]. ESP calculations employed the Hartree–Fock method with a 6-31G* basis set (HF/6-31G*). Partial atomic charges were generated using the RESP method in AmberTools23[14]. Bonded and Lennard-Jones parameters were taken from GAFF2.

Each complex was solvated in a 14-nm dodecahedral box with TIP3P water, maintaining a minimum 15-Å buffer between the complex and the box edge. Na^+ and Cl^- ions were added to neutralize the system and achieve a physiological salt concentration of 0.15 M. The final system contained 188,802 atoms, with 182 Na^+ ions, 175 Cl^- ions, and 60352 water molecules. Long-range electrostatics were treated using the Particle Mesh Ewald (PME)[15] method, and other nonbonded interactions were calculated with a 12-Å cutoff.

The equilibration MD simulations were conducted using the GROMACS 2022.5[16] package. All systems underwent initial energy minimization using the steepest descent algorithm for 10,000 steps. The LINCS[17] algorithm was then applied to constrain bonds involving hydrogen atoms during subsequent steps. A 1-ns NVT equilibration was performed with position restraints (force constant = 1,000 kJ mol^{-1} nm^{-2}) on all heavy atom, followed by a 1-ns NPT equilibration under the same position restraint settings. A velocity-rescaling thermostat[18] (coupling constant = 0.1 ps) was used to maintain the temperature at 300 K, and the Berendsen barostat[19] was applied during NPT equilibration with a reference pressure of 1 bar and coupling constant of 0.5 ps.

After the initial equilibration, all production simulations were performed in OpenMM 8.0.0[20] under the NVT ensemble, using the Langevin middle integrator[21] with a 2-fs timestep and a friction coefficient of 1.0 ps^{-1}. Following 10-ns local simulations, the final frames of each system were used as the starting structures for large-scale simulations on Folding@Home[22, 23].

To ensure that the resulting trajectories formed a dynamically connected ensemble suitable for kinetic analysis, we applied a geometric filter: the distance between the methyl-group carbon attached to the pyrazole ring of GSK'074 and the left-hand-side (LHS) amide nitrogen in VH-032 was required to remain below 20 Å. Structures violating this threshold were excluded from the analysis due to dynamic disconnection. After filtering, trajectories originating from 31 seeding structures and totaling approximately 420 μs were retained for subsequent analysis (**Figure S1**).

2. Microstate MSM Construction and Validation

2.1 Markov State Model (MSM) Theory

The central concept of Markov State Model (MSM) is to divide the configurational space into a set of discrete states and describe the system's dynamics as Markovian transitions among these states at a lag time τ. The dynamics at this lag time are encoded in the transition probability matrix (TPM), $T(\tau)$, whose element T_{ij} denotes the conditional probability of observing the system in state j after time τ given that it was in state i at the initial time. Under the Markov approximation, these transition probabilities depend solely on the current state and are unaffected by the travelling history. Consequently, once τ is sufficiently large long the dynamics to exhibit Markovian behavior, the evolution of the system can be propagated using the first-order master equation[24, 25]:

$$\boldsymbol{T}(n\tau) = \boldsymbol{T}(\tau)^n \qquad \text{S1}$$

The TPM ($\boldsymbol{T}(\tau)$) at Markovian lag time yields important kinetic information. Its leading eigenvectors describe the population fluxes associated with the slowest dynamical processes, while the corresponding eigenvalues characterize the timescales of these dynamic modes, which can be expressed as implied timescales (ITS) defined as:

$$\text{ITS}_i(\tau) = -\frac{\tau}{ln\,\lambda_i(\tau)}, i = 2,3,\ldots \qquad \text{S2}$$

According to the variational principle[26], the estimated eigenvalues of TPMs as well as the resulting ITS can approach but never exceed the true values under equilibrium condition. Therefore, ITS can serve as a measure of the quality of a kinetic model: the closer the ITS is to the variational bound, the more precise the model. To build and validate the microstate-MSM, we carried out the steps detailed below. MSM analyses were conducted using MSMBuilder 2022[27, 28] together with PyEMMA 2.5.12[29].

2.2 Feature Selection

We used Cα–Cα distances between VHL and RIPK1 as the raw input features. For VHL, we included Cα atoms from all residues, while for RIPK1—which has a longer sequence—we selected every other Cα atom. This resulted in a total of 22,022 raw features. To reduce dimensionality while

preserving the slowest dynamical processes, we applied the spectral Accelerated Sequential Incoherent Selection (spectral oASIS)[30] to select the most informative features. Spectral oASIS uses the Nyström method to reconstruct a large positive semidefinite matrix from a small subset of its columns. At each step, it selects the column whose omission would introduce the largest error in its corresponding diagonal entry. When applied to the time-lagged correlation matrix, this procedure highlights the system's slowest dynamical processes. After tICA, the slowest timescales plateau at 2,000 features as shown in **Figure S2(a)**, so we use this feature set for all subsequent analysis.

2.3 Dimensionality Reduction and Conformational Clustering

After selecting the structural features, we reduced dimensionality to identify a small set of collective variables (CVs) that lower computational cost and improve interpretability. We applied time-lagged Independent Components Analysis (tICA)[31-33] to extract the slowest dynamical modes by maximizing time-lagged autocorrelation. Using the identified CVs, we clustered similar MD conformations into microstates with the K-Means[34] algorithm.

Both the dimensionality-reduction and clustering steps require choosing hyperparameters, including the number of CVs, the tICA lag time, and the number of microstates. We selected these values through cross-validation using the generalized matrix Rayleigh quotient (GMRQ)[35] score to avoid overfitting induced by statistical noise. In GMRQ, the dataset is split into training and testing sets, and the Rayleigh quotient is computed from the eigenvectors and correlation matrices of each split:

$$\text{Train score: } R = \mathrm{Tr}(\boldsymbol{V}^{\mathrm{T}}\boldsymbol{C}(\boldsymbol{V}^{\mathrm{T}}\boldsymbol{S}\boldsymbol{V})^{-1}) \qquad \text{S3}$$

$$\text{Test score: } R_t = \mathrm{Tr}(\boldsymbol{V}^{\mathrm{T}}\boldsymbol{C_t}(\boldsymbol{V}^{\mathrm{T}}\boldsymbol{S_t}\boldsymbol{V})^{-1}) \qquad \text{S4}$$

Here, $\boldsymbol{V}$ is the eigenvector of TPM computed from the training set data. $\boldsymbol{S}$ and $\boldsymbol{C}$ are the diagonal matrix of stationary population and transition count matrix computed from the training set data, and $\boldsymbol{S_t}$ and $\boldsymbol{C_t}$ are the corresponding matrices from the testing set. In practice, the best model is the one with the highest GMRQ score and a small gap between the train and test scores. Based on the GMRQ evaluation (**Figure S2**), we selected a tICA lag time of 30 ns, 4 CVs, and 100 microstates as the optimal parameter combination. For each microstate, the state centers were obtained by selecting the frame closest to its centroid in the 4-dimensional tICA space.

2.4 Validation of Microstate-MSM

To determine the Markovian lag time for the 100-microstate MSM, we examined the implied timescale (computed using Eq. S2) plot and found that the slowest dynamical modes plateau at a lag time of 150 ns as shown in **Figure S3(a)**. We then validated the resulting MSM with the Chapman-Kolmogorov test (Eq. S1), which showed that the predicted residence probabilities for all 100 microstates align well with those obtained directly from the all-atom MD simulations as shown in **Figure S3(b)**.

3. IGME Model Construction and Validation

3.1 Theory of Integrative General Master Equation (IGME)

MSMs can fall short in highly coarse-grained systems, where equilibration within each state introduces memory effects that hinder Markovian behavior and lead to underestimated timescales. The Generalized Master Equation (GME) addresses this limitation by incorporating these memory effects into the dynamics through time-dependent memory kernels. $\boldsymbol{K}(t)$[36-39]:

$$\dot{\boldsymbol{T}}(\tau) = \boldsymbol{T}(\tau)\dot{\boldsymbol{T}}(0) - \int_0^\tau \boldsymbol{T}(\tau - s)\boldsymbol{K}(s)\,\mathrm{d}s \qquad \text{S5}$$

Assuming the memory kernel will decay after τ_K ($\boldsymbol{K}(\tau \geq \tau_K) = \boldsymbol{0}$), IGME obtains an analytic solution for the GME (Eq. S5) when the lag time exceeds the memory decay time τ_K, yielding[37]:

$$\boldsymbol{T}(\tau \geq \tau_K) = \boldsymbol{A}\widehat{\boldsymbol{T}}^\tau \qquad \text{S6}$$

Here, $\widehat{\boldsymbol{T}} = \lim_{\tau\to\infty} \boldsymbol{T}(\tau)^{1/\tau}$ captures the long-term dynamics while $\boldsymbol{A}$ reflects the direct influence of the fast dynamics.

For a lag time τ_M to meet the Markovian condition that links Eq. S6 and Eq S1, it's necessary to satisfy $\ln A \ll \tau_M \ln \hat{T}$[37]. This relationship indicates that traditional MSMs need a τ_M far longer than the memory decay time τ_K used in IGME models. Therefore, IGME models require far less data and can be applied to a wider range of systems.

To assess the accuracy of the IGME models, we used the stationary-population-weighted, time-averaged root mean squared error (RMSE)[37, 39], which quantifies the deviation between the IGME predictions and the reference MD data:

$$\text{RMSE} = \sqrt{\frac{\sum_{n=1}^{L_x}\sum_{i,j=1}^{N}\left[\pi_i \boldsymbol{T}_{ij}^{\text{MD}} - \pi_i \boldsymbol{T}_{ij}^{\text{IGME}}(t)\right]^2 dt}{N^2 L_x}} \qquad \text{S7}$$

Here $\boldsymbol{T}^{\text{IGME}}$ corresponds to the TPMs predicted by IGME and L_x denotes the prediction range of IGME, and π_i is the stationary population in state i.

To perform the analysis of IGME models, we used the python code from Ref[37], which is publicly available at https://github.com/xuhuihuang/IGME.

3.2 Kinetic Lumping

With a validated microstate-MSM model, we further lumped 100 microstates into 6 metastable states, ensuring slow transitions between states and fast relaxation within each state. For this kinetic lumping step, we applied Robust Perron Cluster Analysis (PCCA+), a widely used approach for identifying metastable states[40, 41]. PCCA+ leverages the fact that the leading eigenvectors of the microstate transition probability matrix form an approximately simplex-like structure. Using this structure, it assigns each microstate membership values for the candidate metastable states and then optimizes these values for maximal metastability[42]. Each microstate is finally placed into the metastable state for which it shows the highest membership values. Here we chose six metastable states because this number yielded relatively low prediction RMSE (**Eq.** S7) in the IGME models as we can see in **Figure S4(a)**.

3.3 Construction and Validation of IGME Models

In the construction of IGME models, we estimated the two key parameters $\boldsymbol{A}$ and $\widehat{\boldsymbol{T}}$, as shown in Eq. S6, using a least-square fitting (LSF) method[37, 43]. The LSF was applied on a selected subset of the input TPMs: $[\boldsymbol{T}(\tau_K^{\text{trial}}), \boldsymbol{T}(\tau_K^{\text{trial}} + \Delta t), \ldots, \boldsymbol{T}(\tau_K^{\text{trial}} + L)]$, where τ_K^{trial} serves as an initial guess for the memory decay time τ_K, and L defines the span of data included in the fit procedure. As shown in **Figure S4(b)**, we then performed a systematic search over the hyperparameters (τ_K^{trial} and L) to obtain the optimal IGME models that minimized the prediction RMSE (Eq. S7). For this system, we fit IGME models using TPM with lag times shorter than 100 ns, *i.e.*, $\tau_K^{\text{trial}} + L < 100\,\text{ns}$. Compared with conventional MSM, IGME models exhibited consistently improved performance. IGME models with different combinations of hyperparameters maintained stably low RMSE values. Besides, they approached the variational bound more closely and produced implied timescales comparable to or even exceeding those of 300-ns MSMs (**Figure S4(c)**). In addition, IGME models successfully passed the Chapman–Kolmogorov test, whereas the 150-ns MSM did not (**Figure S4(d)**). The top 5% models with the lowest RMSE values were used to estimated $\widehat{\boldsymbol{T}}$ and characterize the dynamics and thermodynamics of the 6-state model.

4. Molecular Property Analysis

SASA values were calculated using MDTraj[44] v1.9.9. Unless otherwise noted, all other molecular properties were computed using MDAnalysis[45] v2.2.0. The structures were visualized using PyMOL (Schrödinger)[46].

4.1 Interface RMSD

Each metastable state contains multiple microstate centers, and each center was used as a reference structure. For a given reference, interface residues were defined as those whose minimum heavy-atom distance to the partner protein was under 10 Å. All frames assigned to the metastable state were then aligned to the reference using the heavy atoms of these interface residues, and the RMSD of the interface heavy atoms was computed for each frame and averaged. Repeating this procedure for each microstate center produced one interface RMSD value per center. The metastable state-level interface RMSD was reported as the mean and standard deviation of these values.

4.2 Buried Surface Area (BSA)

The buried surface area (BSA) was calculated as the sum of the solvent-accessible surface areas (SASA) of the two individual proteins minus the SASA of the protein complex. Hydrogen atoms were included in protein SASA calculations. For each metastable state, BSA was computed for every frame, and the metastable state-level value was reported as the mean and standard deviation across those frames.

4.3 Heavy Atom SASA for RIPK1 Ligand and E3 Ligand

When evaluating linkage potential, we computed SASA for all heavy atoms in the ligands as shown in **Figure 3(a-b)**. Hydrogen atoms in ligands were excluded from this analysis to avoid artificially inflating accessibility values. In the subsequent ranking of per-atom SASA values (**Figure S11**),

we noted that Site 1 contains three symmetry-equivalent atoms and that Site 3 and o-Ph-C each contain two equivalent atoms. To avoid redundancy, these equivalent atoms were grouped, and for each frame we retained the largest SASA value within each equivalence class. The mean and standard deviation were then computed over these representative values.

4.4 Linkage Atoms Distances

Because the pyrazole ring in GSK'074 can rotate, the methyl substituent at Site C can adopt both *cis* and *trans* orientations. To ensure that both possibilities were captured in our geometric analysis, we introduced a pseudo-atom representing Site C in each conformation. During distance-based linker assessment (Left panel of **Figure 3(d-g)**), distances from both the *cis* and *trans* positions were computed and considered in parallel. For linkage sites in VHL containing symmetry-equivalent atoms, the atom with the highest solvent exposure in each frame was selected for distance calculations.

4.5 Linker Length

Linker lengths were obtained as the mean values from 1,000 conformations randomly generated using RDKit[47] v2025.05.3.

5. DiffDock, Binding Free Energy Calculations, and Boltz-2 Modeling

Each metastable state consists of numerous microstates, some of which may represent low-population or transition-state-like conformations connecting to other metastable states. To focus on the most representative conformations, we defined a "core region" within each metastable state, including only those microstates with stationary populations exceeding 1%. As illustrated in **Figure S12**, these core regions correspond to structural ensembles near the center of each state's free energy basin.

From each core region, we randomly selected 200 MD snapshots. All ligands were removed from these snapshots, and the resulting VHL–RIPK1 binary complexes were collected to form a receptor ensemble for docking. For each PROTAC molecule compatible with the geometric constraints of a given metastable state (including linker length and linkage sites), we performed DiffDock[48] docking against the 200 receptor structures associated with that metastable state. DiffDock was run using its default sampling parameters, producing one docked pose per receptor structure. All poses were ranked by ligand RMSD, calculated without superposing ligand coordinates in order to avoid artifacts introduced by forced alignment. For each PROTAC–state pair, the five lowest-RMSD poses were selected for downstream binding free energy evaluation. Docked structures exhibiting severe steric clashes that resulted in positive binding free energies were excluded.

Each selected pose was subjected to a 10 ns all-atom MD simulation. The equilibration process followed the same protocol described in Section 1.2 of the SI. Production runs were performed in the NPT ensemble using the velocity-rescaling thermostat[18] (τ = 0.1 ps, T = 300 K) and the Parrinello–Rahman barostat[49] (τ = 2 ps, P = 1 bar) using the GROMACS 2022.5[16] package. The final 5 ns of each trajectory were extracted for computing the binding free energy between the PROTAC and the VHL–RIPK1 complex.

To estimate the thermodynamic stability of the ternary complex, we decomposed its formation into two sequential steps: first, the formation of the PPI between the E3 ligase (VHL) and the target protein (RIPK1), followed by the binding of the PROTAC molecule:

$$E + T \xrightleftharpoons{\Delta G_{E+T}} E \cdot T \qquad E \cdot T + P \xrightleftharpoons{\Delta G_{ET+P}} E \cdot P \cdot T \tag{S8}$$

Here E, T, P denote the E3 ligase, the target protein, and the PROTAC molecule, respectively. For a given metastable PPI, the free energy of protein–protein association: ΔG_{E+T} is fixed. Differences in ternary complex stability among PROTAC molecules therefore arise from the second step, ΔG_{ET+P}, corresponding to PROTAC binding to the preformed PPI. In this work, we estimate ΔG_{ET+P} using the gmx_MMPBSA tool[50] based on the Molecular mechanics/Poisson–Boltzmann Surface Area (MM/PBSA) method. The dielectric interface was defined using a level set function[51], and the non-polar solvation free energy[52] was treated as a single SASA-dependent term. The internal and external dielectric constants were set to 1.0 and 80.0, respectively, to mimic the protein interior and aqueous solvent. A halogen-optimized atomic radii set (mbondi_pb2)[53] was applied, and the ionic strength was set to 0.150 M to reflect physiological salt conditions.

To estimate the ensemble-averaged binding free energies and their uncertainties for each PROTAC–state pair, we performed bootstrap resampling of the five trajectories. For each of 10 bootstrap iterations, the 5 trajectories were resampled with replacement, and the binding free energy of that bootstrap set was computed as the average of the selected samples. The final binding free energy corresponds to the average across all bootstrap iterations, and the standard deviation across iterations was taken as the statistical error. If a PROTAC molecule was compatible with the distance range of multiple metastable PPIs, we selected the lowest predicted binding free energy as the representative value for comparison across compounds. The full state-resolved binding free energy data are provided in **Figure S13** (Site 2 linkers) and **Figure S15** (Site 3 linkers). To enable comparison, the fluorocyclopropyl group in Site 3 was not modelled in simulations and binding free energy calculations.

For compounds **269-2** and **269-10**, Boltz-2[54] co-folding was also used for structural modeling. The protein sequences used for co-folding were identical to those employed in the MD simulations. For each compound, 5 ternary complex structures were generated using the default Boltz-2 settings. Following structure generation, the proteins were protonated using the GROMACS pdb2gmx program[16], and the BSA was calculated with hydrogens included.

6. Chemical Synthesis

All reactions were conducted under a positive pressure of dry argon in glassware that had been oven-dried prior to use. Anhydrous solutions of reaction mixtures were transferred via an oven-dried syringe or cannula. All solvents were dried prior to use unless noted. Thin-layer chromatography (TLC) was performed using precoated silica gel plates. Flash column chromatography was performed with silica gel. ^{1}H and ^{13}C nuclear magnetic resonance (NMR) spectra were recorded on Bruker 400 MHz. ^{1}H NMR spectra were reported in parts per million (ppm) referenced to 7.26 ppm of $CDCl_3$ or referenced to the centerline of a septet at 2.50 ppm of

DMSO-d6. Signal splitting patterns were described as singlet (s), doublet (d), triplet (t), quartet (q), quintet (quint), or multiplet (m), with coupling constants (J) in hertz. High-resolution mass spectra (HRMS) were performed on an electron spray injection (ESI) TOF mass spectrometer.

The HPLC spectrometry analysis of the final products was processed on a Shimadzu CMB-40 system using a Shimadzu Nexcol C18 column (5 cm × 3.0 mm, 5 μm) for chromatographic separation. Shimadzu SPD-40 LC/MS with multimode electrospray ionization plus atmospheric pressure chemical ionization was used for detection. Method: The mobile phases were 0.1% formic acid in purified water (A) and 0.1% formic acid in MeCN (B). The gradient was increased from 5% to 100% at 10 min, then held at isocratic 100% B for 5 min, and then immediately stepped back down to 5% for 5 min re-equilibration. The flow rate was set at 1.0 mL/min. The column temperature was set at 30 °C. The purities of all of the final compounds were determined to be over 95% by LC−MS.

The synthesis of RIPK1 PROTACs is summarized in Scheme 1. The mono Boc-protected diamine and RIPK1 binder were coupled to form the amide intermediate. After Boc-deprotection, the resulting free amine was reacted with VHL ligand to yield the final product.

General Procedure a: (Condensation of mono Boc-protected diamine with RIPK1 binder): To mono Boc-protected diamine (1.0 eq) and reported RIPK1 binder (1.05 eq) in DMF, were added HATU (1.2 eq) and DIPEA (3.0 eq) successively. The solution was stirred at room temperature for 2h then added with EtOAc and water. The water phase was extracted with EtOAc (3×) and the combined organic phase was washed with water, brine, dried over sodium sulfate, filtered and condensed to afford a residue which was purified by flash column chromatography on silica to afford the desired compound.

Procedure b: (Boc deprotection and condensation with tert-Butyl N-(2-bromoethyl) carbamate): To a stirring solution of Boc-protected intermediate (1 eq) in DCM was added TFA (5.0 eq). After stirring at room temperature for 1 h, the mixture was condensed under reduced pressure to afford TFA salt quantitatively. The solid was dissolved in DMF, K_2CO_3 (2.5 eq) was added to neutralize the TFA, followed by the addition of tert-Butyl N-(2-bromoethyl) carbamate (1.05 eq). The solution was stirred at 90 °C for 4h then added with EtOAc and water. The water phase was extracted with EtOAc (3×) and the combined organic phase was washed with water, brine, dried over sodium sulfate, filtered and condensed to afford a residue which was purified by flash column chromatography on silica to afford the desired compound.

Procedure c: (Boc deprotection and condensation with RIPK1 binder): To a stirring solution of Boc-protected intermediate (1 eq) in DCM was added TFA (5.0 eq). After stirring at room temperature for 1 h, the mixture was condensed under reduced pressure to afford TFA salt quantitatively. The solid was dissolved in THF (6 mL) and acetonitrile (1.5 mL), followed by the addition of 1-Boc-piperidine-4-carboxaldehyde (1.05 eq). After stirring for 5 h, sodium triacetoxyborohydride (1.2 eq) was added and the reaction was allowed to stir overnight. The next morning, the solvents were removed by rotary evaporation and the residue was dissolved in ethyl acetate, washed twice with satd aq. $NaHCO_3$, followed by water, then dried over $MgSO_4$, filtered and concentrated in vacuo. The residue was then chromatographed over silica gel to afford the

desired compound.

General Procedure d: (Boc deprotection and condensation with VHL ligand): To a stirring solution of Boc-protected intermediate (1 eq) in DCM was added TFA (5.0 eq). After stirring at room temperature for 1 h, the mixture was condensed under reduced pressure to afford TFA salt quantitatively. The solid was dissolved in DMF, DIPEA (1.5 eq) was added to neutralize the TFA, followed by the addition of a mixed solution of VHL ligand (1 eq), HATU (1.2 eq) and DIPEA (2 eq) in DMF dropwise at 0°C. The solution was stirred at room temperature for 2h then added with EtOAc and water. The water phase was extracted with EtOAc (3×) and the combined organic phase was washed with water, brine, dried over sodium sulfate, filtered and condensed to afford a residue which was purified by reverse-ISCO to yield final compound.

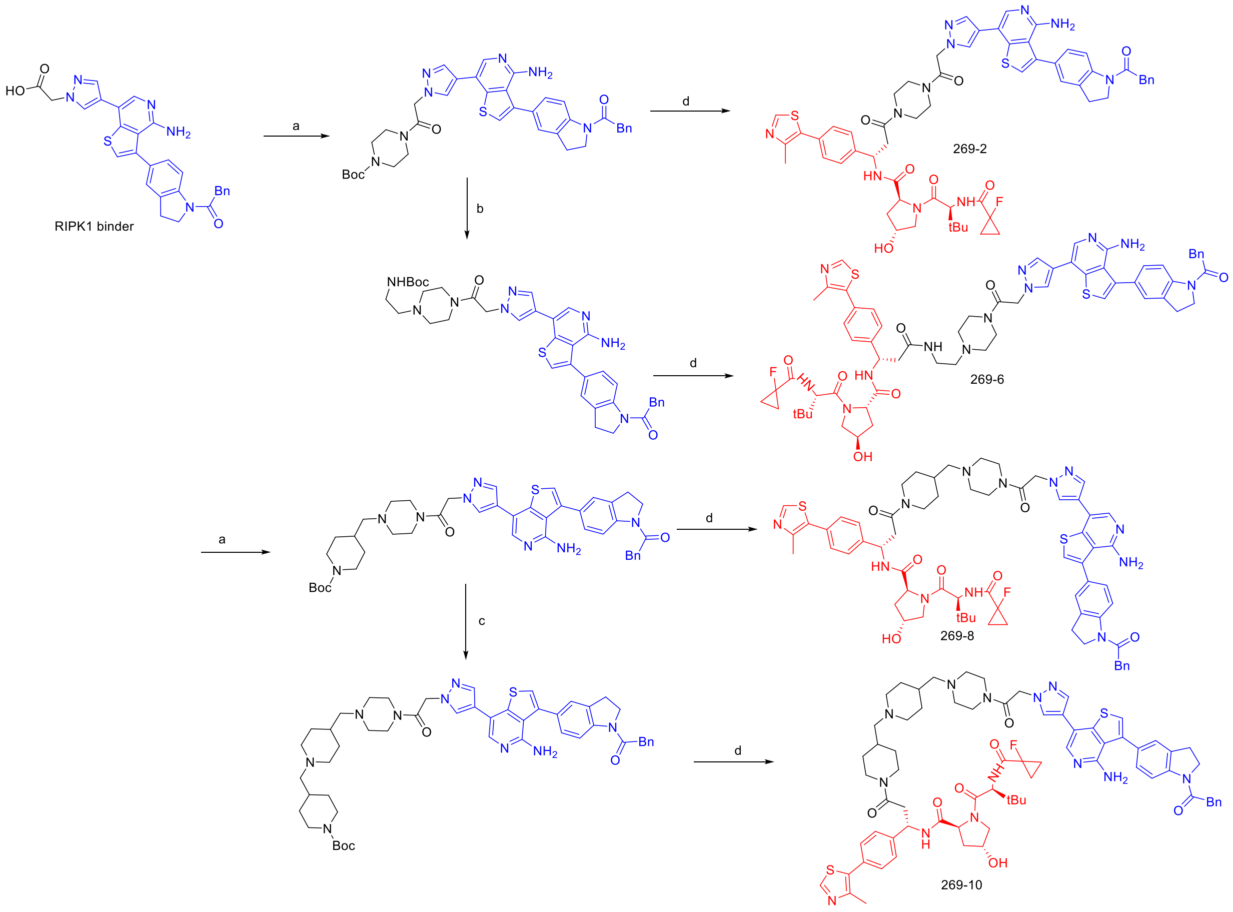

Scheme 1. Synthesis of compounds 269 Series**.** Reagent and conditions: (a) linker, HATU, DIPEA, DMF, rt. (b) TFA, DCM, rt; then linker, K_2CO_3, DMF, 90°C. (c) TFA, DCM, rt; then linker, THF/MeCN (V/V = 4:1), $Na[(CH_3COO)_3BH]$, rt. (d) TFA, DCM, rt; then VHL ligand, HATU, DIPEA, DMF, rt.

7. Western Blot

Cell Culture. PC3 cells were cultured in RPMI-1640 medium (Corning), supplemented with 10% fetal bovine serum (FBS) and 1% Penicillin-Streptomycin. All cells were incubated at 37°C in a 5% CO2 incubator.

Western Blot Assay. Western blotting was performed as previously described[55, 56]. Cells were lysed in RIPA buffer, and protein concentrations were determined using the BCA assay. Equal amounts of protein were separated by SDS-PAGE, transferred to PVDF membranes, and probed with antibodies against RIPK1 and β-Actin. Bound antibodies were visualized using the ECL assay (Bio-Rad), and images were captured using the ChemiDoc MP imaging system (Bio-Rad). Antibodies were purchased from Cell Signaling Technology, including Anti-RIPK1 (CS#3493), Anti-RIPK3 (CS#10188), Anti-Phospho-MLKL (CD#91689), Anti-β-Actin (CS#3700), and HRP-conjugated anti-rabbit IgG (CS#7074).

Supplementary Figures

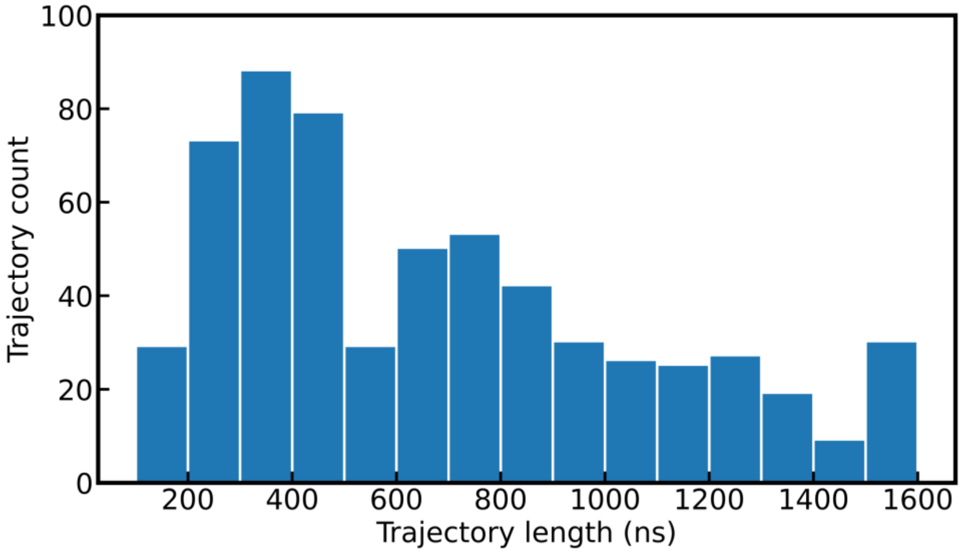

Figure S1. Distribution of trajectory lengths generated on the Folding@Home platform.

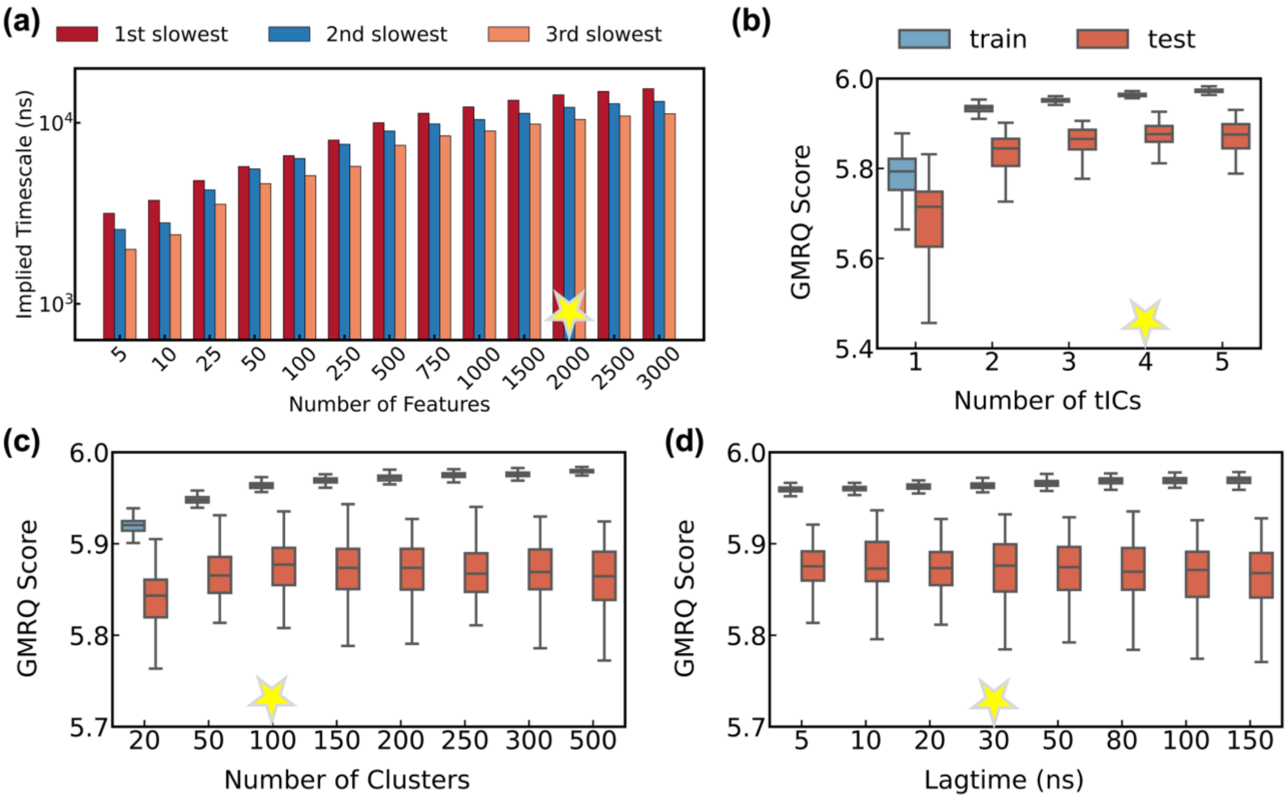

Figure S2. Optimization of microstate-MSM related parameters. (a) The top three slowest implied timescales computed using time-lagged correlation matrices constructed with different numbers of features selected by the spectral-oASIS algorithm. A selection of 2,000 features was chosen because the implied timescales plateau at this number. **(b)** Cross-validation of GMRQ scores for selecting the optimal number of tICs. **(c)** Cross-validation of GMRQ scores for determining the optimal number of clusters. **(d)** Cross-validation of GMRQ scores for selecting the tICA lag time. A combination of 4 tICs, 100 clusters, and 30-ns tICA lagtime was selected. When screening one parameter, the other two were fixed at their optimal values.

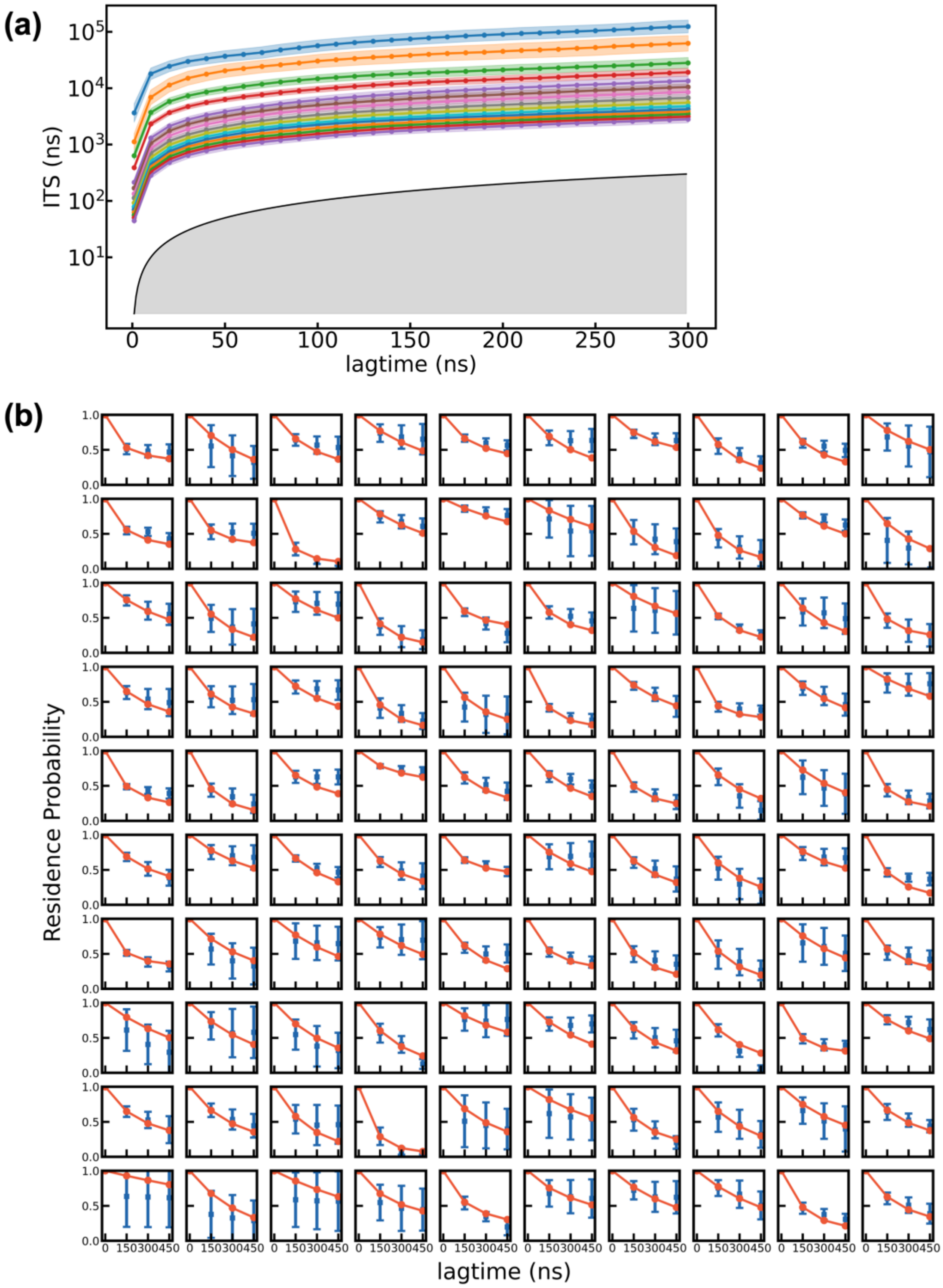

Figure S3. Validation of the Microstate-MSM. (a) Implied timescales (ITS) for the 15 slowest dynamical processes. The ITS curves converge at a lag time of 150 ns, indicating an appropriate Markovian timescale for model construction. **(b)** Chapman–Kolmogorov (CK) test evaluating the residence probabilities of all 100 microstates. The 150-ns MSM passes the CK test with high predictive accuracy, confirming good Markovian behavior and validating the microstate-level MSM. The error bars represent standard deviations estimated from 50 bootstraps of MD data with replacement.

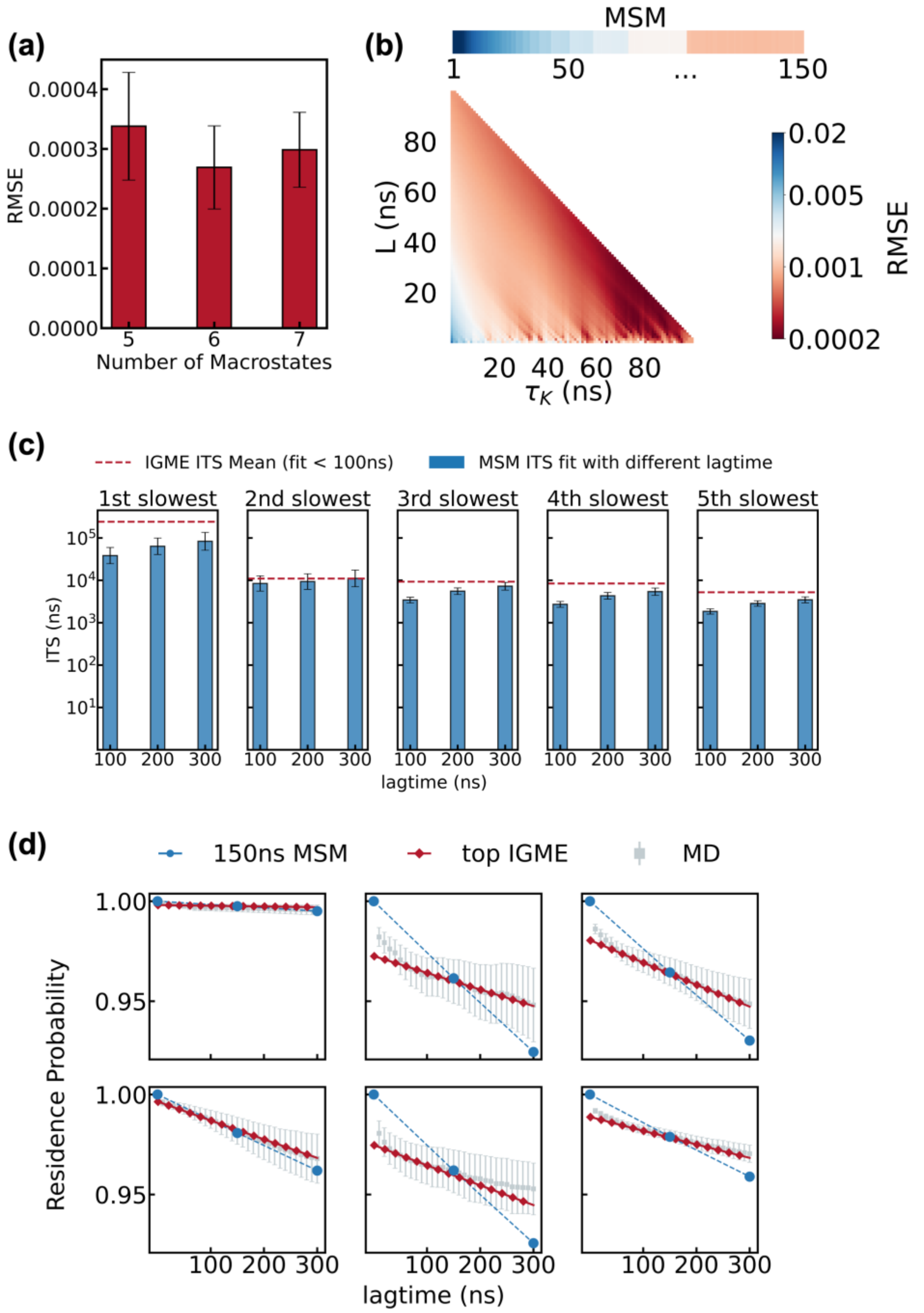

Figure S4. Comparison between MSMs and IGME models. (a) Mean RMSE values for the top 5% IGME models constructed with varying numbers of metastable states, with standard deviations estimated from 50 bootstrap samples of the MD trajectories. The 6-state IGME model shows the lowest prediction error and was therefore selected. **(b)** Parameter scan of IGME models (τ_k and L) for the 6-state system, using lag times shorter than 100 ns for model fitting. Darker red colors indicate smaller prediction errors. For comparison, MSMs trained at different lag times exhibit substantially higher RMSE values. **(c)** Implied timescales (ITS) computed from IGME and MSM models. The red dashed line shows the mean ITS from the top 5% IGME models, while the blue bars represent ITS values from MSMs trained at different lag times. Error bars for MSM ITS values reflect standard deviations from 50 bootstrap samples. **(d)** Chapman–Kolmogorov (CK) tests for the 150-ns MSM and the best IGME model. Error bars represent standard deviations estimated from 50 bootstrap samples of the MD data. The IGME model shows markedly improved CK consistency compared to the MSM.

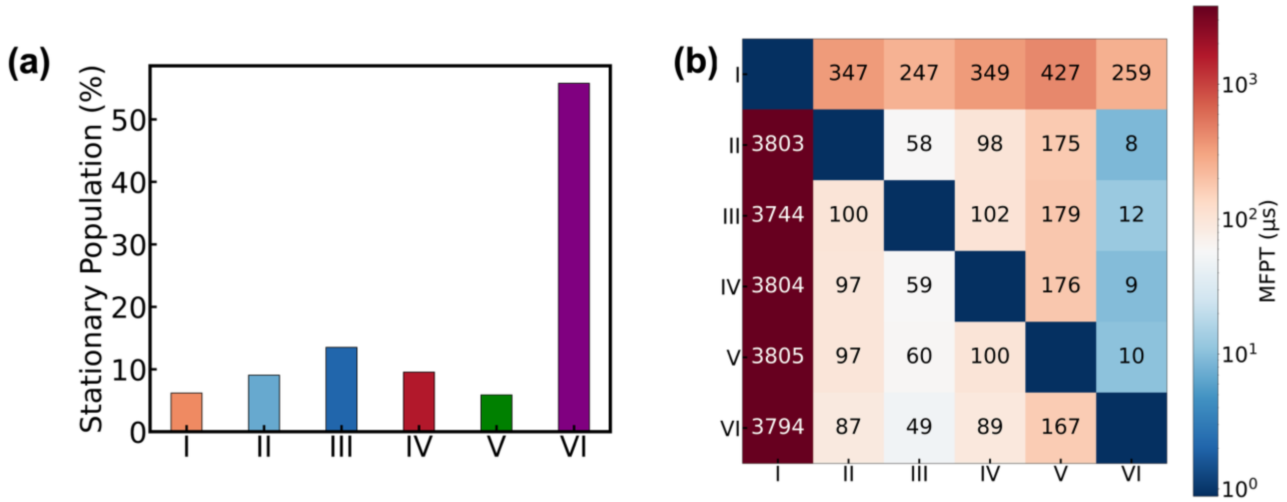

Figure S5. Thermodynamic and Kinetic Properties Identified from Top 5% IGME Models. (a) Average stationary populations of the six metastable states computed from the top 5% IGME models. **(b)** Average mean-first-passage times (MFPTs) between each pair of states. Matrix element (i, j) corresponds to the MFPT for transitions from State i to State j.

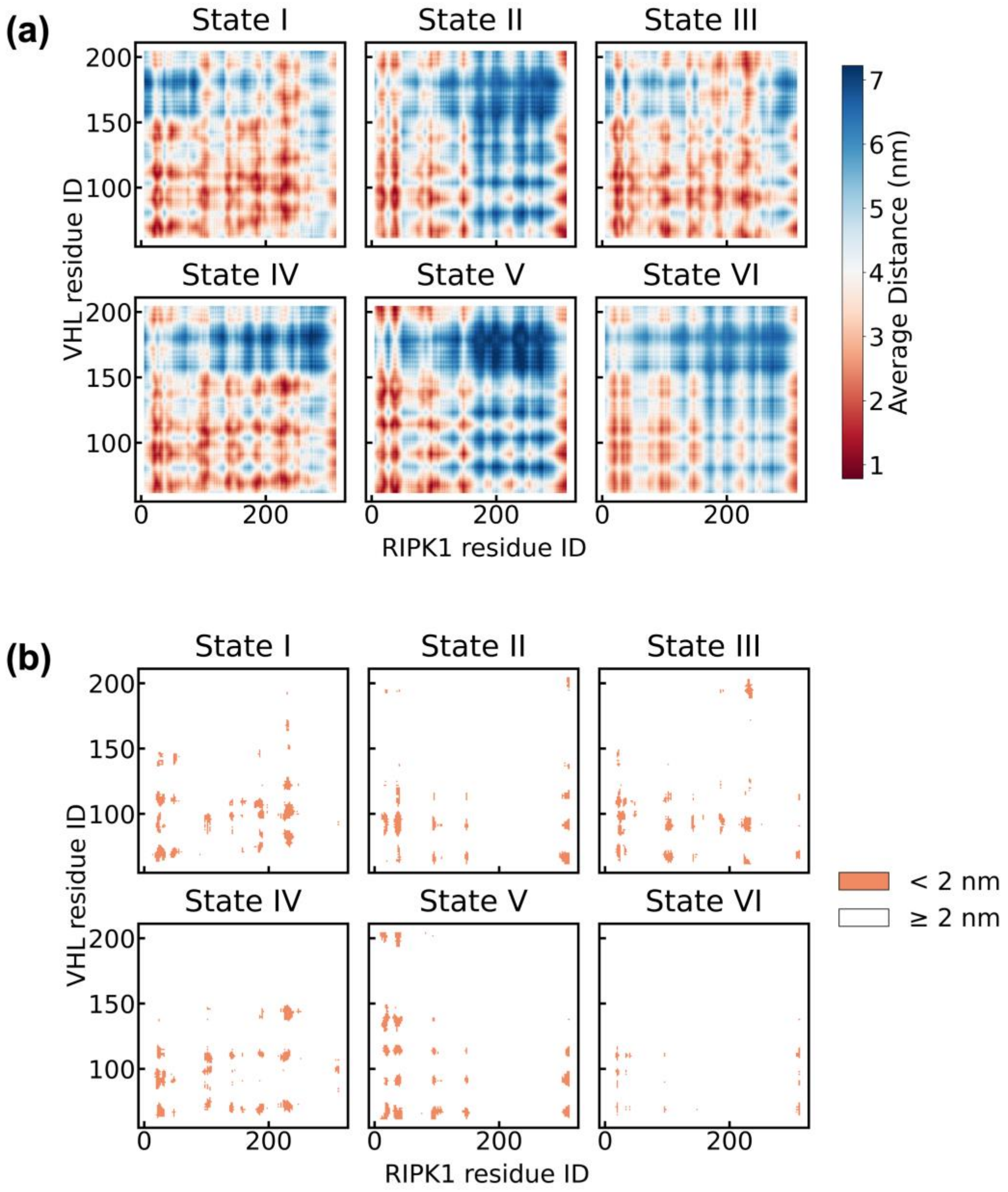

Figure S6. Inter-protein pairwise distance maps for the six metastable states. **(a)** Average inter-protein Cα–Cα distances between RIPK1 and VHL across all frames in each metastable state. Dark red indicates close proximity, whereas dark blue indicates large separation. **(b)** Same data as in **(a)** visualized with a threshold-based color scheme: orange denotes average distances < 2 nm, and white denotes distances > 2 nm.

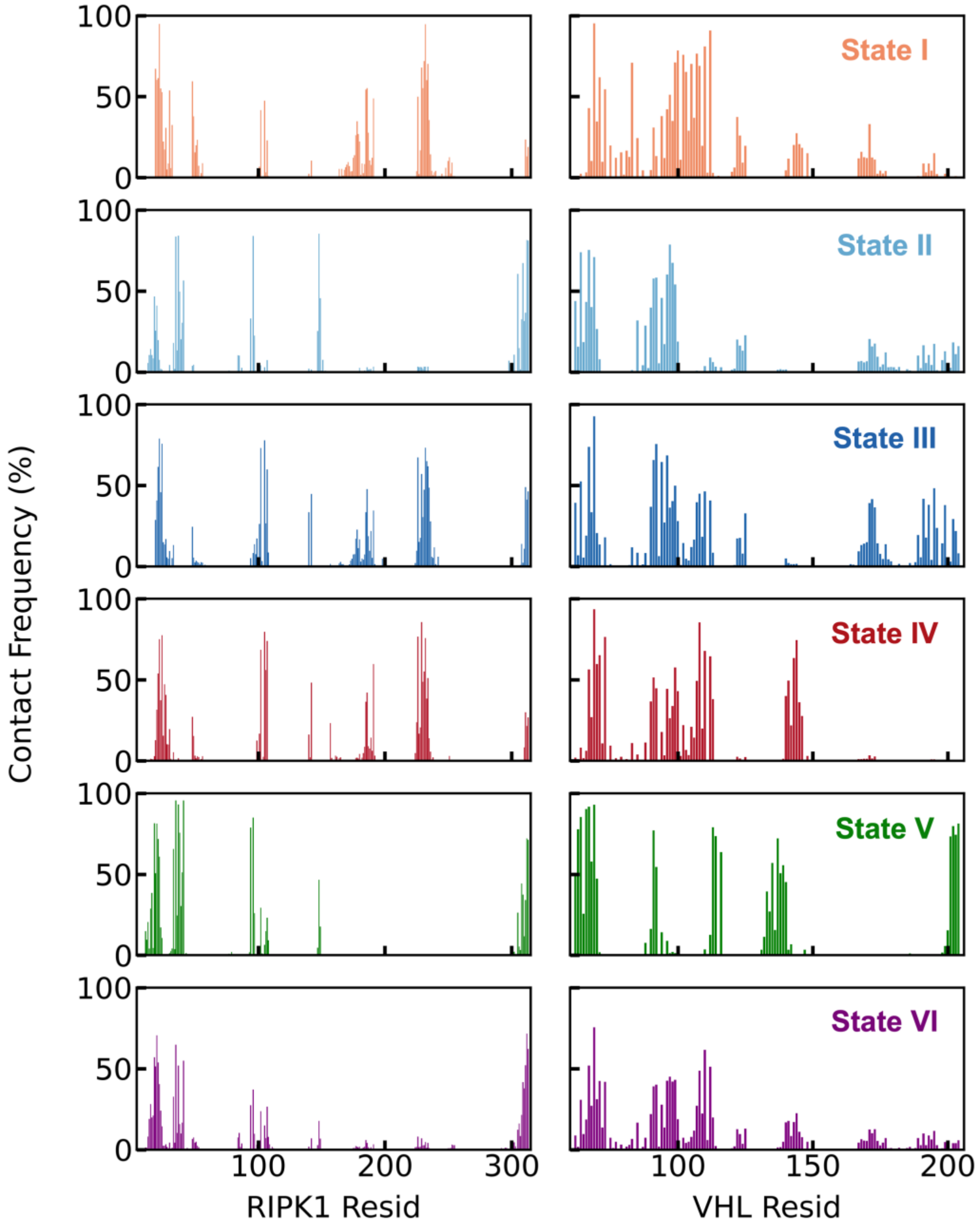

Figure S7. Residue-level contact frequency profiles for each metastable state. A residue is considered in contact if any of its heavy atoms lies within 4.5 Å of any heavy atom of the partner protein. Contact frequency reflects the fraction of frames in which this condition is satisfied among each metastable state.

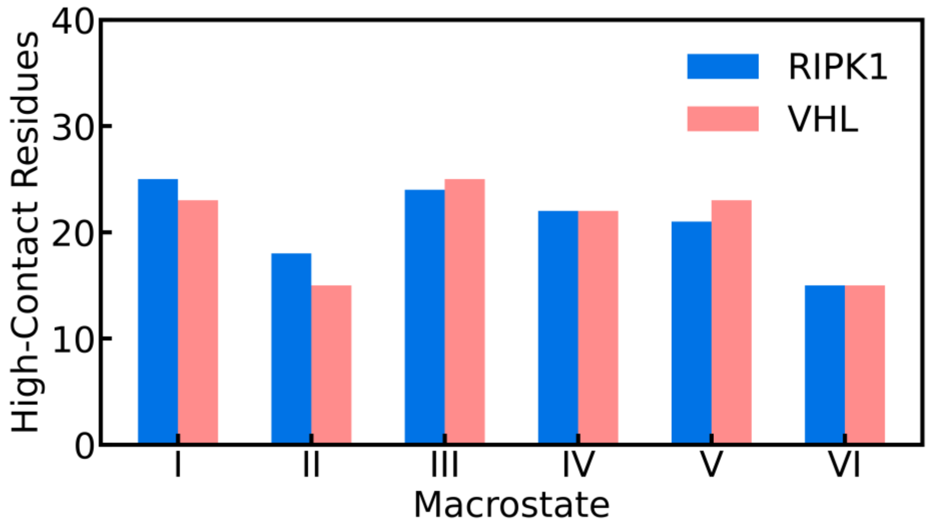

Figure S8. Number of high-contact residues. Residues with contact frequencies greater than 30% are classified as high-contact residues. RIPK1 residues are displayed in marine, whereas VHL residues are displayed in salmon.

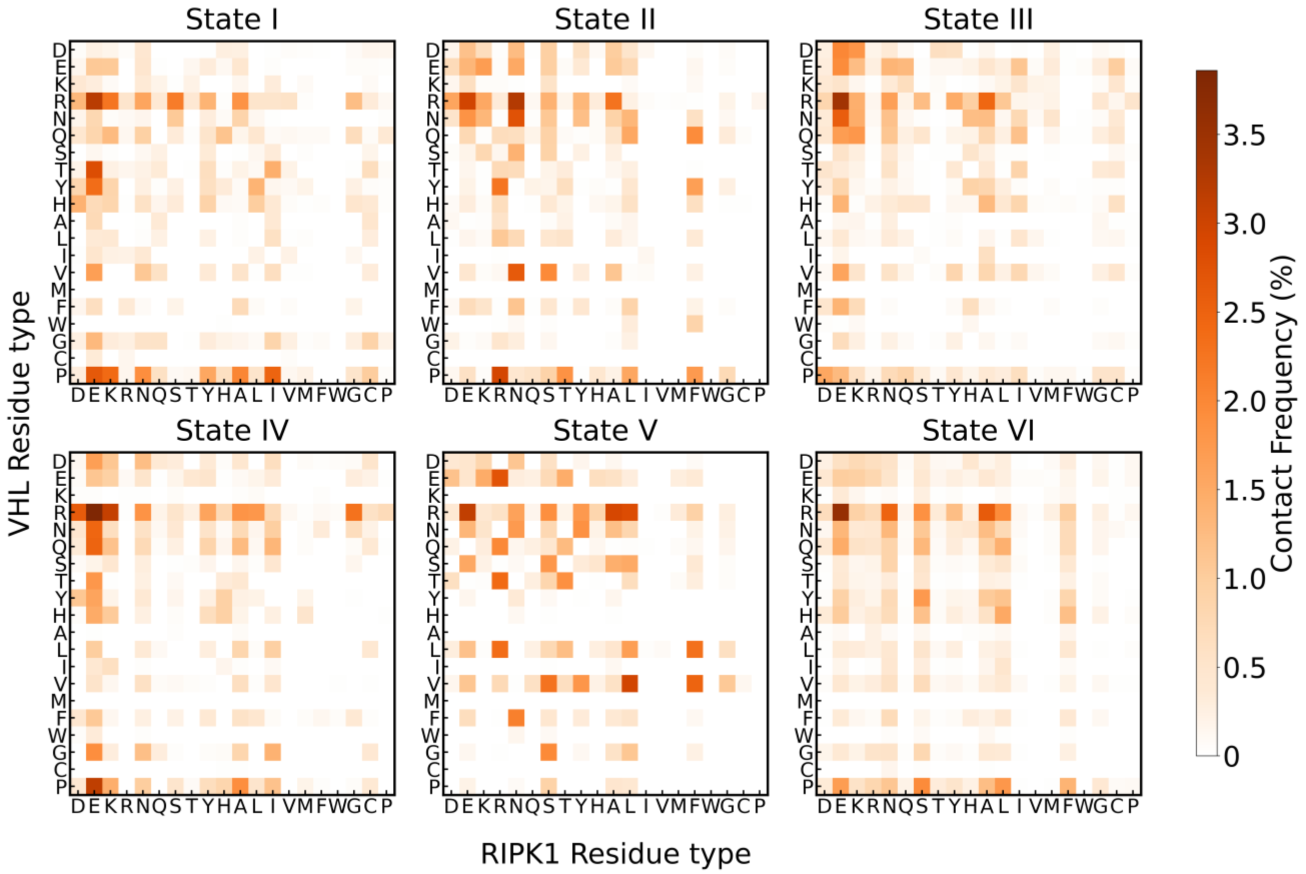

Figure S9. Contact frequency with respect to residue types for each metastable state.

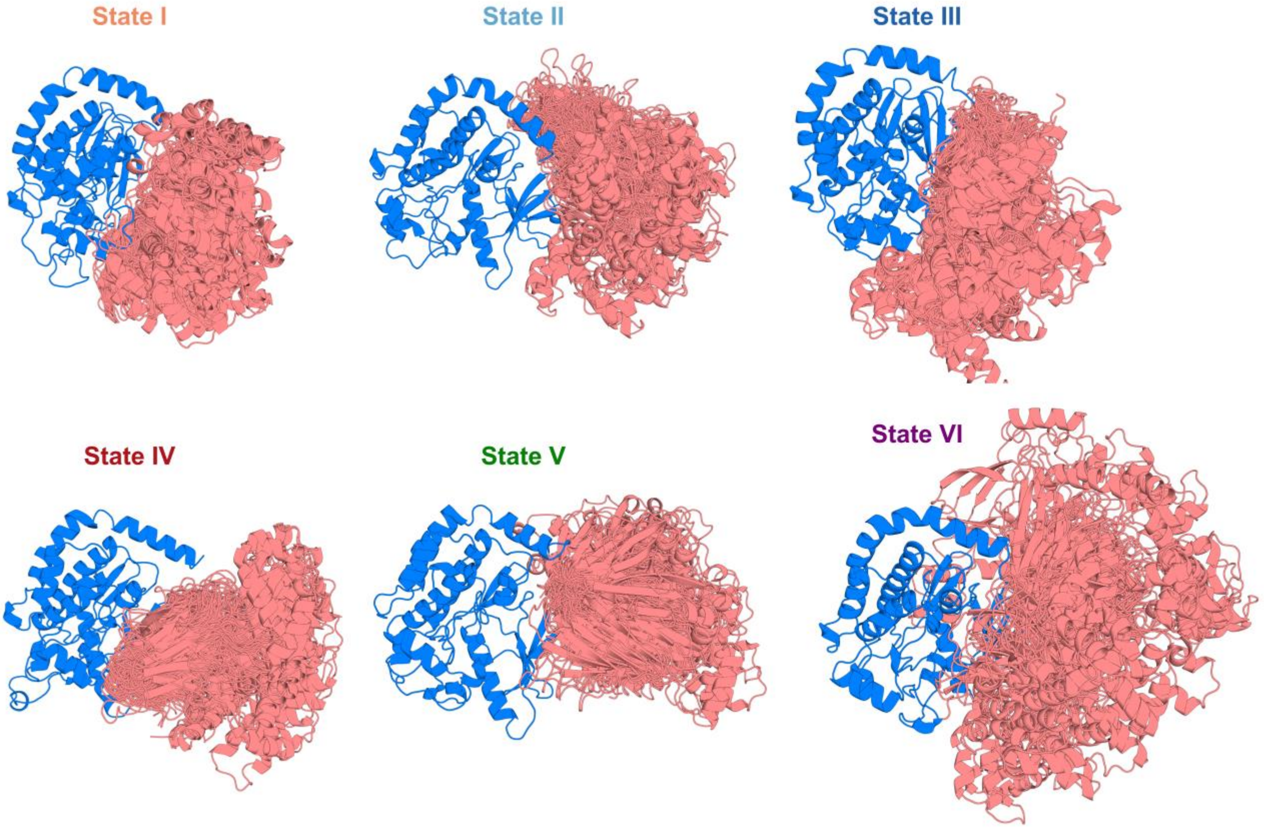

Figure S10. Conformational ensembles of each metastable state. Thirty frames are randomly sampled from each metastable states and aligned on RIPK1 to illustrate the structural heterogeneity. RIPK1 is shown in marine and VHL in salmon.

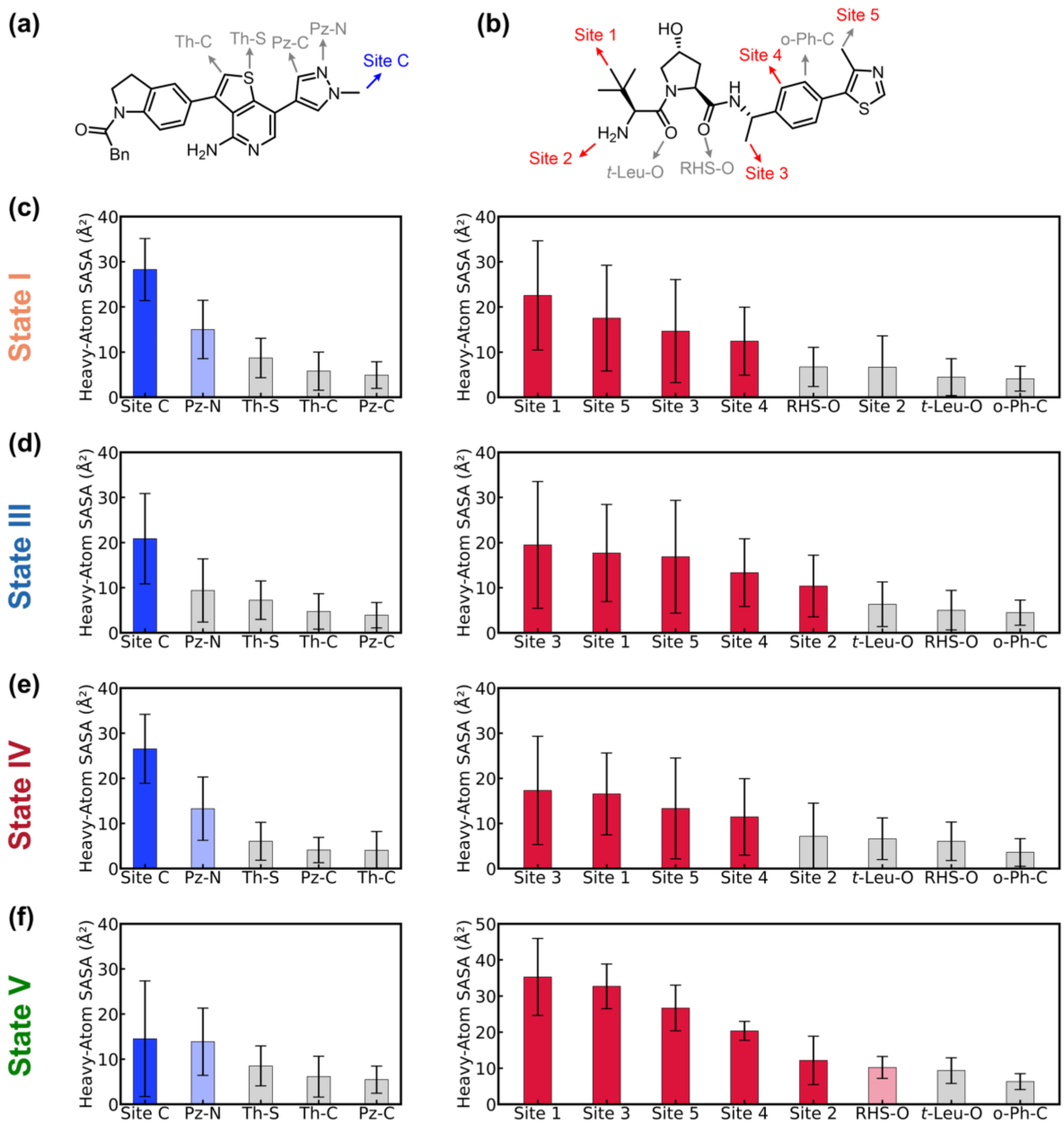

Figure S11. Ranking solvent-accessible heavy atoms on RIPK1 and VHL ligands across shortlisted metastable PPIs. (a) Chemical structure of the RIPK1 ligand, with the only solvent-exposed methyl substituent on the pyrazole ring annotated as Site C. **(b)** Chemical structure of the VHL ligand, highlighting five experimentally validated exit vectors (Sites 1–5). **(c–f)** Ranking heavy atoms in RIPK1 ligand (left panels) and VHL ligand (right panels) in states I, III, IV, and V based on average SASA. For substituents containing symmetry-equivalent atoms, equivalent positions were merged by selecting the maximum SASA per frame prior to averaging. Color scheme: dark blue/red bars indicate atoms that satisfy the 10 Å^2 threshold for solvent exposure and are chemically accessible for covalent linker installation; light blue/light red bars indicate atoms exceeding 10 Å^2 SASA but not chemically suitable for covalent modification; gray bars denote atoms with mean SASA values below 10 Å^2 and therefore considered insufficiently exposed.

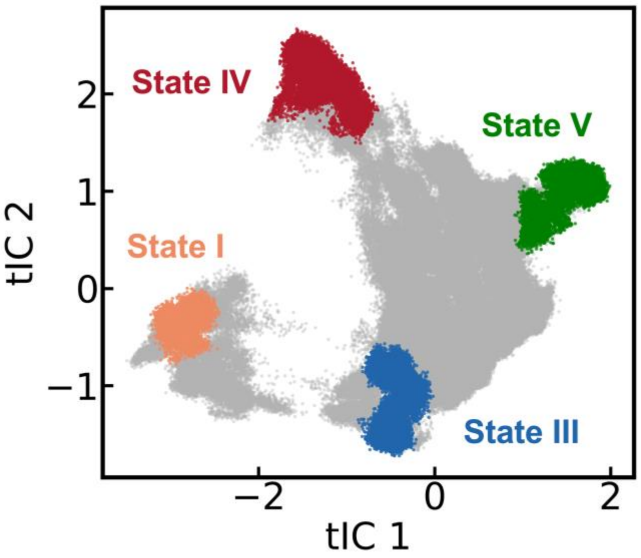

Figure S12. Core-region projection onto the first two tICA components. Microstates belonging to the shortlisted PPIs (States I, III, IV, and V) were classified into core regions by selecting those with stationary populations greater than 1%. These core microstates are highlighted in color (State I in orange, State III in blue, State IV in red, and State V in green), while all remaining frames are shown in grey.

(a)

linker	216-9	216-11	216-13	216-15	225-2	225-3	225-4	225-5	225-6	225-7	229-1	229-2
Length (Å)	5.2	7.7	9.3	10.8	11.7	8.1	12.5	16.4	10.1	10.7	11.7	11.9

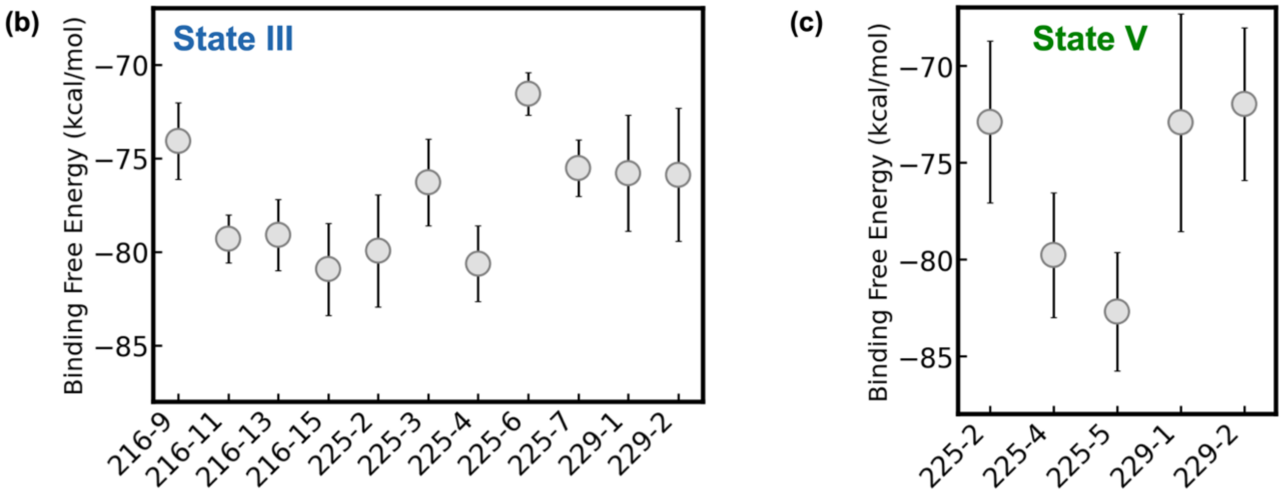

Figure S13. Site 2 linkers evaluated in State III and State V. (a) Length of different Site 2 linkers. **(b)** Predicted binding free energies for Site 2 linkers compatible with State III (linker length < 14 Å). **(c)** Predicted binding free energies for Site 2 linkers compatible with State V (linker length > 11 Å). Mean values and standard deviations are obtained via bootstrapping.

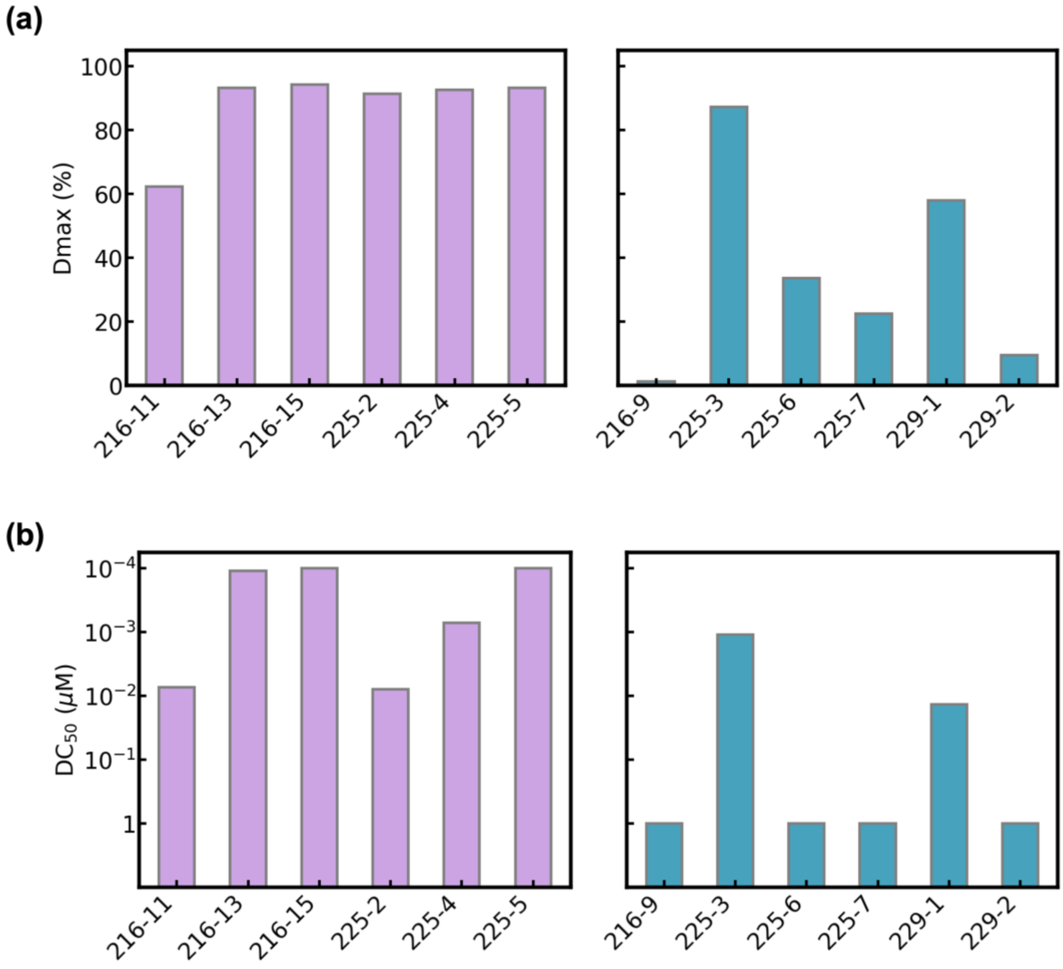

Figure S14. Degradation activities for Site 2 linkers. (a) D_{max} values, representing the maximum extent of RIPK1 protein reduction achieved by each compound. **(b)** DC_{50}, denoting the concentration required to reduce RIPK1 protein levels by 50%. Compounds classified in the top 50% based on predicted binding free energy are shown in violet (left panel), whereas those in the bottom 50% are shown in teal (right panel).

(a)

linker	269-2	269-6	269-8	269-10
Length (Å)	7.2	10.0	11.6	16.0

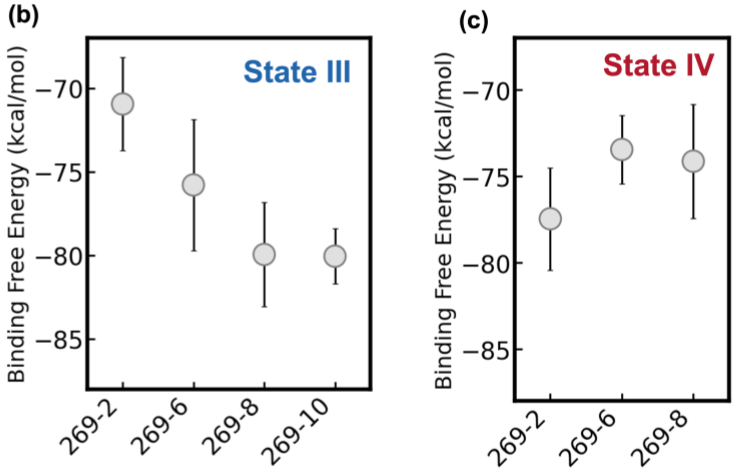

Figure S15. Site 3 linkers evaluated in State III and State IV. (a) Length of different Site 3 linkers. **(b)** Predicted binding free energies for Site 3 linkers compatible with State III (6 Å < linker length < 17 Å). **(c)** Predicted binding free energies for Site 3 linkers compatible with State IV (linker length < 14 Å). Mean values and standard deviations are obtained via bootstrapping.

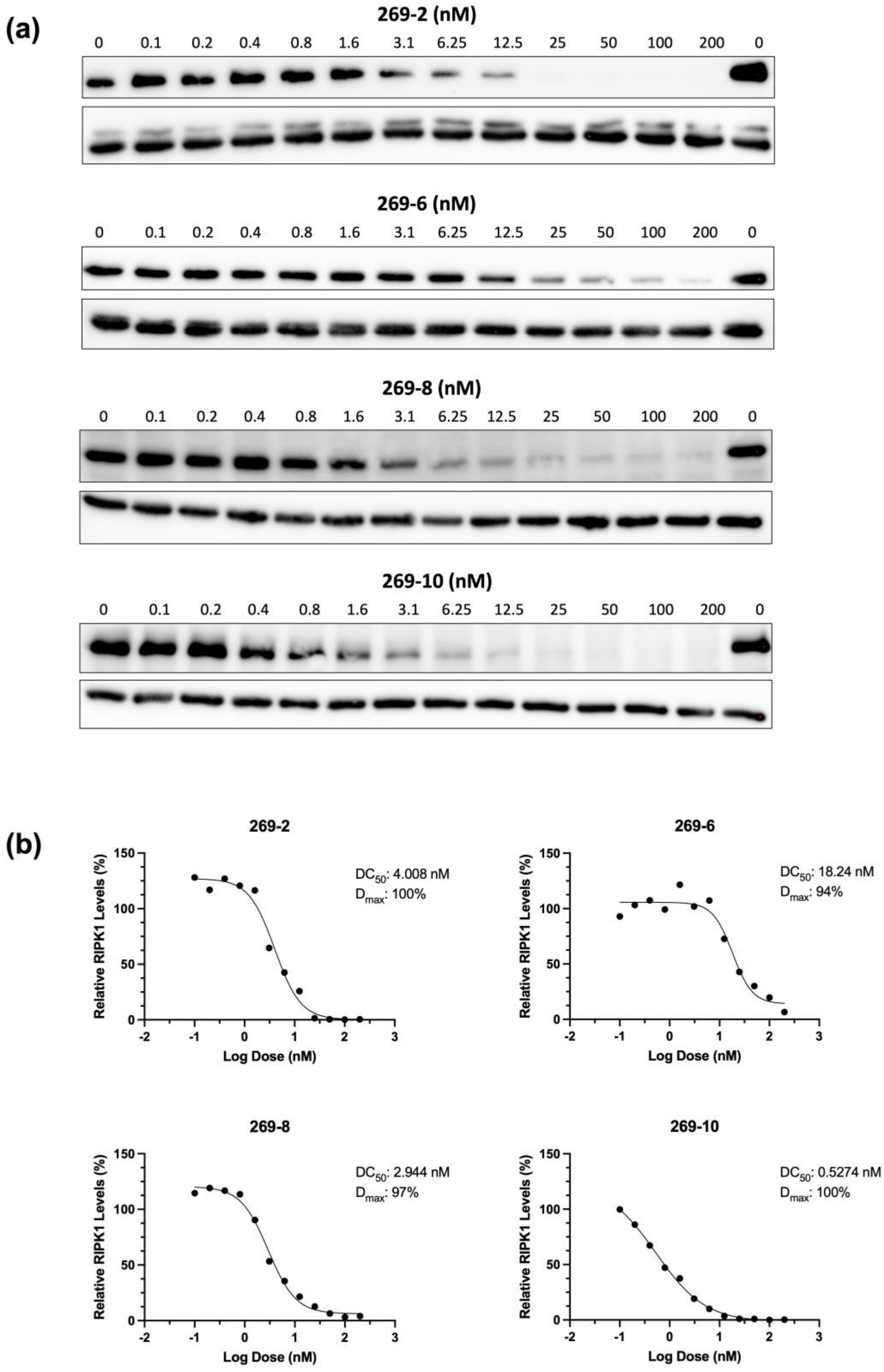

Figure S16. Degradation activities for Site 3 linkers. (a) Raw western blot data for the four Site 3 linker compounds in the 269 series. **(b)** Dose–response curves for the four Site 3 linker compounds in the 269 series. RIPK1 degradation activity was tested with 24 h treatment time in PC3 cells.

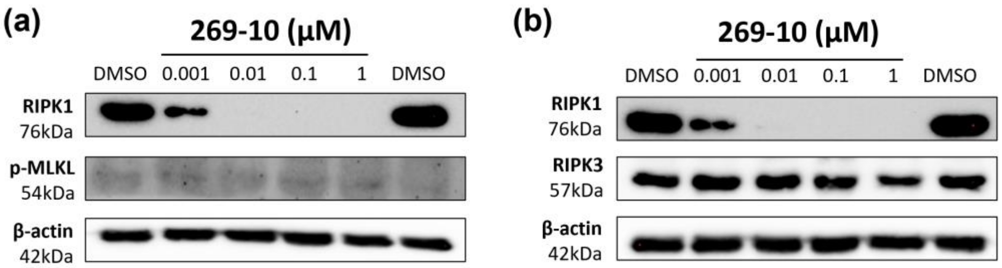

Figure S17. Dose-response to 269-10 for RIPK1, p-MLKL, and RIPK3. (a) Comparison of degradation effects of **269-10** between RIPK1 and p-MLKL. **(b)** Comparison of degradation effects of **269-10** between RIPK1 and RIPK3. PC3 cells were treated with the RIPK1 PROTAC **269-10** at listed concentrations for 24 hours. Following treatment, cells were harvested and lysates collected and analyzed by Western blot.

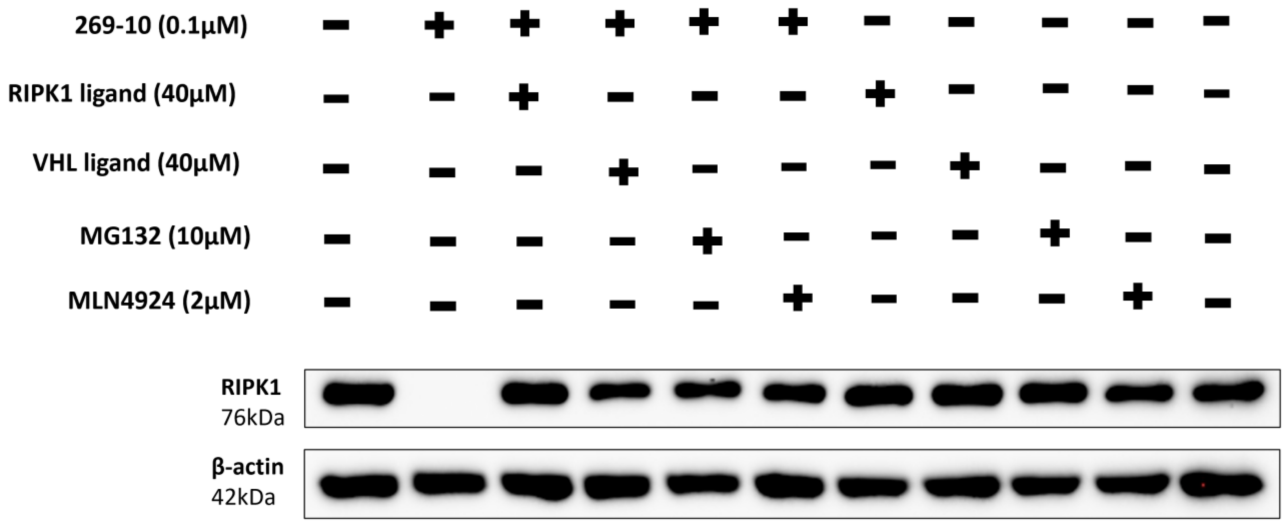

Figure S18. Mechanism of degrader 269-10 in PC3 cells. PC3 cells were treated with either the free RIPK1 ligand (40 μM), VHL ligand (40 μM), proteosome inhibitor MG132 (10 μM) or neddylation inhibitor MLN4924 (2 μM) for 1 hour. Then the treatment group received RIPK1 PROTAC **269-10** (0.1 μM) for 3 hours. After the treatment time, the cell lysates were collected and RIPK1 protein levels were observed by Western blot.

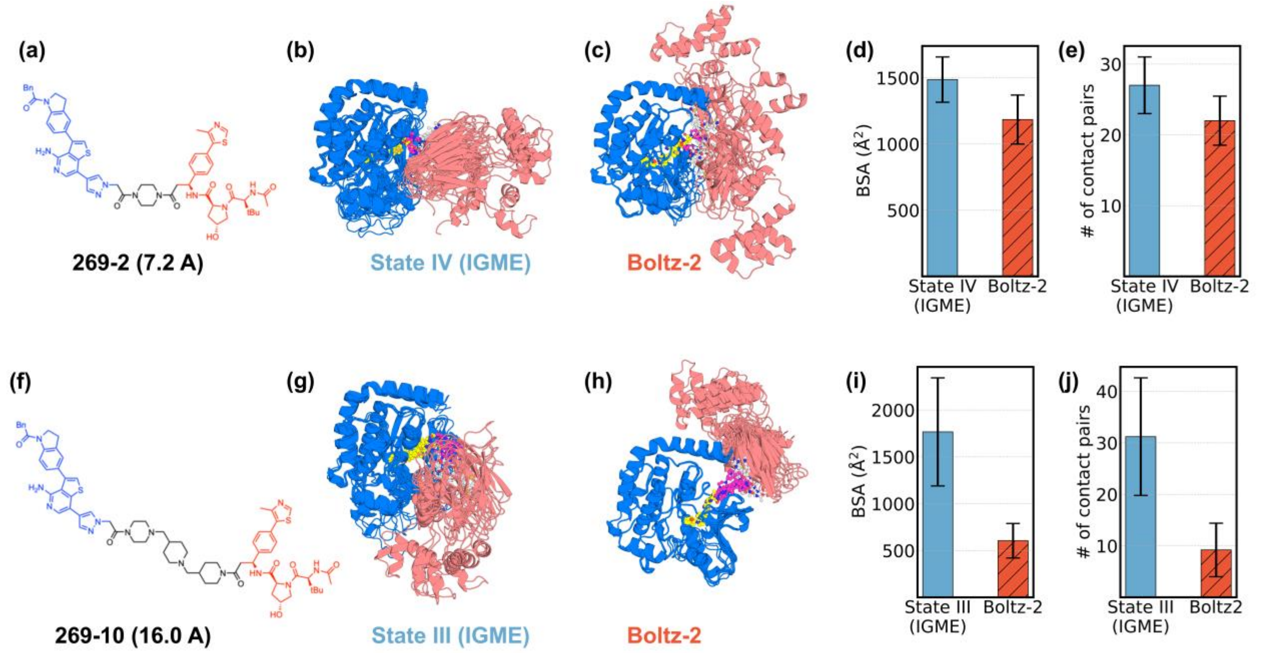

Figure S19. Comparison of ternary complex structures obtained from IGME modeling and Boltz-2. (a) Chemical structure of compound **269-2**; linker length is indicated in parentheses (see **Figure S15**). **(b)** Representative ternary complex structures from State IV modeled with **269-2**. Structure selection follows the same criteria used for binding free energy calculations (see SI text Sec. 5). All structures are aligned to RIPK1. **(c)** Ternary complex structures obtained from Boltz-2 co-folding with **269-2**. **(d)** BSA results of the modeled protein complexes from the different methods with structures shown in **(b)** and **(c)**. **(e)** Number of RIPK1–VHL residue contact pairs for the structures shown in **(b)** and **(c)**. **(f)** Chemical structure of compound **269-10**; linker length is indicated in parentheses. **(g)** Representative ternary complex structures from State III modeled with **269-10**. Structure selection follows the same criteria used for binding free energy calculations. **(h)** Ternary complex structures obtained from Boltz-2 co-folding with **269-10**. **(i)** BSA results of the modeled protein complexes from the different methods with structures shown in **(g)** and **(h)**. **(j)** Number of RIPK1–VHL residue contact pairs for the structures shown in **(g)** and **(h)**. For chemical structures, the POI ligand is shown in blue, the linker in black, and the E3 ligand in red. The fluorocyclopropyl group at Site 3 was not modeled and is therefore not shown. For ternary complex structures, RIPK1 is shown in marine, VHL in salmon, the POI ligand in yellow (stick-and-ball), the E3 ligand in gray (stick-and-ball), and the linker motif in magenta (stick-and-ball).

Analytical Characterization Data

1. NMR Data

(2S,4R)-N-((S)-3-(4-(2-(4-(4-amino-3-(1-(2-phenylacetyl)indolin-5-yl)thieno[3,2-c]pyridin-7-yl)-1H-pyrazol-1-yl)acetyl)piperazin-1-yl)-1-(4-(4-methylthiazol-5-yl)phenyl)-3-oxopropyl)-1-((S)-2-(1-fluorocyclopropane-1-carboxamido)-3,3-dimethylbutanoyl)-4-hydroxypyrrolidine-2-carboxamide (**269-2**)
^{1}H NMR (400 MHz, Chloroform-*d*) δ 8.66 (s, 1H), 8.36 (d, *J* = 8.29 Hz, 1H), 8.01 – 7.90 (m, 2H), 7.86 (d, *J* = 6.34 Hz, 1H), 7.75 (s, 1H), 7.43 – 7.18 (m, 13H), 7.12 (dd, *J* = 8.83, 3.47 Hz, 2H), 5.40 (d, *J* = 7.18 Hz, 1H), 5.12 – 5.03 (m, 2H), 4.64 (dd, *J* = 24.60, 8.69 Hz, 2H), 4.52 – 4.45 (m, 1H), 4.17 (t, *J* = 8.48 Hz, 3H), 4.11 – 3.92 (m, 6H), 3.86 (s, 2H), 3.69 (dd, *J* = 11.36, 3.63 Hz, 1H), 3.57 (d, *J* = 12.19 Hz, 5H), 3.44 (s, 2H), 3.25 (d, *J* = 8.74 Hz, 2H), 3.06 (dd, *J* = 15.51, 5.44 Hz, 1H), 2.95 – 2.84 (m, 1H), 2.50 (s, 3H), 2.25 – 2.14 (m, 2H), 1.36 – 1.20 (m, 5H), 1.07 (s, 9H).
^{13}C NMR (101 MHz, Chloroform-*d*) δ 170.7, 170.6, 170.2, 170.0, 169.6, 168.9, 168.7, 165.0, 151.7, 150.4, 150.0, 148.4, 143.9, 140.7, 138.7, 137.8, 133.8, 132.3, 131.4, 131.1, 129.5, 129.5, 129.0, 128.8, 128.8, 127.1, 126.9, 125.4, 124.6, 119.8, 117.5, 117.3, 70.0, 59.3, 57.5, 56.8, 53.5, 53.4, 50.1, 48.4, 45.6, 45.2, 44.9, 43.5, 41.9, 41.3, 38.2, 37.1, 35.7, 29.6, 27.9, 26.4, 16.0, 13.8, 13.7, 13.6.
HRMS-ESI (m/z): $[M+H^{+}]^{+}$ calculated for $C_{60}H_{65}FN_{11}O_{7}S_{2}$, 1134.4488; found, 1134.4477;
HPLC retention time: 5.67 min, purity >95%.

(2S,4R)-N-((S)-3-((2-(4-(2-(4-(4-amino-3-(1-(2-phenylacetyl)indolin-5-yl)thieno[3,2-c]pyridin-7-yl)-1H-pyrazol-1-yl)acetyl)piperazin-1-yl)ethyl)amino)-1-(4-(4-methylthiazol-5-yl)phenyl)-3-oxopropyl)-1-((S)-2-(1-fluorocyclopropane-1-carboxamido)-3,3-dimethylbutanoyl)-4-hydroxypyrrolidine-2-carboxamide (**269-6**)
^{1}H NMR (400 MHz, Chloroform-*d*) δ 8.66 (s, 1H), 8.46 (s, 1H), 8.36 (d, *J* = 8.22 Hz, 1H), 8.10 (d, *J* = 8.19 Hz, 1H), 7.88 (d, *J* = 20.05 Hz, 2H), 7.72 (s, 1H), 7.44 – 7.16 (m, 16H), 7.14 – 7.04 (m, 3H), 5.38 (d, *J* = 6.97 Hz, 1H), 5.10 – 4.96 (m, 2H), 4.68 – 4.57 (m, 3H), 4.51 (s, 1H), 4.41 – 4.06 (m, 13H), 3.99 (d, *J* = 11.21 Hz, 1H), 3.86 (s, 2H), 3.72 (dd, *J* = 11.30, 3.46 Hz, 2H), 3.56 (d, *J* = 38.20 Hz, 6H), 3.35 – 3.16 (m, 4H), 2.78 (d, *J* = 5.76 Hz, 2H), 2.51 (d, *J* = 12.05 Hz, 10H), 2.26 – 2.08 (m, 3H), 1.37 – 1.20 (m, 5H), 1.07 (s, 10H).
^{13}C NMR (101 MHz, Chloroform-*d*) δ 170.8, 170.6, 170.1, 170.0, 169.8, 169.6, 165.0, 151.6, 150.4, 150.0, 148.4, 144.0, 140.4, 138.7, 137.7, 133.8, 132.3, 131.4, 131.0, 130.0, 129.5, 129.3, 129.0, 128.8, 128.8, 127.1, 126.8, 125.4, 124.8, 119.8, 117.3, 117.3, 114.1, 77.2, 70.0, 59.7, 57.5, 57.0, 56.8, 53.3, 52.3, 50.2, 48.4, 44.5, 43.5, 41.8, 41.6, 37.4, 35.8, 35.7, 27.9, 26.4, 16.1, 13.8, 13.7, 13.5, 13.4.
HRMS-ESI (m/z): $[M+H^{+}]^{+}$ calculated for $C_{62}H_{70}FN_{12}O_{7}S_{2}$, 1177.4910; found, 1177.4903;
HPLC retention time: 4.96 min, purity >95%.

(2S,4R)-N-((S)-3-(4-((4-(2-(4-(4-amino-3-(1-(2-phenylacetyl)indolin-5-yl)thieno[3,2-c]pyridin-7-yl)-1H-pyrazol-1-yl)acetyl)piperazin-1-yl)methyl)piperidin-1-yl)-1-(4-(4-methylthiazol-5-yl)phenyl)-3-oxopropyl)-1-((S)-2-(1-fluorocyclopropane-1-carboxamido)-3,3-dimethylbutanoyl)-4-hydroxypyrrolidine-2-carboxamide (**269-8**)
^{1}H NMR (400 MHz, Chloroform-*d*) δ 8.68 (s, 1H), 8.38 (d, *J* = 8.32 Hz, 2H), 8.08 (dd, *J* = 70.43, 7.81 Hz, 1H), 7.90 (d, *J* = 25.91 Hz, 2H), 7.72 (s, 1H), 7.42 – 7.24 (m, 13H), 7.23 (s, 1H), 7.14 (q, *J* = 6.60, 5.01 Hz, 1H), 5.38 (d, *J* = 6.72 Hz, 1H), 5.08 (d, *J* = 2.68 Hz, 2H), 4.73 – 4.59 (m, 2H), 4.52 (t, *J* = 8.39 Hz, 2H), 4.18 (t, *J* = 8.48 Hz, 3H), 4.08 – 3.81 (m, 11H), 3.76 – 3.47 (m, 7H), 3.24 (s, 2H), 3.09 – 2.75 (m, 4H), 2.55 – 2.30 (m, 8H), 2.27 – 2.02 (m, 4H), 1.70 (dd, *J* = 31.94, 13.03 Hz, 3H), 1.38 – 1.20 (m, 5H), 1.08 (s, 10H), 0.95 – 0.79 (m, 2H), 0.55 (d, *J* = 12.79 Hz, 1H).

^{13}C NMR (101 MHz, Chloroform-*d*) δ 170.6, 170.5, 170.3, 170.1, 169.6, 168.5, 168.3, 164.5, 151.5, 150.4, 150.4, 148.3, 144.1, 141.2, 141.1, 138.8, 137.7, 133.8, 132.3, 131.5, 130.8, 129.4, 129.0, 128.9, 128.8, 127.1, 126.8, 125.3, 125.0, 119.9, 117.4, 117.2, 114.1, 70.1, 63.8, 59.2, 57.4, 56.7, 53.5, 52.7, 50.2, 48.4, 46.1, 45.8, 45.3, 43.5, 42.2, 41.8, 37.4, 37.1, 35.6, 33.4, 33.2, 30.9, 30.2, 27.9, 26.5, 16.1, 16.1, 13.7, 13.6.
HRMS-ESI (m/z): $[M+H^+]^+$ calculated for $C_{66}H_{76}FN_{12}O_7S_2$, 1231.5380; found, 1231.5359;
HPLC retention time: 4.95 min, purity >95%.

(2S,4R)-N-((S)-3-(4-((4-((4-(2-(4-(4-amino-3-(1-(2-phenylacetyl)indolin-5-yl)thieno[3,2-c]pyridin-7-yl)-1H-pyrazol-1-yl)acetyl)piperazin-1-yl)methyl)piperidin-1-yl)methyl)piperidin-1-yl)-1-(4-(4-methylthiazol-5-yl)phenyl)-3-oxopropyl)-1-((S)-2-(1-fluorocyclopropane-1-carboxamido)-3,3-dimethylbutanoyl)-4-hydroxypyrrolidine-2-carboxamide (**269-10**)
^1H NMR (500 MHz, Chloroform-*d*) δ 8.71 (s, 1H), 8.49 (s, 1H), 8.41 (d, *J* = 8.26 Hz, 1H), 8.09 (d, *J* = 7.64 Hz, 1H), 7.94 (d, *J* = 24.57 Hz, 2H), 7.82 (s, 1H), 7.46 – 7.25 (m, 15H), 7.14 (dt, *J* = 8.37, 3.73 Hz, 1H), 6.78 (s, 1H), 5.44 – 5.36 (m, 1H), 5.12 (s, 2H), 4.73 (td, *J* = 8.18, 4.26 Hz, 1H), 4.65 (d, *J* = 8.82 Hz, 1H), 4.54 (d, *J* = 9.74 Hz, 2H), 4.21 (t, *J* = 8.52 Hz, 2H), 4.04 – 3.97 (m, 1H), 3.91 (s, 2H), 3.84 – 3.72 (m, 3H), 3.68 (dt, *J* = 9.52, 6.11 Hz, 2H), 3.59 (d, *J* = 5.08 Hz, 2H), 3.33 – 2.77 (m, 21H), 2.59 – 2.48 (m, 5H), 2.43 (s, 4H), 2.30 (d, *J* = 39.15 Hz, 5H), 1.94 – 1.75 (m, 5H), 1.65 – 1.53 (m, 3H), 1.49 (d, *J* = 6.62 Hz, 1H), 1.40 – 1.22 (m, 9H), 1.11 (s, 11H), 0.94 – 0.81 (m, 3H).
^{13}C NMR (126 MHz, Chloroform-*d*) δ 170.8, 170.6, 170.5, 170.4, 169.5, 168.5, 168.3, 164.6, 152.0, 150.3, 150.3, 149.7, 148.4, 143.9, 141.2, 141.1, 138.5, 137.7, 133.8, 132.2, 131.9, 131.6, 130.9, 130.8, 130.0, 129.4, 129.0, 128.8, 128.6, 127.2, 126.8, 126.8, 125.4, 124.3, 119.8, 117.8, 117.3, 114.2, 79.1, 70.1, 70.0, 63.4, 59.1, 57.5, 57.4, 56.6, 53.6, 53.6, 53.4, 52.8, 50.4, 50.1, 48.4, 45.7, 45.5, 45.4, 43.6, 42.3, 41.5, 38.0, 37.5, 37.1, 37.0, 35.6, 35.5, 32.5, 32.4, 32.2, 31.1, 30.9, 30.3, 29.7, 28.4, 28.0, 26.5, 26.5, 16.2, 16.1, 13.7, 13.6.
HRMS-ESI (m/z): $[M+H^+]^+$ calculated for $C_{72}H_{87}FN_{13}O_7S_2$, 1328.6271; found, 1328.6231;
HPLC retention time: 4.47 min, purity >95%.

2. NMR Spectrum

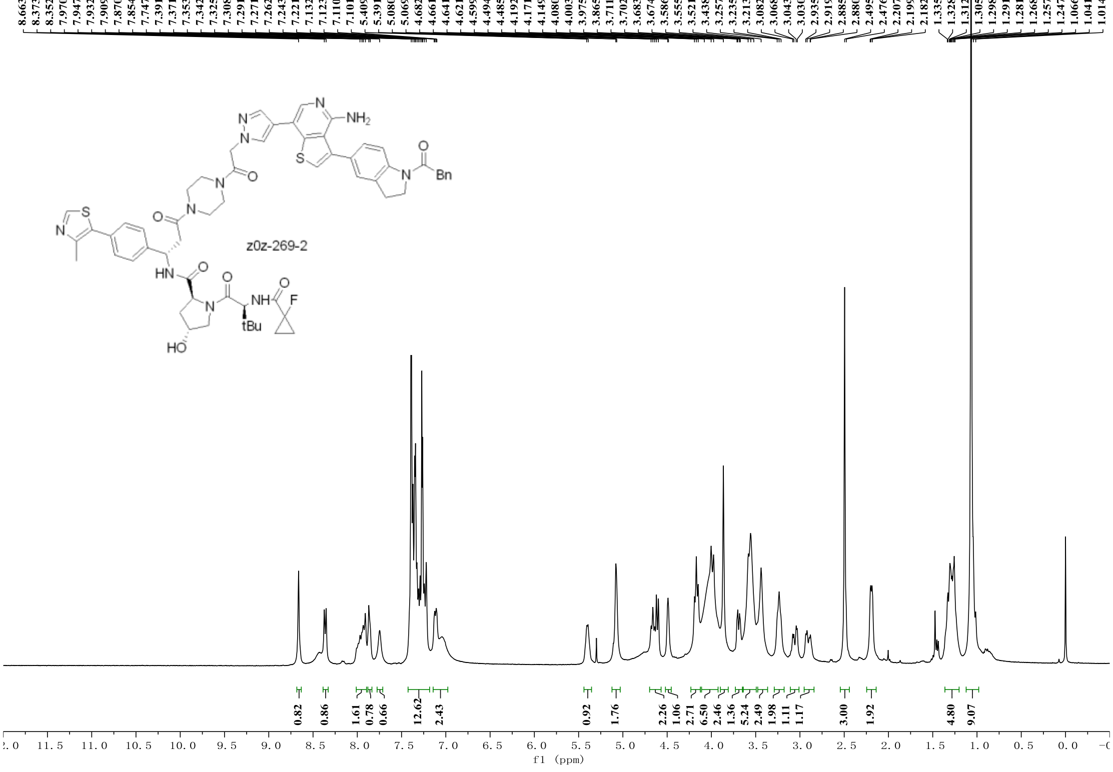

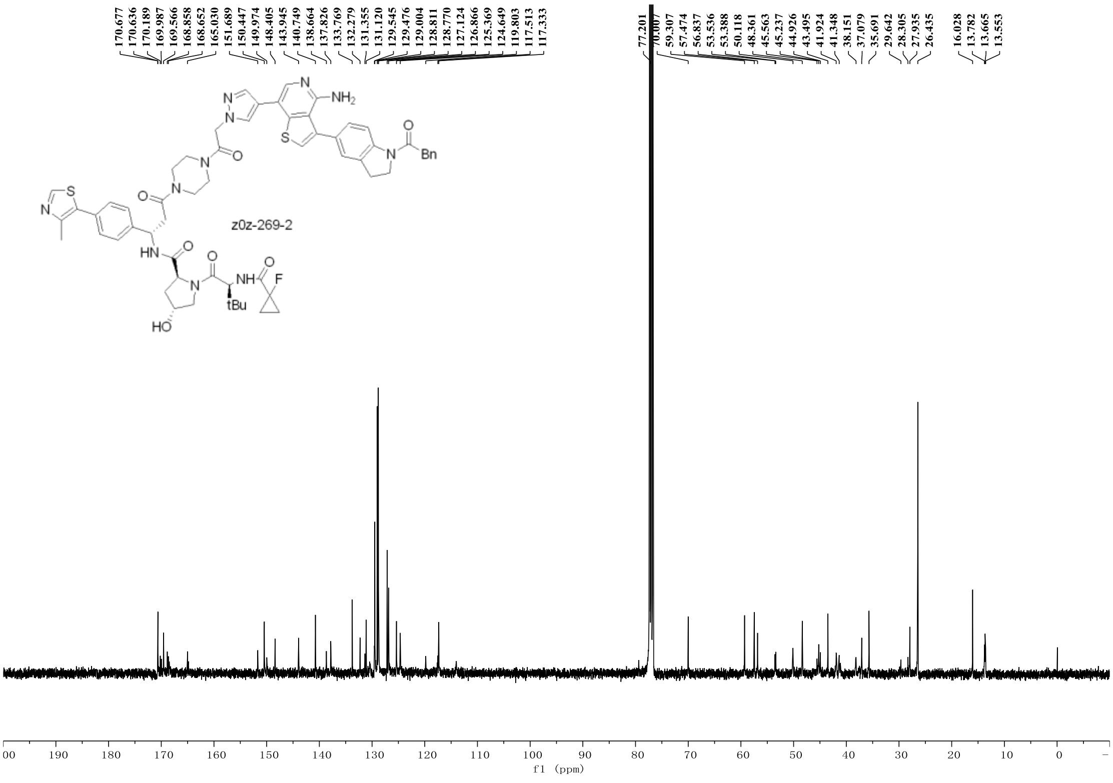

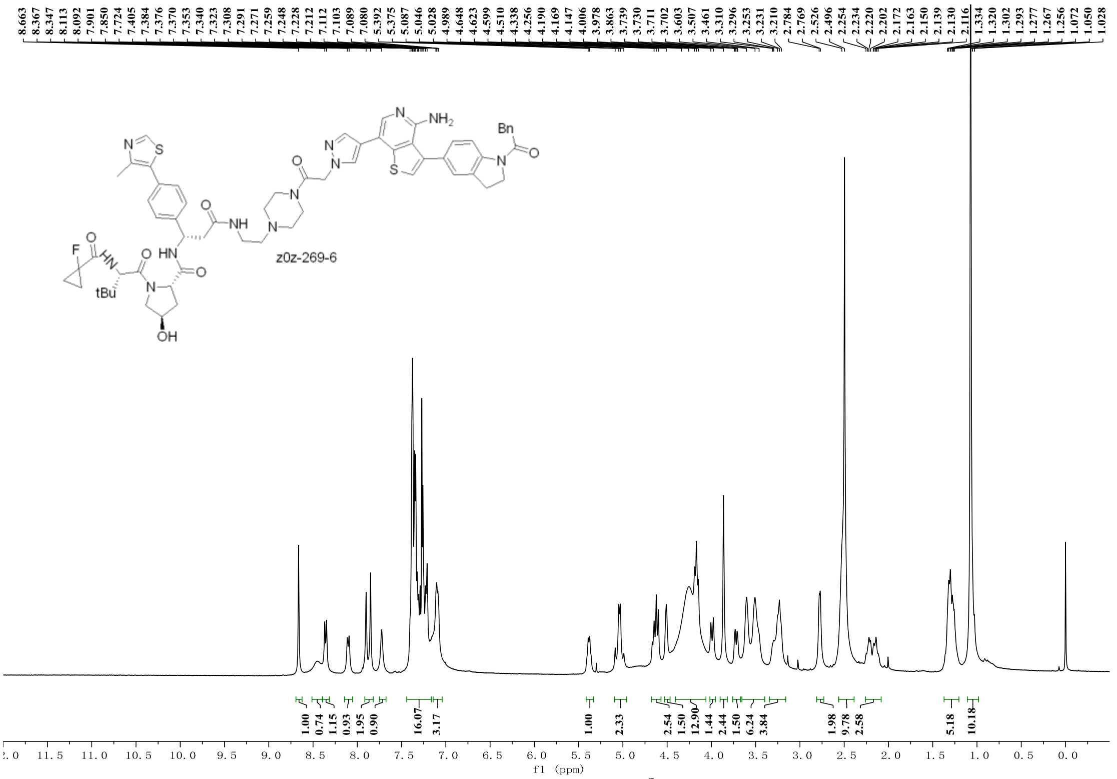
z0z-269-6
f1 (ppm)

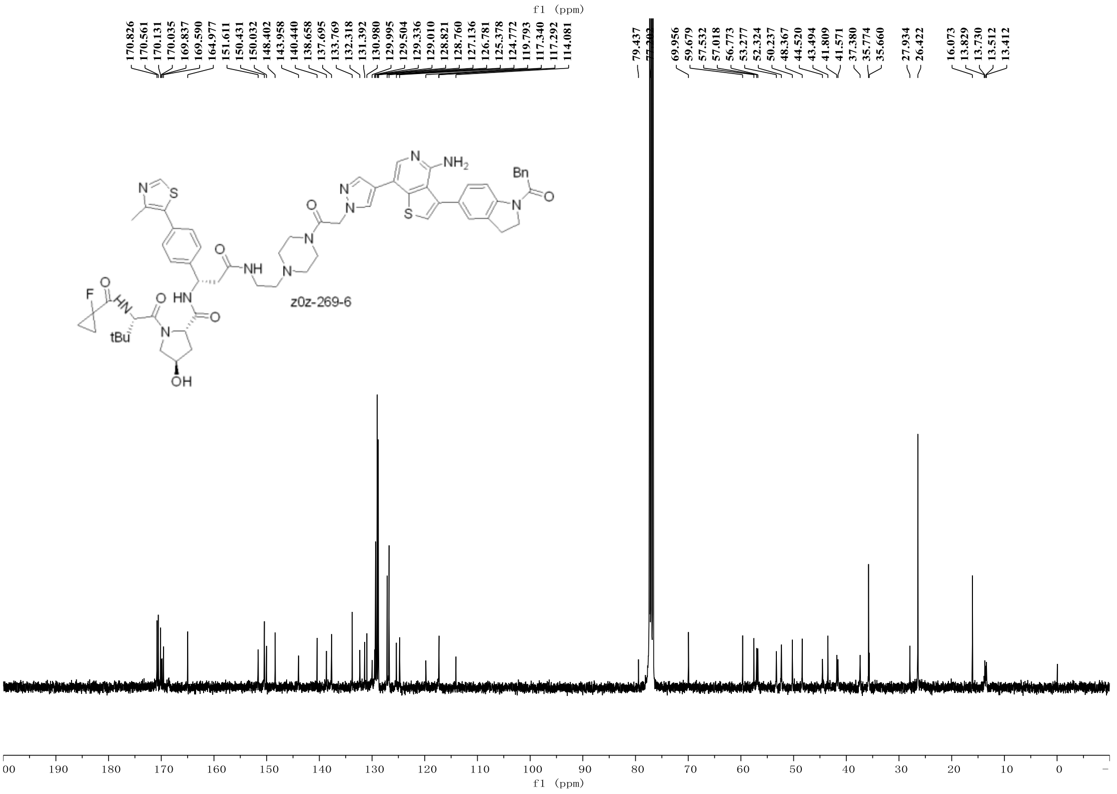
z0z-269-6
f1 (ppm)

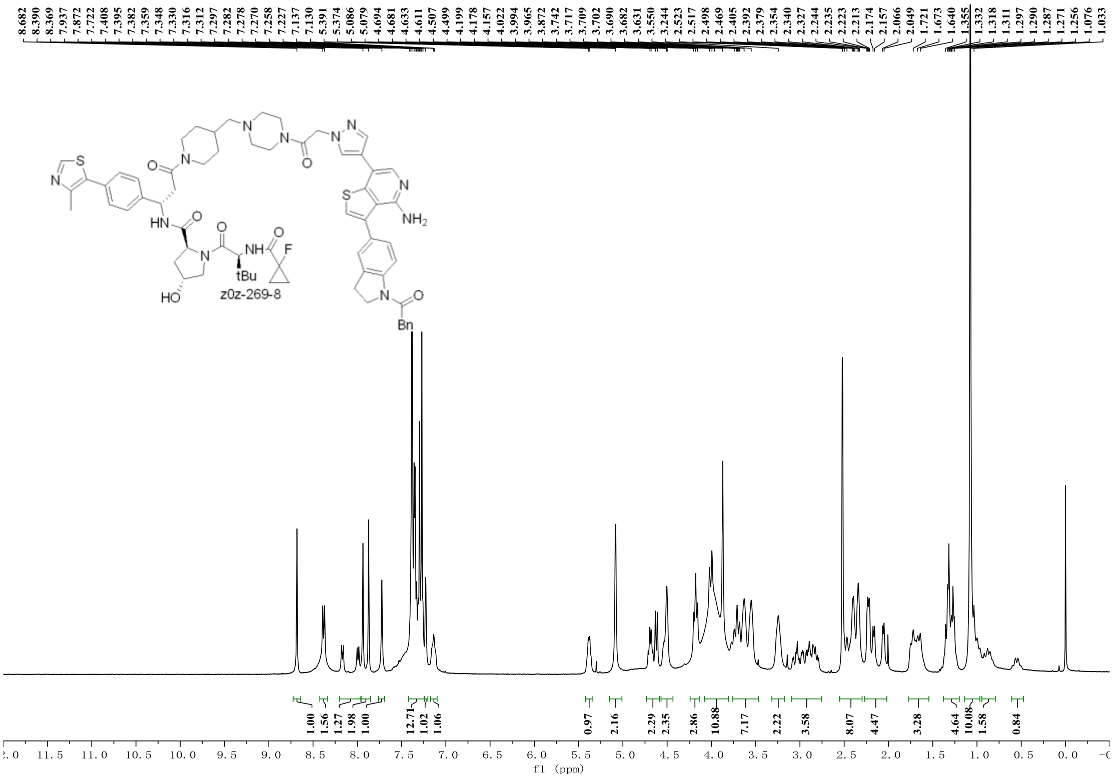
8.682 8.390 8.369 7.937 7.872 7.722 7.408 7.395 7.382 7.359 7.348 7.330 7.316 7.312 7.297 7.282 7.278 7.270 7.258 7.227 7.137 7.130 5.391 5.374 5.086 5.079 4.694 4.681 4.633 4.611 4.507 4.499 4.199 4.178 4.157 4.022 3.994 3.965 3.872 3.742 3.717 3.709 3.702 3.690 3.682 3.631 3.550 3.244 2.517 2.498 2.469 2.405 2.392 2.379 2.354 2.340 2.327 2.244 2.235 2.223 2.213 2.174 2.157 2.066 2.049 1.721 1.673 1.640 1.355 1.332 1.318 1.311 1.297 1.290 1.287 1.271 1.256 1.076 1.033
z0z-269-8
1.00 1.56 1.27 1.98 1.00 12.71 1.02 1.06 0.97 2.16 2.29 2.35 2.86 10.88 7.17 2.22 3.58 8.07 4.47 3.28 4.64 10.08 1.58 0.84
f1 (ppm)

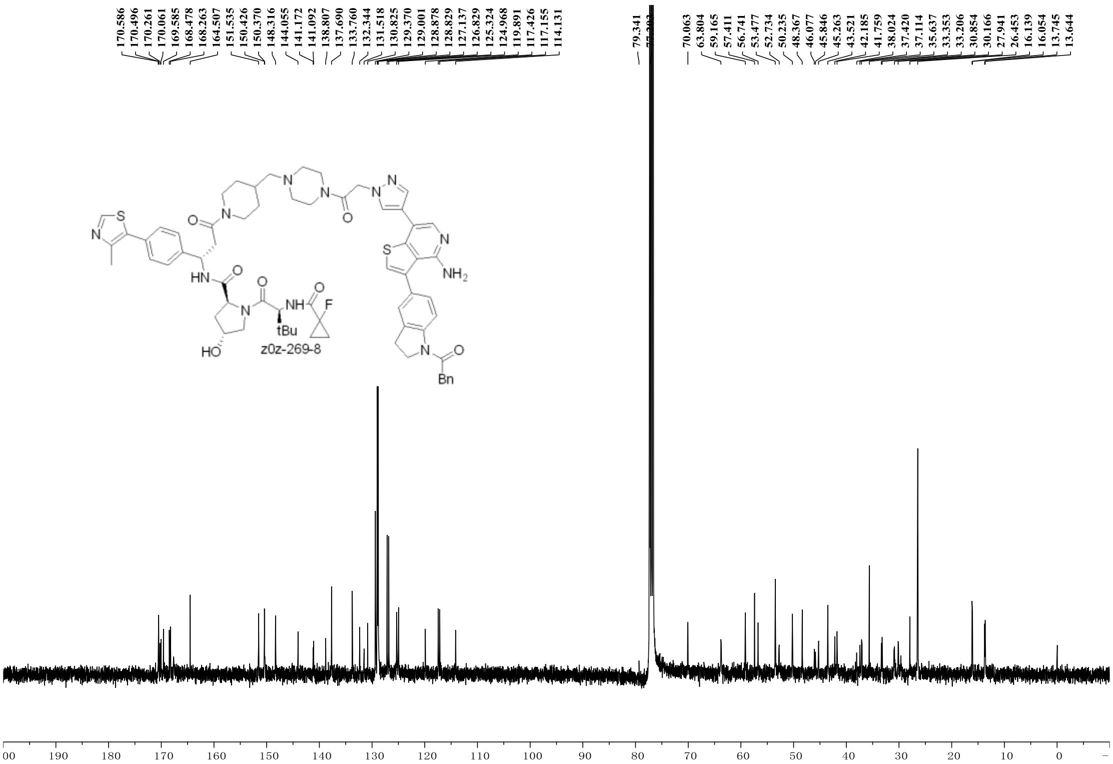
170.586 170.496 170.261 170.061 169.585 168.478 168.263 164.507 151.535 150.426 150.370 148.316 144.055 141.172 141.092 138.807 137.690 133.760 132.344 131.518 130.825 129.370 129.001 128.878 128.829 127.137 126.829 125.324 124.968 119.891 117.426 117.155 114.131
79.341
70.063 63.804 59.165 57.411 56.741 53.477 52.734 50.235 48.367 46.077 45.846 45.263 43.521 42.185 41.759 38.024 37.420 37.114 35.637 33.353 33.206 30.854 30.166 27.941 26.453 16.139 16.054 13.745 13.644
z0z-269-8
f1 (ppm)

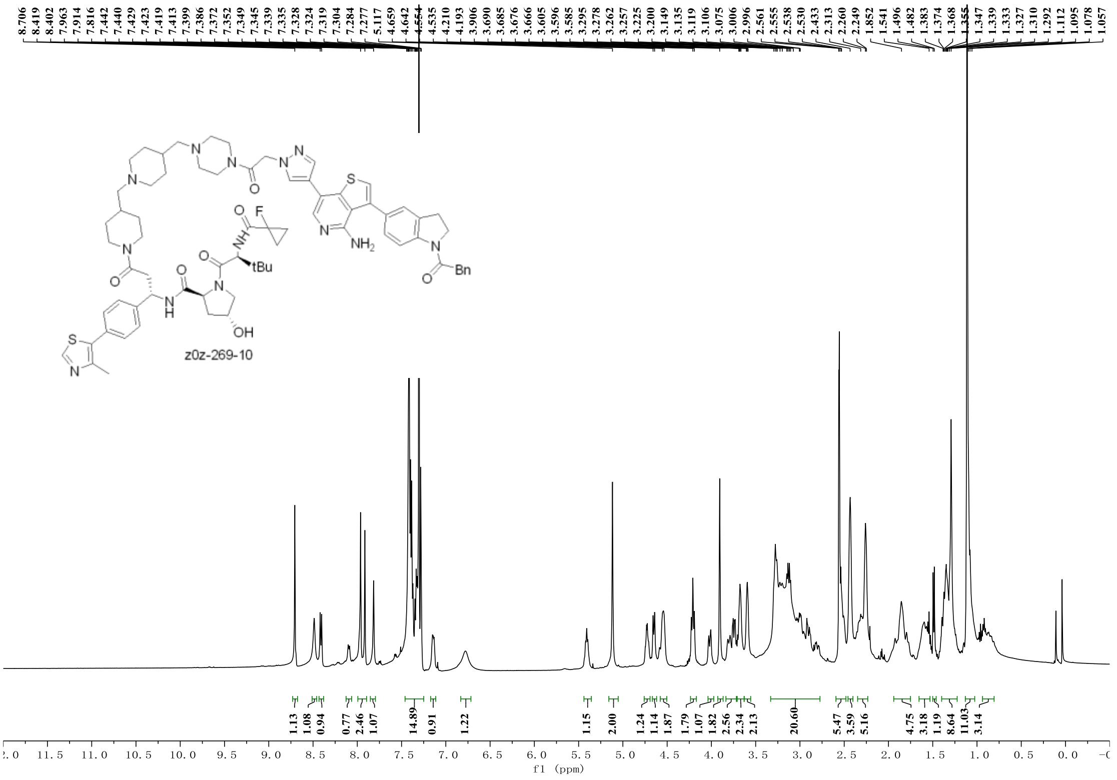
8.706 8.419 8.402 7.963 7.914 7.816 7.442 7.440 7.429 7.423 7.419 7.413 7.399 7.386 7.372 7.352 7.349 7.345 7.339 7.335 7.328 7.324 7.319 7.304 7.284 7.277 5.117 4.659 4.642 4.535 4.210 4.193 3.906 3.690 3.685 3.676 3.666 3.605 3.596 3.585 3.295 3.278 3.262 3.257 3.225 3.200 3.149 3.135 3.119 3.106 3.075 3.006 2.996 2.561 2.555 2.538 2.530 2.433 2.313 2.260 2.249 1.852 1.541 1.496 1.482 1.383 1.374 1.368 1.355 1.347 1.339 1.333 1.327 1.310 1.292 1.112 1.095 1.078 1.057
z0z-269-10
1.13 1.08 0.94 0.77 2.46 1.07 14.89 0.91 1.22 1.15 2.00 1.24 1.14 1.87 1.79 1.07 1.82 2.56 2.34 2.13 20.60 5.47 3.59 5.16 4.75 3.18 1.19 8.64 11.03 3.14
f1 (ppm)

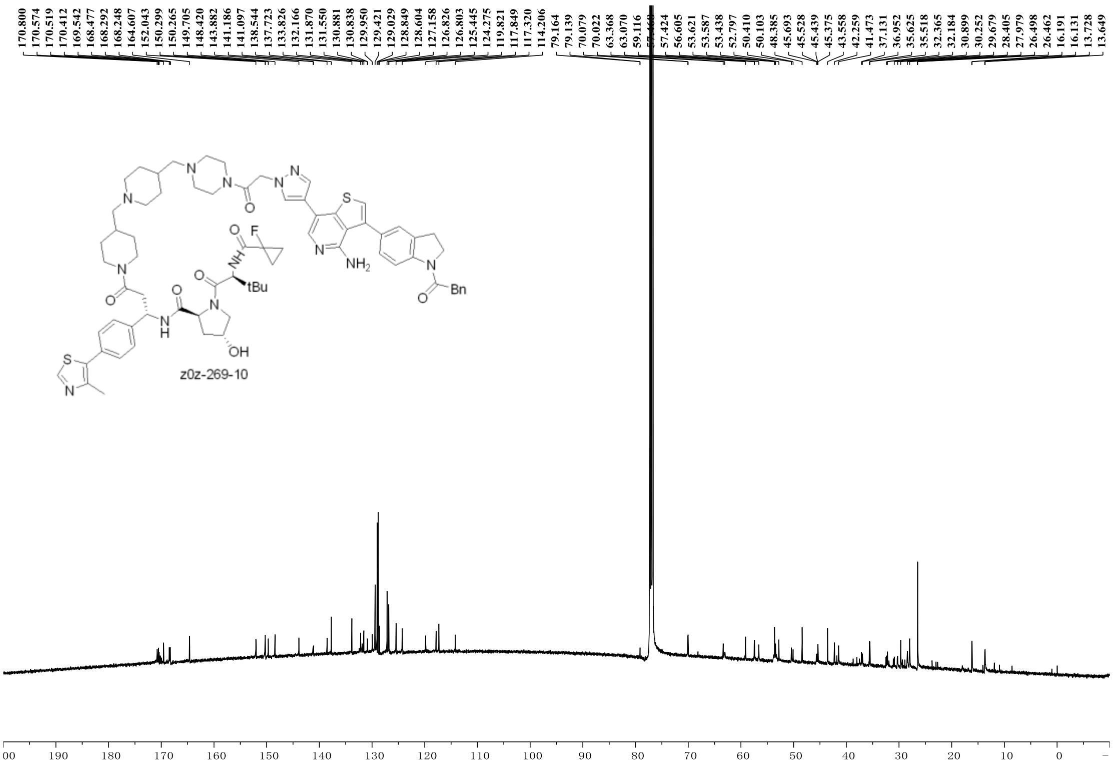
170.800 170.574 170.519 170.412 169.542 168.477 168.292 168.248 164.607 152.043 150.299 150.265 149.705 148.420 143.882 141.186 141.097 138.544 137.723 133.826 132.166 131.870 131.550 130.881 130.838 129.950 129.421 129.029 128.849 128.604 127.158 126.826 126.803 125.445 124.275 119.821 117.849 117.320 114.206 79.164 79.139 70.079 70.022 63.368 63.070 59.116 57.424 56.605 53.621 53.587 53.438 52.797 50.410 50.103 48.385 45.693 45.528 45.439 45.375 43.558 42.259 41.473 37.131 36.952 35.625 35.518 32.365 32.184 30.899 30.252 29.679 28.405 27.979 26.498 26.462 16.191 16.131 13.728 13.649
z0z-269-10
f1 (ppm)

3. HPLC Data

<Sample Information>

Sample Name	: 269-2-11		
Sample ID	:		
Data Filename	: 269-2-11.lcd		
Method Filename	: analytic_15min_0.8ml_min_20min_total_9.0_slope.lcm		
Batch Filename	: normal batch.lcb		
Vial #	: 1-11	Sample Type	: Unknown
Injection Volume	: 10 uL		
Date Acquired	: 5/29/2025 6:19:56 PM	Acquired by	: System Administrator
Date Processed	: 5/29/2025 6:34:56 PM	Processed by	: System Administrator

<Chromatogram>

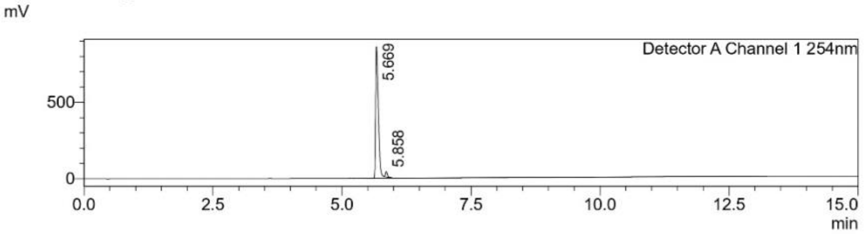

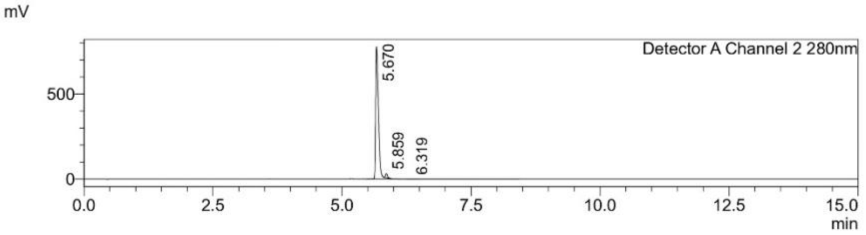

<Peak Table>

Detector A Channel 1 254nm

Peak#	Ret. Time	Height	Width at 5% Height	Area	Area%
1	5.669	844343	0.125	3065636	97.221
2	5.858	33789	0.087	87636	2.779
Total		878132		3153272	100.000

Detector A Channel 2 280nm

Peak#	Ret. Time	Height	Width at 5% Height	Area	Area%
1	5.670	768581	0.125	2968466	97.900
2	5.859	24499	0.081	61879	2.041
3	6.319	331	0.200	1785	0.059
Total		793410		3032129	100.000

C:\LabSolutions\Data\zhen\269-2-11.lcd

<Sample Information>

Sample Name	: 296-6-43		
Sample ID	:		
Data Filename	: 296-6-43.lcd		
Method Filename	: analytic_15min_0.8ml_min_20min_total_9.0_slope.lcm		
Batch Filename	: normal batch.lcb		
Vial #	: 1-43	Sample Type	: Unknown
Injection Volume	: 10 uL		
Date Acquired	: 5/30/2025 11:49:12 AM	Acquired by	: System Administrator
Date Processed	: 5/30/2025 12:04:12 PM	Processed by	: System Administrator

<Chromatogram>

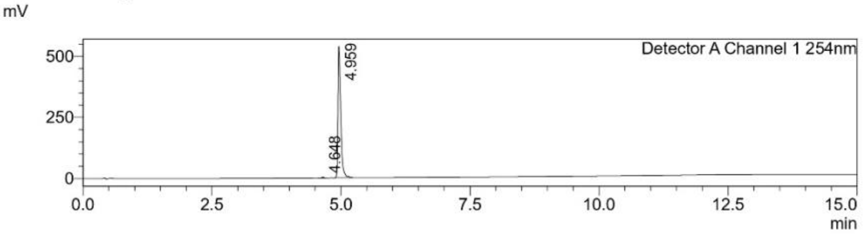

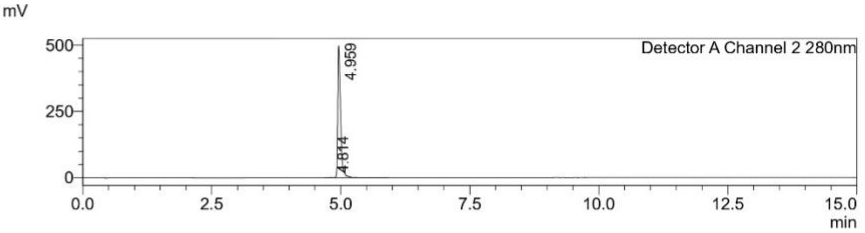

<Peak Table>

Detector A Channel 1 254nm

Peak#	Ret. Time	Height	Width at 5% Height	Area	Area%
1	4.648	3658	0.085	8637	0.428
2	4.959	526204	0.136	2011435	99.572
Total		529862		2020071	100.000

Detector A Channel 2 280nm

Peak#	Ret. Time	Height	Width at 5% Height	Area	Area%
1	4.814	1319	--	5118	0.271
2	4.959	485467	0.137	1885745	99.729
Total		486786		1890863	100.000

C:\LabSolutions\Data\zhen\296-6-43.lcd

<Sample Information>

Sample Name	: 296-8-53		
Sample ID	:		
Data Filename	: 296-8-53.lcd		
Method Filename	: analytic_15min_0.8ml_min_20min_total_9.0_slope.lcm		
Batch Filename	: normal batch.lcb		
Vial #	: 1-52	Sample Type	: Unknown
Injection Volume	: 10 uL		
Date Acquired	: 5/30/2025 1:10:10 PM	Acquired by	: System Administrator
Date Processed	: 5/30/2025 1:25:11 PM	Processed by	: System Administrator

<Chromatogram>

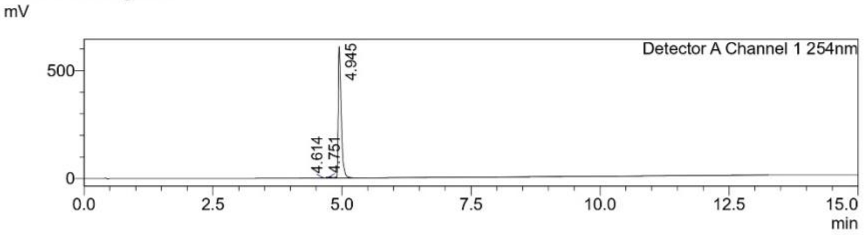

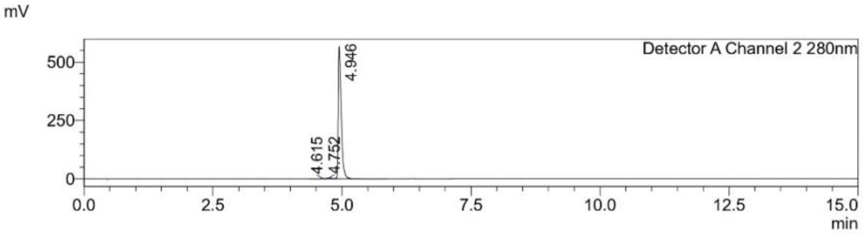

<Peak Table>

Detector A Channel 1 254nm

Peak#	Ret. Time	Height	Width at 5% Height	Area	Area%
1	4.614	2986	--	10037	0.464
2	4.751	4490	--	26988	1.248
3	4.945	602505	0.146	2124762	98.287
Total		609981		2161786	100.000

Detector A Channel 2 280nm

Peak#	Ret. Time	Height	Width at 5% Height	Area	Area%
1	4.615	2939	--	11168	0.488
2	4.752	4455	--	27053	1.182
3	4.946	561181	0.144	2249660	98.329
Total		568576		2287881	100.000

C:\LabSolutions\Data\zhen\296-8-53.lcd

<Sample Information>

Sample Name	: 296-10-28		
Sample ID	:		
Data Filename	: 296-10-28.lcd		
Method Filename	: analytic_15min_0.8ml_min_20min_total_9.0_slope.lcm		
Batch Filename	: normal batch.lcb		
Vial #	: 1-28	Sample Type	: Unknown
Injection Volume	: 10 uL		
Date Acquired	: 5/30/2025 2:10:46 PM	Acquired by	: System Administrator
Date Processed	: 5/30/2025 2:25:47 PM	Processed by	: System Administrator

<Chromatogram>

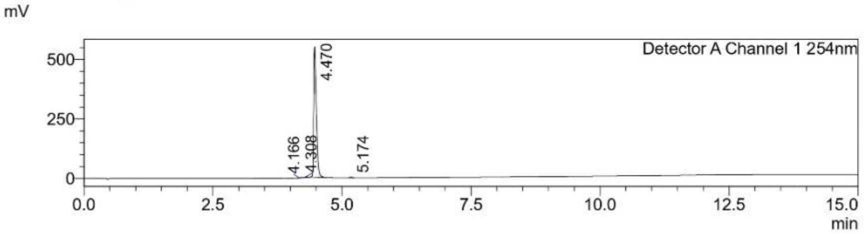

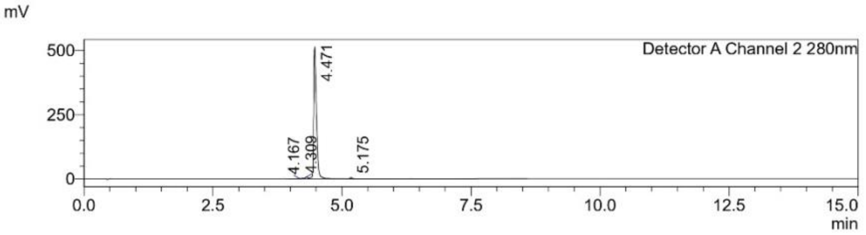

<Peak Table>

Detector A Channel 1 254nm

Peak#	Ret. Time	Height	Width at 5% Height	Area	Area%
1	4.166	1385	--	2919	0.146
2	4.308	4364	0.125	12882	0.645
3	4.470	540374	0.123	1973890	98.752
4	5.174	3468	0.084	9136	0.457
Total		549591		1998827	100.000

Detector A Channel 2 280nm

Peak#	Ret. Time	Height	Width at 5% Height	Area	Area%
1	4.167	2085	--	7848	0.395
2	4.309	7014	--	34877	1.755
3	4.471	508546	0.126	1930328	97.125
4	5.175	5009	0.098	14423	0.726
Total		522655		1987475	100.000

C:\LabSolutions\Data\zhen\296-10-28.lcd

4. HRMS Spectrum

269-2

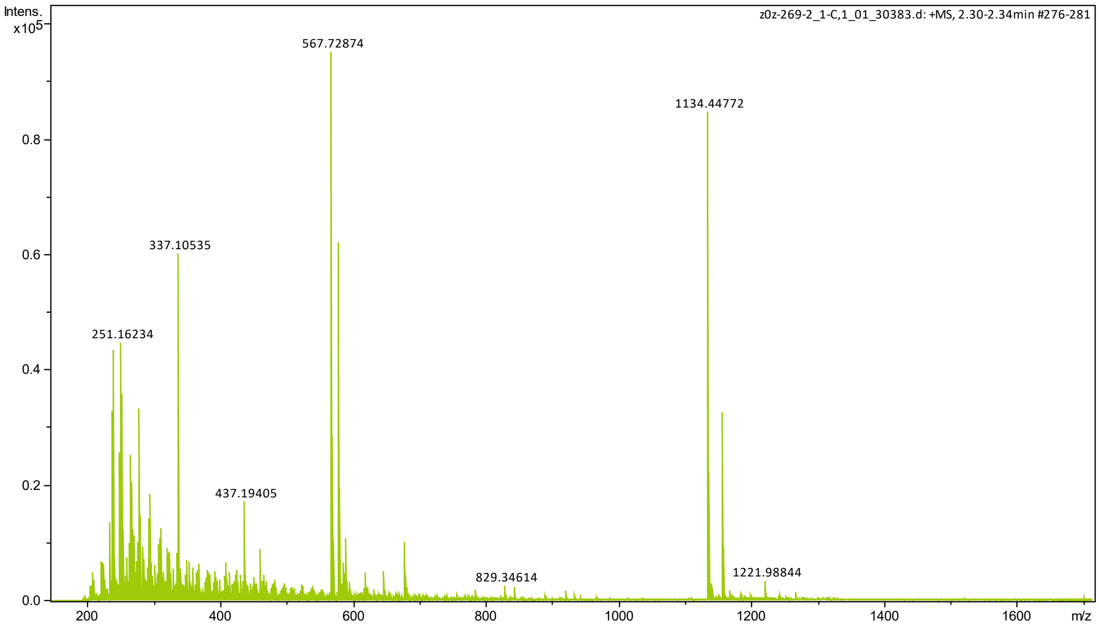

269-6

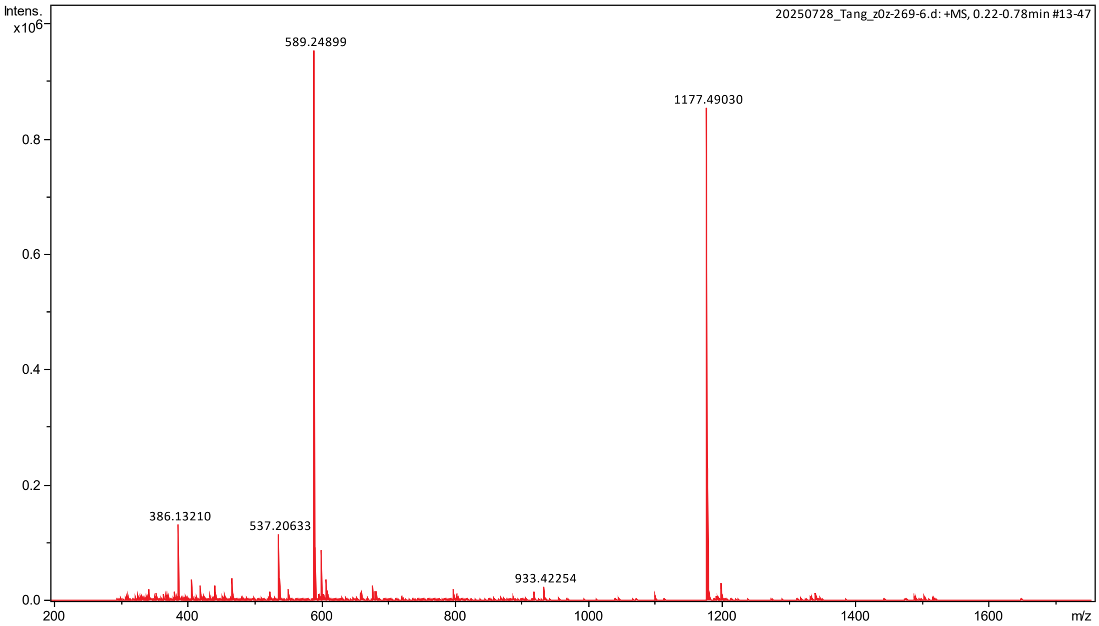

269-8

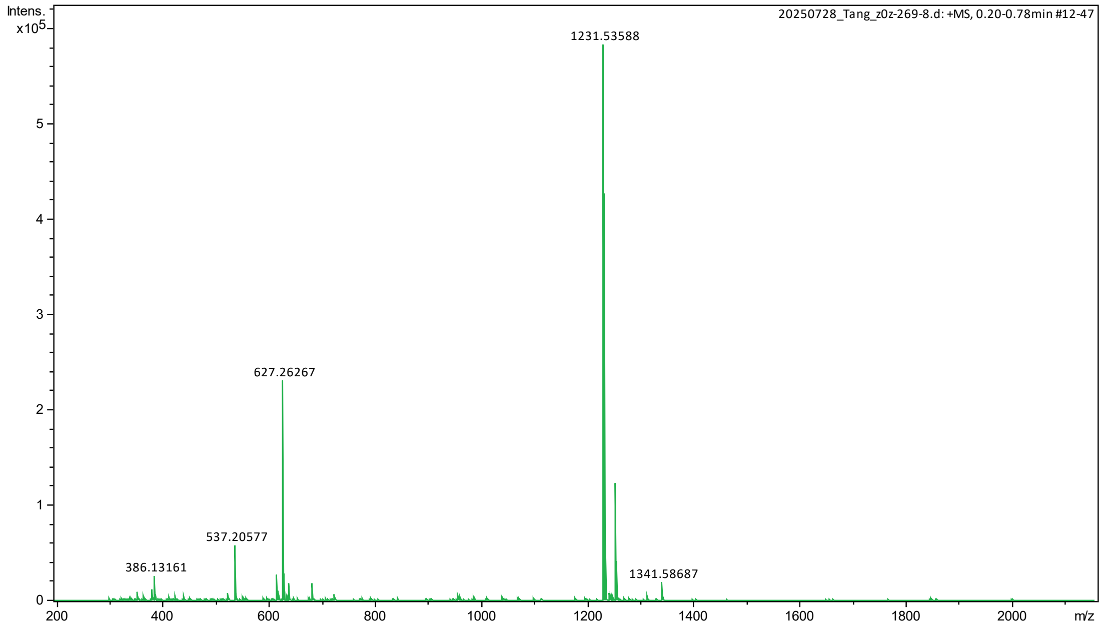
Intens.
x10^5
20250728_Tang_z0z-269-8.d: +MS, 0.20-0.78min #12-47
1231.53588
627.26267
537.20577
386.13161
1341.58687
0
1
2
3
4
5
200
400
600
800
1000
1200
1400
1600
1800
2000
m/z

269-10

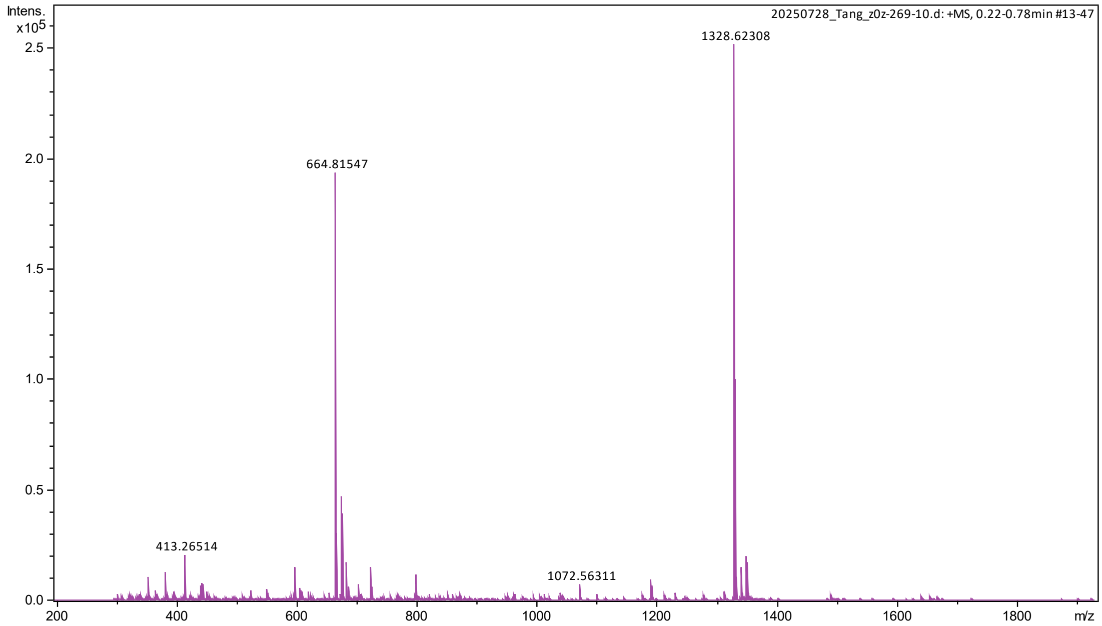
Intens.
x10^5
20250728_Tang_z0z-269-10.d: +MS, 0.22-0.78min #13-47
1328.62308
664.81547
413.26514
1072.56311
0.0
0.5
1.0
1.5
2.0
2.5
200
400
600
800
1000
1200
1400
1600
1800
m/z

References

1. P. Soares, M. S. Gadd, J. Frost, C. Galdeano, L. Ellis, O. Epemolu, S. Rocha, K. D. Read and A. Ciulli, Journal of Medicinal Chemistry **61** (2), 599–618 (2018).
2. P. A. Harris, D. Bandyopadhyay, S. B. Berger, N. Campobasso, C. A. Capriotti, J. A. Cox, L. Dare, J. N. Finger, S. J. Hoffman, K. M. Kahler, R. Lehr, J. D. Lich, R. Nagilla, R. T. Nolte, M. T. Ouellette, C. S. Pao, M. C. Schaeffer, A. Smallwood, H. H. Sun, B. A. Swift, R. D. Totoritis, P. Ward, R. W. Marquis, J. Bertin and P. J. Gough, ACS Medicinal Chemistry Letters **4** (12), 1238–1243 (2013).
3. A. Waterhouse, M. Bertoni, S. Bienert, G. Studer, G. Tauriello, R. Gumienny, F. T. Heer, T. A P. de Beer, C. Rempfer, L. Bordoli, R. Lepore and T. Schwede, Nucleic Acids Research **46** (W1), W296–W303 (2018).
4. R. Anandakrishnan, B. Aguilar and A. V. Onufriev, Nucleic Acids Research **40** (W1), W537–W541 (2012).
5. M. H. M. Olsson, C. R. Søndergaard, M. Rostkowski and J. H. Jensen, Journal of Chemical Theory and Computation **7** (2), 525–537 (2011).
6. O. Trott and A. J. Olson, Journal of Computational Chemistry **31** (2), 455–461 (2010).
7. S. Rosignoli and A. Paiardini, Bioinformatics **38** (17), 4233–4234 (2022).
8. Y. Yan, H. Tao, J. He and S.-Y. Huang, Nature Protocols **15** (5), 1829–1852 (2020).
9. J. A. Maier, C. Martinez, K. Kasavajhala, L. Wickstrom, K. E. Hauser and C. Simmerling, Journal of Chemical Theory and Computation **11** (8), 3696–3713 (2015).
10. P. Mark and L. Nilsson, The Journal of Physical Chemistry A **105** (43), 9954–9960 (2001).
11. J. Wang, R. M. Wolf, J. W. Caldwell, P. A. Kollman and D. A. Case, Journal of Computational Chemistry **25** (9), 1157–1174 (2004).
12. M. J. Frisch, G. W. Trucks, H. B. Schlegel, G. E. Scuseria, M. A. Robb, J. R. Cheeseman, G. Scalmani, V. Barone, G. A. Petersson, H. Nakatsuji, X. Li, M. Caricato, A. V. Marenich, J. Bloino, B. G. Janesko, R. Gomperts, B. Mennucci, H. P. Hratchian, J. V. Ortiz, A. F. Izmaylov, J. L. Sonnenberg, Williams, F. Ding, F. Lipparini, F. Egidi, J. Goings, B. Peng, A. Petrone, T. Henderson, D. Ranasinghe, V. G. Zakrzewski, J. Gao, N. Rega, G. Zheng, W. Liang, M. Hada, M. Ehara, K. Toyota, R. Fukuda, J. Hasegawa, M. Ishida, T. Nakajima, Y. Honda, O. Kitao, H. Nakai, T. Vreven, K. Throssell, J. A. Montgomery Jr., J. E. Peralta, F. Ogliaro, M. J. Bearpark, J. J. Heyd, E. N. Brothers, K. N. Kudin, V. N. Staroverov, T. A. Keith, R. Kobayashi, J. Normand, K. Raghavachari, A. P. Rendell, J. C. Burant, S. S. Iyengar, J. Tomasi, M. Cossi, J. M. Millam, M. Klene, C. Adamo, R. Cammi, J. W. Ochterski, R. L. Martin, K. Morokuma, O. Farkas, J. B. Foresman and D. J. Fox, (Wallingford, CT, 2016).
13. S. Grimme, S. Ehrlich and L. Goerigk, Journal of Computational Chemistry **32** (7), 1456–1465 (2011).
14. D. A. Case, H. M. Aktulga, K. Belfon, D. S. Cerutti, G. A. Cisneros, V. W. D. Cruzeiro, N. Forouzesh, T. J. Giese, A. W. Götz, H. Gohlke, S. Izadi, K. Kasavajhala, M. C. Kaymak, E. King, T. Kurtzman, T.-S. Lee, P. Li, J. Liu, T. Luchko, R. Luo, M. Manathunga, M. R. Machado, H. M. Nguyen, K. A. O'Hearn, A. V. Onufriev, F. Pan, S. Pantano, R. Qi, A. Rahnamoun, A. Risheh, S. Schott-Verdugo, A. Shajan, J. Swails, J. Wang, H. Wei, X. Wu, Y. Wu, S. Zhang, S. Zhao, Q. Zhu, T. E. Cheatham, III, D. R. Roe, A. Roitberg, C. Simmerling, D. M. York, M. C. Nagan and K. M. Merz, Jr., Journal of Chemical Information and Modeling **63** (20), 6183–6191 (2023).
15. T. Darden, D. York and L. Pedersen, The Journal of Chemical Physics **98** (12), 10089–10092 (1993).
16. M. J. Abraham, T. Murtola, R. Schulz, S. Páll, J. C. Smith, B. Hess and E. Lindahl, SoftwareX **1-2**, 19–25 (2015).
17. B. Hess, H. Bekker, H. J. C. Berendsen and J. G. E. M. Fraaije, Journal of Computational Chemistry **18** (12), 1463–1472 (1997).
18. G. Bussi, D. Donadio and M. Parrinello, The Journal of Chemical Physics **126** (1), 014101 (2007).
19. H. J. C. Berendsen, J. P. M. Postma, W. F. van Gunsteren, A. DiNola and J. R. Haak, The Journal of Chemical Physics **81** (8), 3684–3690 (1984).
20. P. Eastman, R. Galvelis, R. P. Peláez, C. R. A. Abreu, S. E. Farr, E. Gallicchio, A. Gorenko, M. M. Henry, F. Hu, J. Huang, A. Krämer, J. Michel, J. A. Mitchell, V. S. Pande, J. P. Rodrigues, J. Rodriguez-Guerra, A. C. Simmonett, S. Singh, J. Swails, P. Turner, Y. Wang, I. Zhang, J. D. Chodera, G. De Fabritiis and T. E. Markland, The Journal of Physical Chemistry B **128** (1), 109–116 (2024).
21. Z. Zhang, X. Liu, K. Yan, M. E. Tuckerman and J. Liu, The Journal of Physical Chemistry A **123** (28), 6056–6079 (2019).
22. M. Shirts and V. S. Pande, Science **290** (5498), 1903–1904 (2000).
23. V. A. Voelz, V. S. Pande and G. R. Bowman, Biophysical Journal **122** (14), 2852–2863 (2023).
24. B. E. Husic and V. S. Pande, Journal of the American Chemical Society **140** (7), 2386–2396 (2018).
25. J.-H. Prinz, H. Wu, M. Sarich, B. Keller, M. Senne, M. Held, J. D. Chodera, C. Schütte and F. Noé, The Journal of Chemical Physics **134** (17) (2011).
26. F. Nuske, B. G. Keller, G. Perez-Hernandez, A. S. Mey and F. Noe, J Chem Theory Comput **10** (4), 1739–1752

(2014).
27. M. P. Harrigan, M. M. Sultan, C. X. Hernandez, B. E. Husic, P. Eastman, C. R. Schwantes, K. A. Beauchamp, R. T. McGibbon and V. S. Pande, Biophys J **112** (1), 10–15 (2017).
28. G. R. Bowman, X. Huang and V. S. Pande, Methods **49** (2), 197–201 (2009).
29. M. K. Scherer, B. Trendelkamp-Schroer, F. Paul, G. Perez-Hernandez, M. Hoffmann, N. Plattner, C. Wehmeyer, J. H. Prinz and F. Noe, J Chem Theory Comput **11** (11), 5525–5542 (2015).
30. F. Litzinger, L. Boninsegna, H. Wu, F. Nuske, R. Patel, R. Baraniuk, F. Noe and C. Clementi, J Chem Theory Comput **14** (5), 2771–2783 (2018).
31. C. R. Schwantes and V. S. Pande, Journal of Chemical Theory and Computation **9** (4), 2000–2009 (2013).
32. G. Pérez-Hernández, F. Paul, T. Giorgino, G. De Fabritiis and F. Noé, The Journal of Chemical Physics **139** (1) (2013).
33. Y. Naritomi and S. Fuchigami, The Journal of Chemical Physics **139** (21) (2013).
34. S. Lloyd, IEEE Transactions on Information Theory **28** (2), 129–137 (1982).
35. R. T. McGibbon and V. S. Pande, J Chem Phys **142** (12), 124105 (2015).
36. A. J. Dominic, 3rd, T. Sayer, S. Cao, T. E. Markland, X. Huang and A. Montoya-Castillo, Proc Natl Acad Sci U S A **120** (12), e2221048120 (2023).
37. S. Cao, Y. Qiu, M. L. Kalin and X. Huang, The Journal of Chemical Physics **159** (13) (2023).
38. A. J. Dominic, 3rd, S. Cao, A. Montoya-Castillo and X. Huang, J Am Chem Soc **145** (18), 9916–9927 (2023).
39. S. Cao, A. Montoya-Castillo, W. Wang, T. E. Markland and X. Huang, The Journal of Chemical Physics **153** (1) (2020).
40. P. Deuflhard and M. Weber, Linear Algebra and its Applications **398**, 161–184 (2005).
41. S. Röblitz and M. Weber, Adv. Data Anal. Classif. **7** (2), 147–179 (2013).
42. S. Kube and M. Weber, The Journal of Chemical Physics **126** (2) (2007).
43. Y. Wu, S. Cao, Y. Qiu and X. Huang, The Journal of Chemical Physics **160** (12) (2024).
44. Robert T. McGibbon, Kyle A. Beauchamp, Matthew P. Harrigan, C. Klein, Jason M. Swails, Carlos X. Hernández, Christian R. Schwantes, L.-P. Wang, Thomas J. Lane and Vijay S. Pande, Biophysical Journal **109** (8), 1528–1532 (2015).
45. N. Michaud-Agrawal, E. J. Denning, T. B. Woolf and O. Beckstein, Journal of Computational Chemistry **32** (10), 2319–2327 (2011).
46. L. Schrödinger.
47. RDKit.
48. A. D. Gabriele Corso, Benjamin Fry, Nicholas Polizzi, Regina Barzilay, Tommi Jaakkola, in *International Conference on Learning Representations (ICLR)* (2024).
49. M. Parrinello and A. Rahman, J. Appl. Phys. **52** (12), 7189–7190 (1981).
50. M. S. Valdés-Tresanco, M. E. Valdés-Tresanco, P. A. Valiente and E. Moreno, Journal of Chemical Theory and Computation **17** (10), 6281–6291 (2021).
51. J. Wang, Q. Cai, Y. Xiang and R. Luo, Journal of Chemical Theory and Computation **8** (8), 2741–2751 (2012).
52. C. Tan, Y.-H. Tan and R. Luo, The Journal of Physical Chemistry B **111** (42), 12263–12274 (2007).
53. R. Nunes, D. Vila-Viçosa and P. J. Costa, Journal of Chemical Theory and Computation **15** (7), 4241–4251 (2019).
54. S. Passaro, G. Corso, J. Wohlwend, M. Reveiz, S. Thaler, V. R. Somnath, N. Getz, T. Portnoi, J. Roy, H. Stark, D. Kwabi-Addo, D. Beaini, T. Jaakkola and R. Barzilay, bioRxiv (2025).
55. H. Xie, M. S. Bacabac, M. Ma, E.-J. Kim, Y. Wang, W. Wu, L. Li, W. Xu and W. Tang, Journal of Medicinal Chemistry **66** (18), 13028–13042 (2023).
56. Z. Zhang, C. Li, N. J. Hawkins, R. Mudududdla, Y. Nie, P.-K. Liu, P. Huang, N. M. Del Rio, H. Chang, M. E. Brown, L. Li and W. Tang, Journal of Medicinal Chemistry **68** (14), 15120–15136 (2025).
